# Risk factors of transient and permanent hypoparathyroidism after thyroidectomy: a systematic review and meta-analysis

**DOI:** 10.1097/JS9.0000000000001475

**Published:** 2024-04-23

**Authors:** Kang Ning, Yongchao Yu, Xinyi Zheng, Zhenyu Luo, Zan Jiao, Xinyu Liu, Yiyao Wang, Yarong Liang, Zhuoqi Zhang, Xianglin Ye, Weirui Wu, Jian Bu, Qiaorong Chen, Fuxiang Cheng, Lizhen Liu, Mingjie Jiang, Ankui Yang, Tong Wu, Zhongyuan Yang

**Affiliations:** a Department of Head and Neck Surgery, Sun Yat-sen University Cancer Center; b State Key Laboratory of Oncology in South China, Guangdong Provincial Clinical Research Center for Cancer, Sun Yat-sen University Cancer Center; c Zhongshan School of Medicine, Sun Yat-sen University, Guangzhou; d Clinical Medical College, Southwest Medical University; e Faculty of Nursing, Southwest Medical University, Luzhou, People’s Republic of China

**Keywords:** hypoparathyroidism, meta-analysis, risk factor, systematic review, thyroidectomy

## Abstract

**Background:**

Postoperative hypoparathyroidism (hypoPT) is a common complication following thyroid surgery. However, current research findings on the risk factors for post-thyroid surgery hypoPT are not entirely consistent, and the same risk factors may have different impacts on transient and permanent hypoPT. Therefore, there is a need for a comprehensive study to summarize and explore the risk factors for both transient and permanent hypoPT after thyroid surgery.

**Materials and methods:**

Two databases (PubMed and Embase) were searched from inception to 2024. The Newcastle–Ottawa Scale was used to rate study quality. Pooled odds ratios were used to calculate the relationship of each risk factor with transient and permanent hypoPT. Subgroup analyses were conducted for hypoPT with different definition-time (6 or 12 months). Publication bias was assessed using Begg’s test and Egger’s test.

**Results:**

A total of 19 risk factors from the 93 studies were included in the analysis. Among them, sex and parathyroid autotransplantation were the most frequently reported risk factors. Meta-analysis demonstrated that sex (female vs. male), cN stage, central neck dissection, lateral neck dissection, extent of central neck dissection (bilateral vs. unilateral), surgery [total thyroidectomy (TT) vs. lobectomy], surgery type (TT vs. sub-TT), incidental parathyroidectomy, and pathology (cancer vs. benign) were significantly associated with transient and permanent hypoPT. Preoperative calcium and parathyroid autotransplantation were only identified as risk factors for transient hypoPT, while preoperative PTH was a protective factor. Additionally, node metastasis and parathyroid in specimen were associated with permanent hypoPT.

**Conclusion:**

The highest risk of hypoPT occurs in female thyroid cancer patients with lymph node metastasis undergoing TT combined with neck dissection. The key to preventing postoperative hypoPT lies in the selection of surgical approach and intraoperative protection.

## Introduction

Highlights

- This is the first comprehensive meta-analysis describing the risk factors of hypoparathyroidism (hypoPT) after thyroid surgery.
- The highest risk of hypoPT occurs in female thyroid cancer patients with lymph node metastasis undergoing total thyroidectomy combined with neck dissection.
- Optimal prevention of postoperative hypoPT hinges on carefully choosing the surgical approach and ensuring meticulous intraoperative protection.

Postoperative hypoparathyroidism (hypoPT) is the most common complication of thyroid surgery^1^. According to a meta-analysis, the median incidence of temporary and permanent hypoPT following thyroidectomy ranges from 19 to 38% and 0 to 3%, respectively^2^. HypoPT typically arises from intraoperative damage or removal of the parathyroid glands, leading to decreased levels of parathyroid hormone (PTH) in the blood, subsequently triggering symptoms of hypocalcemia^3,4^. Patients may experience muscle spasms, tetany, and cardiac arrhythmias, ultimately diminishing their quality of life^5,6^. Therefore, exploring the risk factors associated with postoperative hypoPT is crucial for providing enhanced healthcare, which can improve surgical safety, enhance postoperative recovery, and reduce long-term health risks for patients.

Recent studies have identified various risk factors for post-thyroidectomy hypoPT, including age, operation type, disease type, parathyroid autotransplantation (PA)^7–11^. However, disparities in sample sizes, study designs, outcome definitions, and analysis methods of those studies could result in divergent conclusions. For example, some studies have found that sex was an independent risk factor for hypoPT^7–9^, while other studies have failed to yield similar results^10,11^. Central node dissection (CND) is considered an important risk factor for post-thyroidectomy hypoPT^1^, but there were still some negative results^12^. Additionally, the same risk factor may have different effects on temporary and permanent hypoPT, which can affect clinical decision-making^13,14^.

Several meta-analyses have been conducted to explore post-thyroidectomy hypoPT^15–17^. Koimtzis *et al*.^15^ suggested that variations in permanent hypoPT definitions (diagnosed at 6 or 12 months postoperatively) may not yield statistically significant differences in the overall incidence rate, without delving into the potential risk factors. Besides, Vaitsi *et al*.^16^ confirmed preoperative vitamin D deficiency as a risk factor for both temporary and permanent hypoPT, while Chen *et al*.^17^ identified 12 risk factors predictive of postoperative hypocalcemia. However, above-mentioned studies only focused on individual risk factors^16^ or specific symptoms of hypoPT^17^. Therefore, a more comprehensive summary and discussion are still needed for the risk factors of post-thyroidectomy hypoPT.

In this systematic review and meta-analysis, data from 93 studies were synthesized to delineate all risk factors associated with transient and permanent hypoPT following thyroid surgery. The comprehensive identification of risk factors for hypoPT will help surgeon develop strategies for prevention and management of postoperative complication in patients undergoing thyroidectomy.

## Method

### Protocol registration

This meta-analysis was performed in accordance with the Preferred Reporting Items for Systematic Reviews and Meta-Analyses (PRISMA, Supplemental Digital Content 1, http://links.lww.com/JS9/C438, Supplemental Digital Content 2, http://links.lww.com/JS9/C439) and Assessing the Methodological Quality of Systematic Reviews (AMSTAR, Supplemental Digital Content 3, http://links.lww.com/JS9/C440) guidelines^18,19^. The protocol of this study has been registered with the International Prospective Register of Systematic Reviews prior to the initiation of data extraction (http://www.crd.york.ac.uk/PROSPERO, registration no. CRD 42023412216 accessed on 21 January 2024).

### Search strategy

Electronic databases including PubMed and Embase were thoroughly searched from inception up to July 2023. Search strategies were developed using controlled vocabulary terms [Medical Subject Headings (MeSH) terms for PubMed, and EMTREE headings for EMBASE] as well as a range of relevant keywords, including ‘Risk Factors’, ‘Thyroid Gland’, ‘General Surgery’, ‘Thyroidectomy’, ‘Thyroid removal’, ‘Thyroid resection’, ‘Hypoparathyroidism’, ‘Vitamin D’, ‘Hypocalcemia’, ‘Calcium’, ‘Parathyroid Hormone’, and ‘PTH’. Detailed lists of the search terms for each database are available in Supplementary Material (Tables S1A, B, Supplemental Digital Content 4, http://links.lww.com/JS9/C441).

### Eligible criteria

Studies were included if they met the following criteria: (1) published in English; (2) prospective or retrospective cohort of adult patients who underwent open thyroid surgery for the first time; (3) reported the associations between the potential risk factors and hypoPT; (4) provided a precise definition of transient or permanent hypoPT; (5) the odds ratio (OR) and corresponding 95% CIs could be either calculated or extracted directly; Exclusion criteria were as follows: (1) the cohort included children, endoscopic surgery, or second surgeries; (2) studies only reported hypoPT in a short time after surgery; (3) studies that lacked sufficient information to assess the impact of the potential risk factors on hypoPT; (4) unpublished studies and nonpeer reviewed data (e.g. conference abstracts); (5) studies that did not provide a precise definition of hypoPTH (including both timing and criteria) were excluded from consideration. The criteria for including risk factors in the study are as follows: (1) Consistent definition and format across different literature sources. (2) Reported in at least three studies (including permanent and transient hypoPT). (3) Suitable for meta-analysis integration.

The outcomes of all included studies focused on transient or permanent hypoPT during postoperative follow-up, rather than around the perioperative period. The definition of hypoPT included the following criteria: (1) persistent low levels of PTH or calcium after surgery; (2) the need for oral calcium or vitamin D supplementation after surgery; (3) clinical symptoms of hypocalcemia, such as seizures and muscle spasms after surgery; (4) HypoPT that persisted beyond 6 or 12 months postoperatively was classified as permanent, whereas cases resolving within this timeframe were categorized as transient.

### Data extraction

Following the elimination of duplicate records, all titles and abstracts underwent a preliminary screening. Full-text articles of potentially eligible studies were retrieved for further assessment. The reference lists of all retrieved articles and relevant reviews were manually searched to identify additional eligible studies. The following research data were extracted from each eligible articles: first author name, publication year, study location, total sample size, number of male participants, average age, study design, number of transient or permanent hypoPT cases, definition of transient or permanent hypoPT, the number of participants with malignancy, CND, lateral neck dissection (LND), and PA. To comprehensively analyze hypoPT, univariate and multivariate analysis results (OR and 95% CI) for reported risk factors in each study were systematically documented. All recorded risk factor data were compiled and, when similar risk factors were identified across different studies, they were categorized into the same group. Only risk factors reported in three or more studies were included in this meta-analysis, and the screening of risk factors were shown in Table S2A-D and S3A-E (Supplemental Digital Content 4, http://links.lww.com/JS9/C441). In cases where the results of a single study were reported in multiple articles, we prioritized the latest publication to avoid the potential overlap in study populations.

### Quality assessment

A nine-score system of the Newcastle–Ottawa Quality Assessment Scale (NOS) was applied to evaluate the quality of included studies across three domains: selection of study groups (0–4 scores), comparability of groups (0–2 scores), and ascertainment of exposure or outcomes (0–3 scores)^20^. A total score of 0–3, 4–6, and 7–9 was considered to indicate low, moderate, and high quality, respectively. Study selection, data extraction, and quality assessment were independently conducted by two experienced investigators (K.N. and X.Z.) and any discrepancies were resolved through discussions with a senior reviewer (Z.Y.). The detail results of quality assessment were shown in Table S4A-J (Supplemental Digital Content 4, http://links.lww.com/JS9/C441). Furthermore, we assessed the quality of meta-analysis of each risk factor using the Grading of Recommendations Assessment, Development, and Evaluation (GRADE) approach based on study design, risk of bias, inconsistency, indirectness, imprecision, and publication bias^21,22^.

### Statistical analysis

For univariable analysis results of risk factors with the same content and format in the included studies, we conducted a meta-analysis to analyze the combined OR values and 95% CI. The results were visually presented using forest plots and tables. Given the current existence of two different definitions for transient and permanent hypoPT, which are defined at 6 months and 12 months, we conducted subgroup meta-analyses for these two definitions. Heterogeneity between included studies was assessed using *I*-squared (*I* ^2^) statistic. In case of high statistical heterogeneity (*I* ^2^>50%), random-effect models were employed to pool effect size; otherwise, fixed-effect models were adopted^23^. Publication bias was assessed using Begg’s test, and Egger’s test. *P*-value of <0.05 was considered statistically significant. All statistical analyses were performed using the ‘meta’ package of R version 4.3.0.

## Results

### Literature search

**Figure 1:**
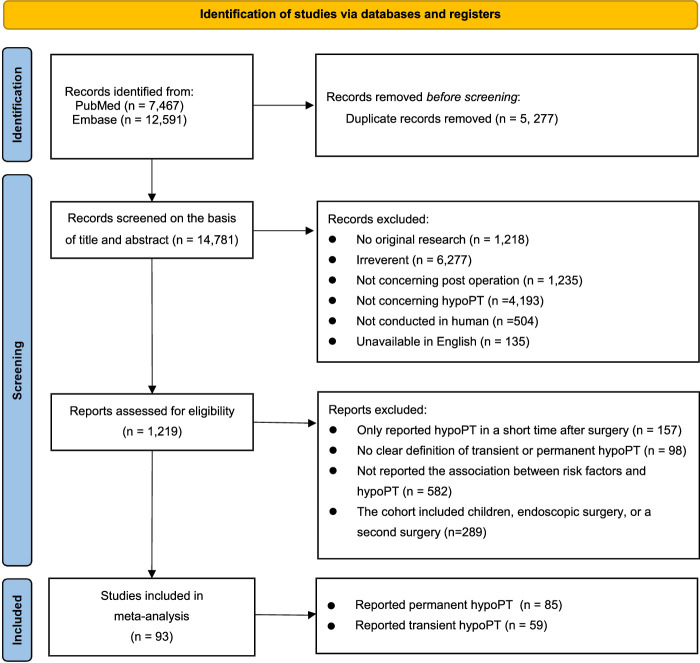
PRISMA diagram showing screening and selection of studies for systematic review and meta-analysis. hypoPT, hypoparathyroidism.

Initially, a total of 20 058 possibly relevant studies from two electronic databases were identified. After removal of 5777 duplicate records, 14 781 titles and abstracts remained for further screening. 13 562 studies were excluded for various reasons [not original research (*n*=1218); irreverent (*n*=6277); not concerning postoperation (*n*=1235); not concerning hypoPT (*n*=4193); not conducted in human (*n*=504); unavailable in English (*n*=135)]. Then, 1219 studies were further excluded in full-text screening. Among these, 157 studies exclusively reported hypoPT in a short period following surgery, 98 studies did not provide clear definition of transient or permanent hypoPT, 582 studies did not report the association between risk factors and hypoPT, 289 studies included children, endoscopic surgery, or a second surgery. In total, 93 studies met our eligibility requirements and were included in the final analysis^8–14,24–109^ (Fig. 1).

### Study characteristics

The characteristics and quality assessment of included studies are presented in Table 1. The number of participants in these studies ranged from 40 to 192 333, with the percentage of male participants ranging from 0 to 89.5%. The average age of patients varied from 30.7 to 57.0 years, and 30 articles did not mention the average age. In terms of study design, there were 65 retrospective cohort studies, 28 prospective cohort studies included in the analysis. Eighty-five studies reported permanent hypoPT, and only 59 included studies reported transient hypoPT. The incidence of transient hypoPT in different studies ranges from 1.0 to 58.0%, while the incidence of permanent hypoPT falls between 0 and 29.2%. In terms of defining hypoPT, 16 studies relied solely on serum PTH or calcium levels, 31 studies determined it based on the need for calcium and vitamin D supplementation, and 46 studies included both criteria. Additionally, 57 studies used a 6-month definition time, while 36 studies used a 12-month definition time for hypoPT. The methodological quality of included studies ranged from moderate to high with a mean NOS score of 8 (range 6–9).

**Table 1:** Characteristics of included studies.

					HypoPT definition^a^								
Author (y)	Country	Total (male)	Age mean	Study Design	Details	Time (months)	Transient hypoPT n (%)	Permanent hypoPT n (%)	Malignancy n (%)	CND n (%)	LND n (%)	PA n (%)	NOS
Annebäck (2024)^25^	Sweden	1636 (16.3)	45.0	Retrospective	Calcium/vitamin D supplementation	12	—	102 (6.2)	41 (2.5)	—	—	289 (17.7)	8
Eynde (2023)^26^	Belgium	2030 (21.2)	—	Prospective	1. Calcium<2.0 mmol/l 2. Calcium/vitamin D supplementation	6	273 (13.4)	67 (3.3)	138 (6.8)	—	—	—	8
Issa (2023)^24^	USA	310 (19.7)	53.0	Retrospective	Below normal calcium levels	6	7 (2.3)	—	235 (75.8)	—	—	—	7
Ahn (2022)^30^	Korea	101 (23.8)	52.7	Prospective	Calcium supplementation	6	8 (7.9)	—	87 (86.1)	51 (50.5)	—	—	9
Baud (2022)^13^	France	1547 (22.9)	49.1	Retrospective	1.Calcium<2.0 mmol/l 2.Calcium/vitamin D supplementation	12	209 (13.5)	26 (1.7)	—	—	535 (34.6)	—	8
Lončar (2022)^29^	Netherlands	749 (24.4)	50.0	Retrospective	Vitamin D supplementation	12	—	53 (7.1)	445 (59.4)	—	—	52 (6.9)	7
Riordan (2022)^28^	Ireland	511 (12.5)	—	Retrospective	1. Calcium< 2.0 mmol/l 2. Calcium/vitamin D supplementation	6	—	16 (3.1)	101 (19.8)	—	—	—	7
Takahashi (2022)^27^	Japan	2388 (26.6)	45.0	Retrospective	Vitamin D supplementation	12	—	484 (20.3)	1570 (65.7)	—	—	—	8
Xu (2022)^14^	China	121 (0)	39.0	Retrospective	PTH<11.0 pg/ml/ Calcium<2.0 mmol/l	6	66 (54.5)	8 (6.6)	—	—	—	68 (56.2)	7
Annebäck (2021)^35^	Sweden	4493 (29.6)	—	Retrospective	Calcium/vitamin D supplementation	12	—	983 (21.9)	2734 (60.9)	—	—	1672 (37.2)	7
Lui (2021)^33^	China	4123 (17.9)	49.0	Prospective	Calcium/vitamin D supplementation	6	—	460 (11.2)	—	—	—	—	7
Palmhag (2021)^32^	Sweden	366 (13.1)	43.1	Retrospective	1.PTH<10.0 pg/ml/Calcium<1.2 mmol/l 2.Hypocalcemic symptoms 3.Calcium/vitamin D supplementation	12	46 (12.7)	12 (3.3)	—	—	—	63 (17.2)	8
Qiu (2021)^12^	China	1749 (28.1)	41.5	Retrospective	1. Below normal PTH levels 2.Hypocalcemic symptoms	6	363 (20.8)	72 (4.1)	—	403 (23.0)	373 (21.3)	—	7
Salem (2021)^31^	Sweden	722 (21.3)	47.0	Retrospective	Calcium supplementation	6	—	93 (12.9)	435 (60.2)	457 (63.3)	—	333 (46.1)	8
Bergenfelz (2020)^36^	Sweden	4828 (16.8)	46.2	Retrospective	Vitamin D supplementation	6	—	239 (5.0)	209 (4.3)	—	—	—	7
Di (2020)^37^	Italy	586 (21.3)	48.0	Retrospective	Calcium/vitamin D supplementation	12	243 (58.0)	22 (5.2)	586 (100)	—	—	—	8
Godlewska (2020)^38^	Poland	89 (22.5)	46.6	Prospective	PTH< 12.0 pg/ml	12	—	12 (13.5)	89 (100)	—	11 (12.4)	12 (13.5)	7
Jørgensen (2020)^39^	Denmark	187 (12.3)	53.6	Retrospective	1. Below normal PTH levels 2. Calcium<1.2 mmol/l 3.Vitamin D supplementation	6	81 (43.3)	25 (13.4)	—	—	—	24 (12.8)	8
Karunakaran (2020)^34^	India	328 (19.8)	34.0	Prospective	1. Calcium<2.0 mmol/l 2.Calcium supplementation	6	187 (57.0)	26 (7.9)	—	—	—	13 (4.0)	7
Song (2020)^40^	China	129 (31.8)	—	Retrospective	1. Calcium<2.0 mmol/L 2. Calcium/vitamin D supplementation	12	39 (30.2)	3 (2.3)	—	—	—	—	8
Wu (2020)^10^	USA	184 (34.8)	—	Retrospective	1.PTH<10.0 pg/ml; 2. Calcium/vitamin D supplementation	6	—	6 (3.3)	139 (75.5)	105 (57.1)	64 (34.8)	81 (44.0)	6
Zheng (2020)^41^	China	546 (28.9)	50.9	Retrospective	1. PTH<15.0 pg/ml 2. Calcium/vitamin D supplementation	6	—	22 (4.0)	340 (62.3)	—	—	—	9
Díez (2019)^42^	Spain	1792 (21.1)	48.5	Retrospective	Calcium/ vitamin D supplementation	12	—	300 (16.7)	1453 (81.1)	—	—	146 (8.1)	8
Gambardella (2019)^43^	Italy	371 (26.7)	—	Retrospective	1. Below normal PTH levels 2. Calcium/vitamin D supplementation	6	61 (16.4)	5 (1.3)	125 (33.7)	187(50.4)	—	—	8
Imga (2019)^44^	Turkey	933 (19.8)	—	Retrospective	1. PTH<13.0 pg/mL 2. Calcium/vitamin D supplementation	12	192 (20.6)	20 (2.1)	94 (10.1)	—	117 (12.5)	—	7
Jeon (2019)^45^	Korea	255 (11.4)	49.2	Retrospective	Calcium supplementation	12	11 (4.3)	—	255 (100)	—	—	—	7
Kong (2019)^46^	China	226 (11.9)	—	Prospective	1. PTH<15.0 pg/mL 2. Calcium/vitamin D supplementation	6	56 (24.8)	—	226 (100)	—	177 (78.3)	17 (7.5)	8
Kwon (2019)^47^	Korea	168 (14.9)	43.1	Retrospective	1. Calcium<2.0 mmol/L 2. Calcium/vitamin D supplementation	6	31 (18.5)	—	93 (55.4)	—	—	—	9
León-Ballesteros (2019)^48^	Mexico	93 (19.4)	46.8	Retrospective	Vitamin D supplementation or magnesium	12	—	37 (3.9)	642 (67.2)	459 (48.0)	—	—	8
Maurer (2019)^49^	Germany	205 (19.5)	43.0	Prospective	Below normal PTH levels	6	41 (20.0)	6 (2.9)	—	—	—	—	8
Nicholson (2019)^50^	USA	300 (23.0)	52.7	Retrospective	Vitamin D supplementation	6	3 (1.0)	—	14 (4.7)	—	—	—	7
Sugino (2019)^51^	Japan	1476 (24.2)	39.0	Prospective	Calcium/vitamin D supplementation	6	324 (22.0)	49 (3.3)	—	—	—	—	9
Falch (2018)^11^	Germany	702 (28.6)	53.0	Retrospective	1. Calcium<1.9 mmol/L 2. Calcium/vitamin D supplementation	6	—	48 (6.8)	55 (7.8)	—	24 (3.4)	40 (5.7)	7
Su (2018)^52^	China	766 (26.9)	43.3	Retrospective	Below normal PTH levels	6	271 (35.8)	10 (1.3)	—	540 (71.4)	—	606 (80.2)	9
Teshima (2018)^53^	Japan	2388 (26.6)	45.0	Retrospective	Calcium/vitamin D supplementation	6	—	12 (0.5)	1570 (65.7)	—	—	—	7
Thomusch (2018)^54^	Germany	18955 (24.6)	—	Prospective	Calcium/vitamin D supplementation	6	1761 (9.3)	135 (0.7)	—	—	—	—	7
Vasileiadis (2018)^55^	Greece	2556 (20.7)	51.4	Retrospective	1.Below normal calcium levels 2. Calcium/vitamin D supplementation	12	461 (18.0)	88 (3.4)	848 (33.2)	—	—	469 (18.3)	8
Villarroya-Marquina (2018)^56^	Spain	142 (14.8)	—	Prospective	1. Below normal PTH levels 2. Calcium/vitamin D supplementation	6	—	36 (25.4)	42 (29.6)	34 (23.9)	10 (7.0)	—	8
Wang (2018)^57^	China	136 (23.5)	42.0	Retrospective	1. Below normal PTH levels 2. Calcium <2.0 mmol/l 3. Calcium/vitamin D supplementation	6	58 (42.6)	10 (7.4)	86 (63.2)	89 (65.4)	20 (14.7)	—	8
Yoo (2018)^58^	Korea	384 (16.4)	49.3	Retrospective	Below normal PTH levels	6	151 (39.3)	8 (2.1)	384 (100)	—	—	—	8
Kwon (2017)^65^	Korea	2031 (11.9)	47.0	Retrospective	1. Calcium<2.0 mmol/l 2. Calcium/vitamin D supplementation	12	170 (8.4)	23 (1.1)	—	—	—	—	7
Lin (2017)^64^	China	3186 (18.4)	47.1	Retrospective	1.PTH <14 pg/ml 2. Calcium/vitamin D supplementation	6	258 (8.1)	74 (2.3)	196 (6.2)	—	—	—	9
Lorente-Poch (2017)^63^	Spain	186 (15.1)	—	Prospective	1. PTH <13 pg/ml 2. Calcium supplementation	12	—	46 (24.7)	54 (29.0)	—	—	110 (59.1)	8
Serra (2017)^62^	Spain	170 (22.9)	47.0	Prospective	1. PTH <13 pg/ml 2. Calcium/vitamin D supplementation	12	—	11 (6.5)	—	170 (100)	48 (28.2)	41 (24.1)	7
Su (2017)^61^	China	903 (28.1)	43.2	Retrospective	Below normal PTH levels	6	399 (44.2)	10 (1.1)	4 (0.4)	753 (83.4)	—	579 (64.1)	7
Suwannasarn (2017)^60^	Thailand	65 (13.8)	43.0	Prospective	Calcium/vitamin D supplementation	6	—	19 (29.2)	42 (64.6)	—	—	—	8
Zheng (2017)^59^	China	548 (32.8)	41.8	Retrospective	Below normal PTH levels 2.Calcium/vitamin D supplementation	12	122 (22.3)	18 (3.3)	312 (56.9)	298 (54.4)	—	—	8
Dubernard (2016)^75^	France	295 (18.3)	47.6	Retrospective	Calcium supplementation	12	—	35 (11.9)	295 (100)	212 (71.9)	—	—	8
Garrahy (2016)^74^	Ireland	201 (–)	—	Retrospective	1.Below normal calcium levels 2. Calcium/vitamin D supplementation	6	—	10 (5.0)	70 (34.8)	32 (15.9)	—	37 (18.4)	8
He (2016)^73^	China	215 (26.5)	—	Retrospective	1. PTH <10 pg/ml 2. Calcium/vitamin D supplementation	6	11 (5.1)	11 (5.1)	56 (26.0)	—	—	—	7
Järhult (2016)^72^	Sweden	640 (14.5)	48.8	Prospective	Calcium/vitamin D supplementation	12	—	22 (3.4)	12 (1.9)	32 (5.0)	—	71 (11.1)	9
Jeong (2016)^8^	Korea	1030 (16.0)	—	Prospective	1.PTH <13 pg/mL 2. Calcium/vitamin D supplementation	6	291 (28.3)	27 (2.6)	909 (88.3)	822 (79.8)	100 (9.7)	—	7
Kim (2016)^71^	Korea	11569 (19.7)	—	Retrospective	PTH <5 pg/ml	6	509 (4.4)	55 (0.5)	11596 (100.2)	8735 (75.5)	—	—	8
Lang (2016)^9^	China	569 (21.3)	52.6	Prospective	Calcium supplementation or calcitriol	12	—	15 (2.6)	—	—	—	95 (16.7)	8
Lin (2016)^70^	China	1598 (16.4)	49.3	Retrospective	PTH<14 pg/ml	6	118 (7.4)	31(1.9)	205 (12.8)	—	—	—	7
Longheu (2016)^69^	Italy	788 (20.8)	—	Retrospective	PTH<10 pg/mL	6	210 (26.6)	11 (1.4)	64 (8.1)	—	47(6.0)	—	7
Park (2016)^68^	Korea	1411 (20.0)	46.1	Retrospective	PTH <10 pg/mL / Calcium<1.9 mmol/l 2. Calcium supplementation 3. Hypocalcemic symptoms	6	153 (10.8)	23 (1.6)	1351 (95.7)	—	280 (19.8)	219 (15.5)	7
Selberherr (2016)^67^	Austria	349 (24.1)	—	Prospective	1. Below normal PTH or calcium levels 2. Calcium/vitamin D supplementation	6	7 5(21.5)	4 (1.1)	—	112 (32.1)	—	—	9
Seo (2016)^66^	Korea	192333 (17.2)	48.0	Retrospective	Calcium supplementation	12	—	10007 (5.2)	19233 (10.0)	—	—	—	7
Carvalho (2015)^76^	Brazil	580 (89.5)	—	Retrospective	1. Calcium<2.0 mmol/l 2. Calcium/vitamin D supplementation	6	201 (34.7)	23 (4.0)	420 (72.4)	—	—	—	8
Daher (2015)^79^	France	3574 (–)	—	Prospective	1. Calcium<2.0 mmol/l 2. Calcium/vitamin D supplementation	6	737 (20.6)	78 (2.2)	—	—	—	—	8
Lorente-Poch (2015)^78^	Spain	657 (17.0)	—	Prospective	1.PTH <13 pg/ml 2.Calcium supplementation	12	—	30 (4.6)	—	117 (17.8)	54 (8.2)	143 (21.8)	9
Wang (2015)^77^	China	438 (19.6)	46.7	Retrospective	1. Calcium<2.0 mmol/L 2. Below normal PTH levels 3. Calcium/vitamin D supplementation	12	—	12 (2.7)	—	381 (87.0)	143 (32.6)	39 (8.9)	8
Ahn (2014)^88^	Korea	361 (14.7)	47.6	Retrospective	1. Calcium<2.0 mmol/l 2. Calcium supplementation	6	27 (7.5)	21 (5.8)	—	70 (19.4)	—	—	7
Almquist (2014)^87^	Sweden	519 (22.0)	46.0	Prospective	Calcium/vitamin D supplementation	12	—	10 (1.9)	100 (19.3)	—	—	90 (17.3)	7
Calo (2014)^86^	Italy	455 (21.3)	—	Retrospective	PTH <10 pg/ml	6	73 (16.0)	11 (2.4)	44 (9.7)	—	—	—	7
Nawrot (2014)^85^	Poland	401 (10.2)	50.8	Retrospective	Calcium/vitamin D supplementation	12	—	34 (8.5)	—	—	—	—	9
Praženica (2014)^84^	Czech Republic	1068 (13.3)	50.8	Retrospective	Calcium/vitamin D supplementation	12	185 (17.3)	16 (1.5)	104 (9.7)	71 (6.6)	—	79 (7.4)	6
Promberger (2014)^83^	Austria	2558 (22.3)	55.0	Prospective	1.Below normal calcium levels 2. Calcium/vitamin D supplementation	12	—	64 (2.5)	169 (6.6)	—	—	—	8
Puzziello (2014)^82^	Italy	2631 (21.9)	52.0	Prospective	Calcium<1.1 mmol/l	6	734 (27.9)	23 (0.9)	398 (15.1)	—	333 (12.7)	—	8
Song (2014)^81^	Korea	454 (21.6)	50.3	Retrospective	Below normal PTH levels	6	254 (55.9)	20 (4.4)	454 (100)	332 (73.1)	71 (15.6)	35 (7.7)	8
Wei (2014)^80^	China	477 (16.1)	45.7	Retrospective	1. PTH <15 pg/ml 2. Below normal calcium levels 3. Calcium/ supplementation	6	124 (26.0)	9 (1.9)	477 (100)	—	—	321 (67.3)	7
Barczyński (2013)^91^	Poland	640 (21.1)	52.7	Retrospective	1. PTH <10 pg/ml / Calcium<2.0 mmol/l 2. Calcium supplementation	12	146 (22.8)	10 (1.6)	640 (100)	358 (55.9)	—	—	9
Hammerstad (2013)^90^	Norway	40 (17.5)	—	Retrospective	1. Below normal PTH or calcium levels 2.Vitamin D supplementation	12	17 (42.5)	4 (10.0)	—	—	—	9 (22.5)	8
Paek (2013)^89^	Korea	531 (15.3)	45.5	Retrospective	Calcium/vitamin D supplementation	12	—	19 (3.6)	518 (97.6)	511 (96.2)	—	239 (45)	9
Abboud (2012)^95^	Lebanon	1127 (22.1)	57.0	Retrospective	Below normal PTH levels 2.Calcium/vitamin D supplementation	12	207 (18.4)	9 (0.8)	237 (21.0)	139 (12.3)	—	227 (20.1)	8
Barczyński (2012)^94^	Poland	191 (11.0)	—	Prospective	1. PTH <10 pg/mL 2. Calcium supplementation	12	37 (19.4)	1 (0.5)	9 (4.7)	—	—	16 (8.4)	8
Giordano (2012)^93^	Italy	1087 (–)	—	Retrospective	1. Calcium<2.0 mmol/l 2. Calcium/vitamin D supplementation	6	408 (37.5)	—	—	693 (63.8)	—	—	7
Sousa (2012)^92^	Brazil	333 (8.7)	45.0	Prospective	1.Below normal PTH levels 2. Calcium supplementation	6	—	14 (4.2)	—	—	—	—	8
Barczyński (2011)^97^	Poland	8106 (9.4)	—	Retrospective	1. PTH <10 pg/ml 2. Calcium supplementation	12	521 (6.4)	11 (0.1)	406 (5.0)	—	—	—	8
Wong (2011)^96^	China	329 (17.3)	30.7	Prospective	1. Below normal PTH levels 2.Calcium/vitamin D supplementation	12	76 (23.1)	14 (4.3)	—	—	—	—	9
Barczyński (2010)^101^	Poland	570 (9.3)	—	Retrospective	Below normal calcium levels	6	45 (7.9)	9 (1.6)	49 (8.6)	—	—	—	8
Moo (2010)^100^	USA	81 (17.3)	—	Retrospective	1. Below normal calcium levels 2.Hypocalcemic symptoms	6	36 (44.4)	5 (6.2)	81 (100)	—	—	—	8
Shen (2010)^99^	USA	382 (28.0)	47.4	Retrospective	1. Calcium<2.0 mmol/l 2. Calcium/vitamin D supplementation	6	—	1 (0.7)	382 (100)	—	—	—	7
Wilhelm (2009)^98^	USA	136 (11.8)	36.4	Retrospective	Calcium/vitamin D supplementation	6	90 (66.2)	—	—	—	—	—	7
Palestini (2008)^102^	Italy	305 (26.6)	47.0	Prospective	Calcium/vitamin D supplementation	6	64 (21.0)	4 (1.3)	—	157 (51.5)	—	139(45.6)	7
Testini (2007)^103^	Italy	1309 (18.4)	50.6	Retrospective	Calcium/vitamin D supplementation	6	51 (3.9)	2 (0.2)	23 (1.8)	—	—	14 (1.1)	8
Alimoglu (2005)^107^	Turkey	100 (14.0)	47.0	Prospective	Calcium supplementation	6	18 (18.0)	2 (2.0)	—	—	—	—	8
Ku (2005)^106^	China	214 (17.3)	—	Retrospective	1.Below normal PTH levels 2. Calcium/vitamin D supplementation	12	26 (12.1)	4 (1.9)	—	—	—	—	7
Ozbas (2005)^105^	Turkey	750 (–)	—	Retrospective	1. Calcium<2.0 mmol/l 2. Calcium/vitamin D supplementation	6	131 (17.5)	1 (0.1)	—	—	—	—	8
Palazzo (2005)^104^	Australia	1196 (–)	—	Retrospective	1.Below normal calcium levels 2. Calcium/vitamin D supplementation	6	—	10 (0.8)	238 (19.9)	—	—	890 (74.4)	7
Thomusch (2000)^109^	Germany	7266 (22.9)	51.8	Prospective	Calcium/vitamin D supplementation	6	464 (6.4)	108 (1.5)	—	—	—	—	7
Zhou (2000)^108^	China	312 (14.7)	44.5	Retrospective	Calcium/vitamin D supplementation	6	—	3 (1.0)	78 (25.0)	—	—	—	8

a HypoPT persisting beyond the definition time frame (6 or 12 months) is classified as permanent hypoPT, otherwise categorized as transient hypoPT.

cN, clinical N; CND, central neck dissection; LND, lateral neck dissection; PA, parathyroid autotransplantation; permanent hypoparathyroidism; Permanent hypoPT; PTH, parathyroid hormone; transient hypoparathyroidism; Transient hypoPT; TT, total thyroidectomy.

### Screening of potential risk factors

**Figure 2:**
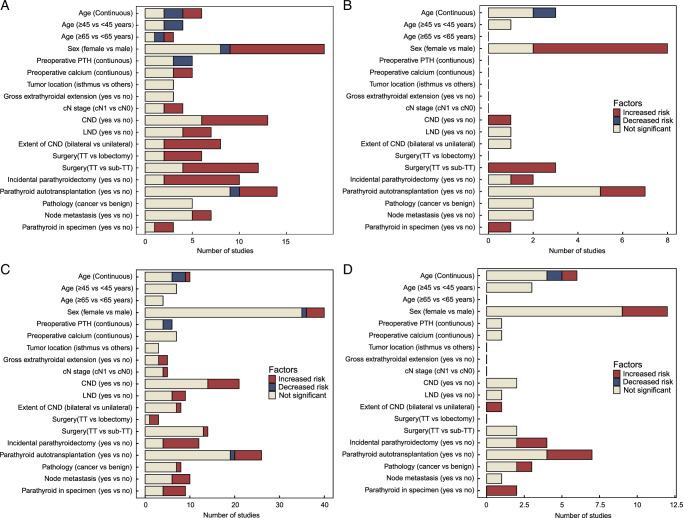
Distribution and results of studies considered for systematic review and meta-analysis. (A) Distribution of results from univariable analysis in included studies for transient hypoPT. (B) Distribution of results from multivariate analysis in included studies for transient hypoPT. (C) Distribution of results from univariable analysis in included studies for permanent hypoPT. (D) Distribution of results from multivariate analysis in included studies for permanent hypoPT. cN, clinical N; CND, central neck dissection; LND, lateral neck dissection; PTH, parathyroid hormone; PGRIS, parathyroid glands remaining in situ; Transient hypoPT, Transient hypoparathyroidism; TT, total thyroidectomy; Permanent hypoPT, Permanent hypoparathyroidism.

We systematically screened potential risk factors in each study according to inclusion criteria, ultimately identifying 19 factors for transient and permanent hypoPT (Table S2A-D, Supplemental Digital Content 4, http://links.lww.com/JS9/C441 and S3A-E, Supplemental Digital Content 4, http://links.lww.com/JS9/C441). Among these, sex and PA were the most frequently reported risk factors for both transient hypoPT and permanent hypoPT. More than half of the studies reported five risk factors were significantly associated with transient hypoPT in both univariate and multivariate analyses, which were sex (female vs. male), CND, surgery [total thyroidectomy (TT) vs. sub-total thyroidectomy (sub-TT)], incidental parathyroidectomy, parathyroid in specimen (Fig. 2A&B). In the case of permanent hypoPT, the number of statistically significant risk factors was notably reduced compared to transient hypoPT, but incidental parathyroidectomy and parathyroid in specimen were still important factors (Fig. 2C, D).

### Meta-analysis of potential risk factors

**Figure 3:**
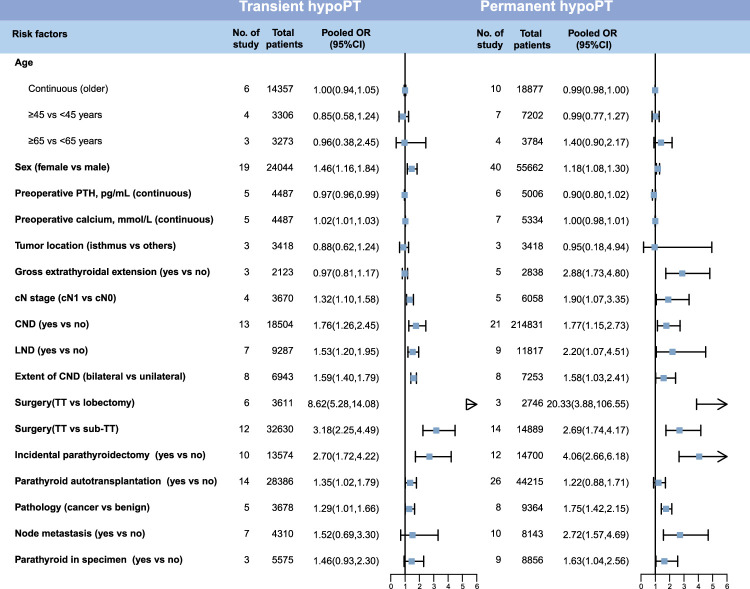
Forest plots showing the meta-analysis results for each potential risk factor. This figure displays the combined results of meta-analysis for both transient and permanent hypoPT, with the specific inclusion details of each study shown in the supplementary figures. CND, central neck dissection; cN, clinical N; hypoPT, hypoparathyroidism; LND, lateral neck dissection; OR, odds ratio; PTH, parathyroid hormone; PGRIS, parathyroid glands remaining in situ; TT, total thyroidectomy.

After meta-analysis of each potential risk factor, we identified 11 statistically significant risk factors associated with transient hypoPT and 10 risk factors related to permanent hypoPT (Fig. 3 and Figure S1A-E, Supplemental Digital Content 5, http://links.lww.com/JS9/C442 and S2A-H, Supplemental Digital Content 5, http://links.lww.com/JS9/C442, Supplemental Digital Content 6, http://links.lww.com/JS9/C443). Two risk factors were found to be associated only with an increased risk of transient hypoPT: preoperative calcium (continuous, OR: 1.02, 95% CI: 1.01–1.03), and PA (OR: 1.35, 95% CI: 1.02–1.79). While preoperative PTH level was a protective factor for transient hypoPT (OR: 0.97, 95% CI: 0.96-0.99). Interestingly, gross extrathyroidal extension (OR: 2.88, 95% CI: 1.73–4.80), node metastasis (OR: 2.72, 95% CI: 1.57–4.69) and parathyroid in specimen (OR: 1.63, 95% CI: 1.04–2.56) were associated only with an increased risk of permanent hypoPT.

Among these variables, nine risk factors were found to be associated with both transient hypoPT and permanent hypoPT. These factors [OR (95%CI)] were sex [female vs. male, transient: 1.46 (1.16–1.84) and permanent: 1.18 (1.08–1.30)], cN stage [transient: 1.32 (1.10–1.58) and permanent: 1.90 (1.07–3.35)], CND [transient: 1.76 (1.26–2.45) and permanent: 1.77 (1.15–2.73)], LND [transient: 1.53 (1.20–1.95) and permanent: 2.20 (1.07,4.51)], extent of CND [bilateral vs. unilateral, transient: 1.59 (1.40–1.79) and permanent: 1.58 (1.03–2.41)], surgery [TT vs. lobectomy, transient: 8.62 (5.28–14.08) and permanent: 20.33 (3.88–106.55)], surgery [TT vs. sub-TT, transient: 3.18 (2.25–4.49) and permanent: 2.69 (1.74–4.17)], incidental parathyroidectomy [transient: 2.70 (1.72–4.22] and permanent: 4.06 (2.66–6.18)], and pathology [cancer vs. benign, transient: 1.29 (1.01–1.66) and permanent: 1.75 (1.42–2.15)].

### Subgroup meta-analysis of potential risk factors

**Figure 4:**
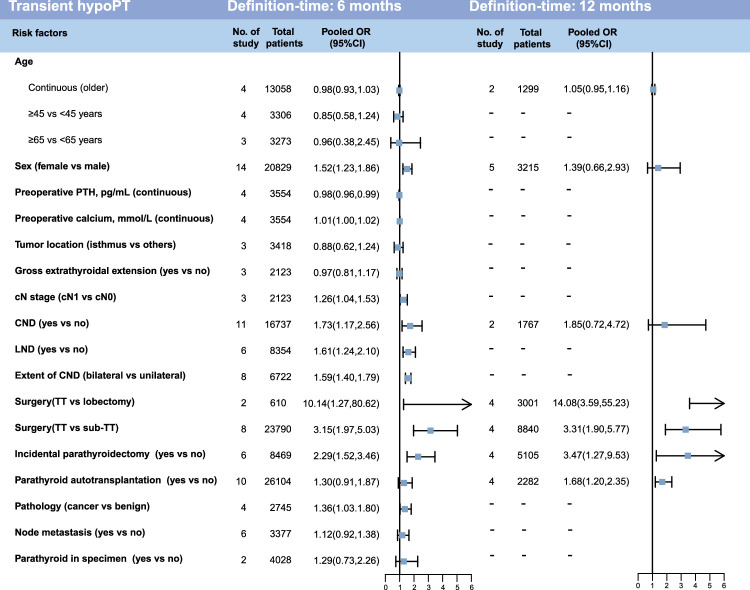
Forest plots showing the subgroup meta-analysis results for each potential risk factor of transient hypoPT. This figure displays the results of subgroup meta-analysis for transient hypoPT with different definition time, and the specific inclusion details of each study were shown in the supplementary figures. cN, clinical N; CND, central neck dissection; hypoPT, hypoparathyroidism; LND, lateral neck dissection; OR, odds ratio; PTH, parathyroid hormone; PGRIS, parathyroid glands remaining in situ; TT, total thyroidectomy.

**Figure 5:**
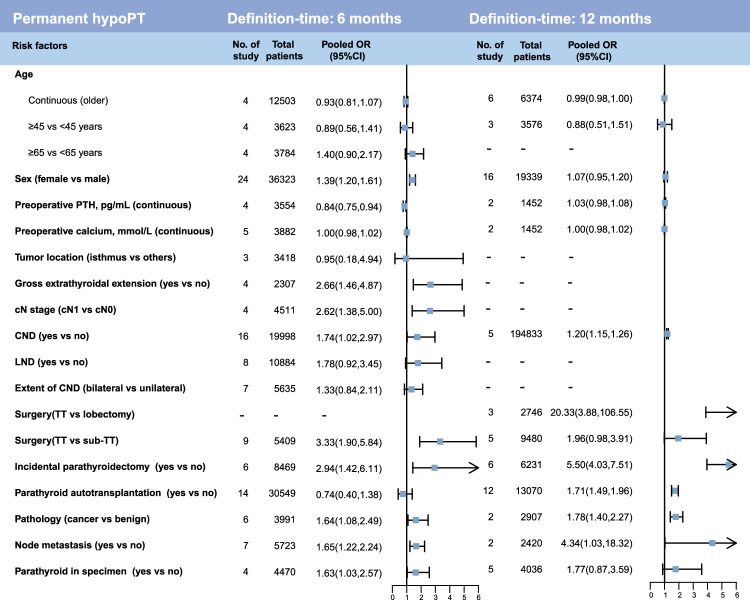
Forest plots showing the subgroup meta-analysis results for each potential risk factor of permanent hypoPT. This figure displays the results of subgroup meta-analysis for permanent hypoPT with different definition time, and the specific inclusion details of each study were shown in the supplementary figures. cN, clinical N; CND, central neck dissection; hypoPT, hypoparathyroidism; LND, lateral neck dissection; OR, odds ratio; PTH, parathyroid hormone; PGRIS, parathyroid glands remaining in situ; TT, total thyroidectomy.

Due to the different time-definitions for transient and permanent hypoPT (12 and 6 months), we conducted subgroup analyses for all risk factors (Figs. 4, 5 and Figure S3-6, Supplemental Digital Content 5, http://links.lww.com/JS9/C442). For transient hypoPT with 6-month definition time, the previous analysis revealed that 10 significant risk factors, including sex (female vs. male), preoperative calcium, cN stage, CND, LND, extent of CND, surgery (TT vs. lobectomy), surgery (TT vs. sub-TT), incidental parathyroidectomy, and pathology (cancer vs. benign), maintained their statistical significance in the subgroup analyses (Fig. 4). For transient hypoPT with 12-month definition-time, only surgery (TT vs. lobectomy), surgery (TT vs. sub-TT), incidental parathyroidectomy and PA showed significant association with the outcomes. Surgery (TT vs. lobectomy), surgery (TT vs. sub-TT) and incidental parathyroidectomy were significant risk factors for transient hypoPT regardless of the timing of its definition.

However, there were some differences in the results of the subgroup analysis for permanent hypoPT compared to the previous analysis (Fig. 5). In the analysis of permanent hypoPT with a 6-month definition time, nine factors, namely sex (female vs. male), gross extrathyroidal extension, cN stage, CND, surgery (TT vs. sub-TT), incidental parathyroidectomy, pathology (cancer vs. benign), node metastasis, parathyroid in specimen. Two risk factors exhibited changes: LND and extent of CND (bilateral vs. unilateral) shifted to a negative result. In both the 6-month and 12-month subgroups, CND, incidental parathyroidectomy, pathology (cancer vs. benign), node metastasis were important risk factors for permanent hypoPT. Interestingly, in the analysis of permanent hypoPT with a 12-month definition time, sex (female vs. male) was no longer associated with permanent hypoPT, while PA emerged as a statistically significant risk factor for 12-month permanent hypoPT.

### Heterogeneity and publication bias

The heterogeneity and publication bias were shown in Table 2. There was publication bias in sex (female vs. male, Egger’s *P*=0.01) and extent of CND (bilateral vs. unilateral, Begg’ *P*=0.02) for transient hypoPT, in age (≥45 vs.<45 years, Egger’s *P*=0.04), preoperative PTH (Egger’s *P*=0.04), node metastasis (Egger’ *P*=0.04) for permanent hypoPT. According to the GRADE quality assessment, the conclusions regarding sex (female vs. male), CND, surgery (TT vs. sub-TT), and incidental parathyroidectomy (yes vs. no) were relatively reliable.

**Table 2:** The heterogeneity and publication bias in the meta-analysis.

	Transient hypoPT					Permanent hypoPT				
			Publication bias					Publication bias		
Risk factor	Statistical method	$I^{2}$ (%)	Begg’s p	Egger’s p	GRADE Quality^a^	Statistical method	$I^{2}$ (%)	Begg’s p	Egger’s p	GRADE Quality^a^
Age - continuous (older)	Random	93	1.00	0.43	Very low	Random	69	0.47	0.28	Low
Age (≥45 vs<45 years)	Random	78	0.31	0.33	Very low	Fixed	11	0.07	0.04	Very low
Age (≥65 vs<65 years)	Random	92	1.00	0.98	Very low	Fixed	49	0.09	0.31	Very low
Sex (female vs male)	Random	90	0.11	0.01	Low	Fixed	29	0.57	0.54	Moderate
Preoperative PTH, pg/ml (continuous)	Fixed	42	0.81	0.28	Very low	Random	57	0.45	0.04	Very low
Preoperative calcium, mmol/L (continuous)	Random	80	0.81	0.62	Very low	Fixed	0	0.55	0.16	Low
Tumor location (isthmus vs others)	Fixed	24	0.30	0.11	Very low	Fixed	0	0.30	0.64	Very low
Gross extrathyroidal extension (yes vs no)	Fixed	0	1.00	0.90	Very low	Fixed	0	0.46	0.16	Very low
cN stage (cN1 vs cN0)	Fixed	35	0.73	0.50	Very low	Fixed	34	1.00	0.84	Very low
CND (yes vs no)	Random	69	0.76	0.96	Moderate	Random	76	0.29	0.19	Moderate
LND (yes vs no)	Random	51	1.00	0.92	Low	Random	67	0.75	0.85	Low
Extent of CND (bilateral vs unilateral)	Fixed	2	0.02	0.16	Very low	Fixed	15	0.71	0.48	Low
Surgery (TT vs lobectomy)	Fixed	39	0.71	0.26	Low	Fixed	0	0.30	0.14	Very low
Surgery (TT vs sub-TT)	Random	77	1.00	0.45	Moderate	Fixed	0	0.91	0.40	Moderate
Incidental parathyroidectomy (yes vs no)	Random	89	0.72	0.79	Moderate	Random	53	0.54	0.27	Moderate
PA (yes vs no)	Random	70	0.44	0.24	Low	Random	57	0.25	0.07	Low
Pathology (cancer vs benign)	Fixed	0	0.09	0.23	Very low	Fixed	8	0.90	0.92	Low
Node metastasis (yes vs no)	Random	81	1.00	0.44	Low	Random	54	0.59	0.04	Very low
Parathyroid in specimen (yes vs no)	Random	75	1.00	0.14	Very low	Random	76	0.92	0.08	Low

High quality: Further research is very unlikely to change our confidence in the estimate of effect.

Moderate quality: Further research is likely to have an important impact on our confidence in the estimate of effect and may change the estimate.

Low quality: Further research is very likely to have an important impact on our confidence in the estimate of effect and is likely to change the estimate.

Very low quality: We are very uncertain about the estimate.

a GRADE Working Group grades of evidence.

cN, clinical N; CND, central neck dissection; hypoPT, hypoparathyroidism; LND, lateral neck dissection; PA: parathyroid autotransplantation; PGRIS, parathyroid glands remaining in situ; PTH, parathyroid hormone; TT, total thyroidectomy.

## Discussion

HypoPT is a common complication following thyroid surgery, however, current research findings on the analysis of risk factors for post-thyroid surgery hypoPT are not entirely consistent^7–11^. Furthermore, the potential differential impact of the same risk factors on transient and permanent hypoPT requires further exploration and analysis. Despite some meta-analyses discussing hypoPT, there is still a lack of a comprehensive study to definitively identify preoperative risk factors for both transient and permanent hypoPT^15–17^. In this study, we reviewed and analyzed 93 studies addressing risk factors for transient and permanent hypoPT, identifying nine common risk factors for both transient and permanent hypoPT, two risk factors specific to transient hypoPT, and three common risk factors for permanent hypoPT.

Studies on hypoPT predominantly relies on the presence of abnormalities in postoperative PTH and serum calcium or the need for oral supplementation of vitamin D and calcium as the definition for hypoPT^8–14,24–109^. However, distinguishing between transient and permanent hypoPT in different studies varies regarding the timeframes, with some studies employing 6-month and others using 12-month^8–14,24–109^. A meta-analysis suggested that the overall incidence of hypoPT did not significantly differ under different time definitions^15^. In our study, we harmonized and performed subgroup analysis for postoperative hypoPT defined at 6 months and 12 months. The definition of time to a certain extent influenced the analysis of risk factors for hypoPT. It altered sex (female vs. male), CND, PA for transient hypoPT, and sex (female vs. male), surgery (TT vs. sub-TT), PA in permanent hypoPT. To reduce the disparities reported in various studies, there is still a need to establish uniform and widely applicable clinical criteria for postoperative hypoPT.

This large-sample meta-analysis revealed that female patients are higher risk to experience transient and permanent hypoPT after thyroid surgery compared to male patients. There are several explanations for the observed findings. Firstly, female patients often present with more severe thyroid conditions preoperatively, such as hyperthyroidism or thyromegaly^110,111^. This complexity in female patients undergoing thyroid surgery increases the likelihood of parathyroid injury compared to males. Secondly, sex differences in hormone and vitamin D levels can affect postoperative parathyroid function recovery. Estrogen, for example, influences parathyroid function and enhances calcium absorption in the intestines, helping to maintain stable blood calcium levels^112^. However, hormonal changes during menopause and emotional responses to disease and surgery may disrupt hormone balance, increasing the risk of postoperative hypoPT^113–115^. Additionally, females often exhibit stronger immune responses, which may affect postoperative inflammation and healing processes, thereby increasing the risk of hypoPT^116^. Clinically, female patients identified as having a higher risk of postoperative hypoPT should be considered for more intensive treatment and disease management.

Although the relationship between CND/LND and the development of permanent hypoPT remains controversial, it is significantly associated with transient hypoPT after surgery^13,117^. CND/LND aims to clear neck lymph nodes infiltrated by cancer cells and prevent cancer cell spread^118–120^. The degree of protection for the parathyroid glands during CND/LND largely depends on the surgeon’s experience and skill^121^. Accidental damage, traction, or burning of the parathyroid glands during surgery can lead to postoperative hypoPT^3,122^. Our analysis suggests that surgical approach and the extent of lymph node dissection were the most significant influencing factors for postoperative hypoPT (both transient and permanent). The primary consideration for the surgical approach and CND/LND should be the management of tumor metastasis, with a focus on minimizing the occurrence of transient hypoPT during surgery.

The primary cause of postoperative hypoPT is the damage or removal of parathyroid glands during thyroid surgery, and markers related to parathyroid status often exhibit a correlation with postoperative hypoPT^3^. Our analysis indicated that incidental parathyroidectomy was a high-risk factor for both transient and permanent hypoPT. PA is a remedial measure for inadvertent parathyroidectomy during thyroid surgery, and patients undergoing PA may experience transient hypoPT postoperatively, as the reestablishment of blood supply and recovery of parathyroid function require some time^123–126^. However, the restoration of PTH through PA is limited and may not fully return patients to the same level as those who did not experience incidental parathyroidectomy. Another challenging aspect to assess was parathyroid ischemia, as it may result in only mild or even normal gland discoloration^1^. Nevertheless, study by Promberger suggests that patients with discolored parathyroid glands may experience temporary functional impairment postoperatively^127^. In summary, it is essential to minimize incidental parathyroidectomy during surgery and assess parathyroid ischemia to reduce the incidence of postoperative hypoPT.

The application of novel technologies holds significant promise in reducing postoperative hypoPT. With advancements in medical technology, techniques such as image-guided surgery and microsurgical methods provide surgeons with clearer visualization and finer precision during operations, facilitating meticulous capsular dissection and the identification and preservation of parathyroid glands^128–130^. Utilizing appropriate intraoperative strategies for parathyroid identification and preservation is particularly beneficial for surgeons with less experience in the field. The use of carbon nanoparticles enhances the dissection of lymph nodes while safeguarding the parathyroid glands and their function^131^. Near-infrared autofluorescence has also been shown to decrease the risk of transient hypoPT, although its impact on persistent hypoPT requires further validation^132^. A meta-analysis revealed that autofluorescence, indocyanine green fluorescence, and carbon nanoparticles offer superior protection of intraoperative parathyroid glands compared to visual inspection alone^133^.

Several limitations should also be acknowledged in this meta-analysis. Firstly, because there is currently no universally accepted standard for defining postoperative hypoPT, different studies have varying definitions of hypoPT, which can indeed reduce the comparability and persuasiveness of research findings. Secondly, the included criteria were limited to English language studies, potentially leading to exclusion of non-English relevant data and publication bias. Thirdly, our conclusions were drawn based on univariate associations between risk factors and hypoPT rather than multivariable analysis that can account for interactive effects and confounding factors, mainly dure to the heterogeneity in data presentation and absence of consistent multivariable variables across studies. For instance, inconsistencies between PA and permanent hypoPT may stem from the inability to correct for incidental parathyroidectomy and surgical experience across different studies. Finally, based on a limited number of studies, we analyzed some potential risk factors. Some of these factors, such as surgeons’ surgical experience and postoperative levels of PTH and Ca, may also be associated with hypoPT. However, due to not meeting inclusion criteria or lacking uniform meta-analysis quantitative indicators, they were not extensively discussed. Therefore, our meta-analysis results should be interpreted with caution.

## Conclusion

In this study, we compiled and analyzed 93 studies on risk factors for postoperative hypoPT in thyroid surgery. To sum up, postoperative hypoPT is common in female thyroid cancer patients with lymph node metastasis undergoing TT combined with neck dissection. There are many common risk factors for transient and permanent hypoPT including sex, cN stage, CND, LND, surgery type, incidental parathyroidectomy, and pathology. These findings have important implications for clinical practitioners in thyroid surgery and parathyroid research. A deeper understanding of these risk factors holds promise for developing more effective treatment strategies and postoperative care plans, with the potential to reduce the occurrence of postoperative hypoPT and enhance patient quality of life and treatment outcomes.

## Ethical approval

Not applicable.

## Consent

Not applicable.

## Sources of funding

This work was supported by the National Natural Science Foundation of China (82072981, 82272649, and 82303881), Guangdong Basic and Applied Basic Research Foundation (No. 2019A1515010150, 2023A1515010450, 2023A1515012903, and 2022A1515110033) and Open Project Fund of the Sixth Affiliated Hospital of Guangzhou Medical University (202011-201).

## Author contribution

K.N., X.Z., Z.J., A.Y., T.W., and Z.Y.: project development; K.N., Y.Y., X.Z., Z.L., X.L., Y.W., X.Y., W.W., J.B., Q.C., and F.C.: data collection or management; K.N., Y.Y., X.Z., Z.L., Y.L., Z.Z., and L.L.: data analysis or interpretation; K.N., Y.Y., M.J., A.Y., T.W., and Z.Y.: manuscript writing/editing.

## Conflicts of interest disclosure

The authors declare that there are no conflicts of interest.

## Research registration unique identifying number (UIN)

The study has been registered with the International Prospective Register of Systematic Reviews (PROSPERO: https://www.crd.york.ac.uk/prospero/) prior to the initiation of data extraction (CRD42023412216).

## Guarantor

Ankui Yang, MD, PhD; Tong Wu, MD, PhD; Zhongyuan Yang, MD, PhD.

## Data availability statement

All relevant data supporting the findings of this study are available within the supplementary materials and/or upon request. The datasets generated and/or analyzed during the current study are available from the corresponding author on reasonable request.

## Provenance and peer review

Not commissioned, externally peer-reviewed.

## Supplementary Material
