# Supplementary figures and images for: Risk factors of transient and permanent hypoparathyroidism after thyroidectomy: a systematic review and meta-analysis
Source: Int J Surg. 2024 Apr 23;110(8):5047–62. doi: 10.1097/JS9.0000000000001475 (PMC11326036; doi:10.1097/JS9.0000000000001475)

## Identification of studies via databases and registers

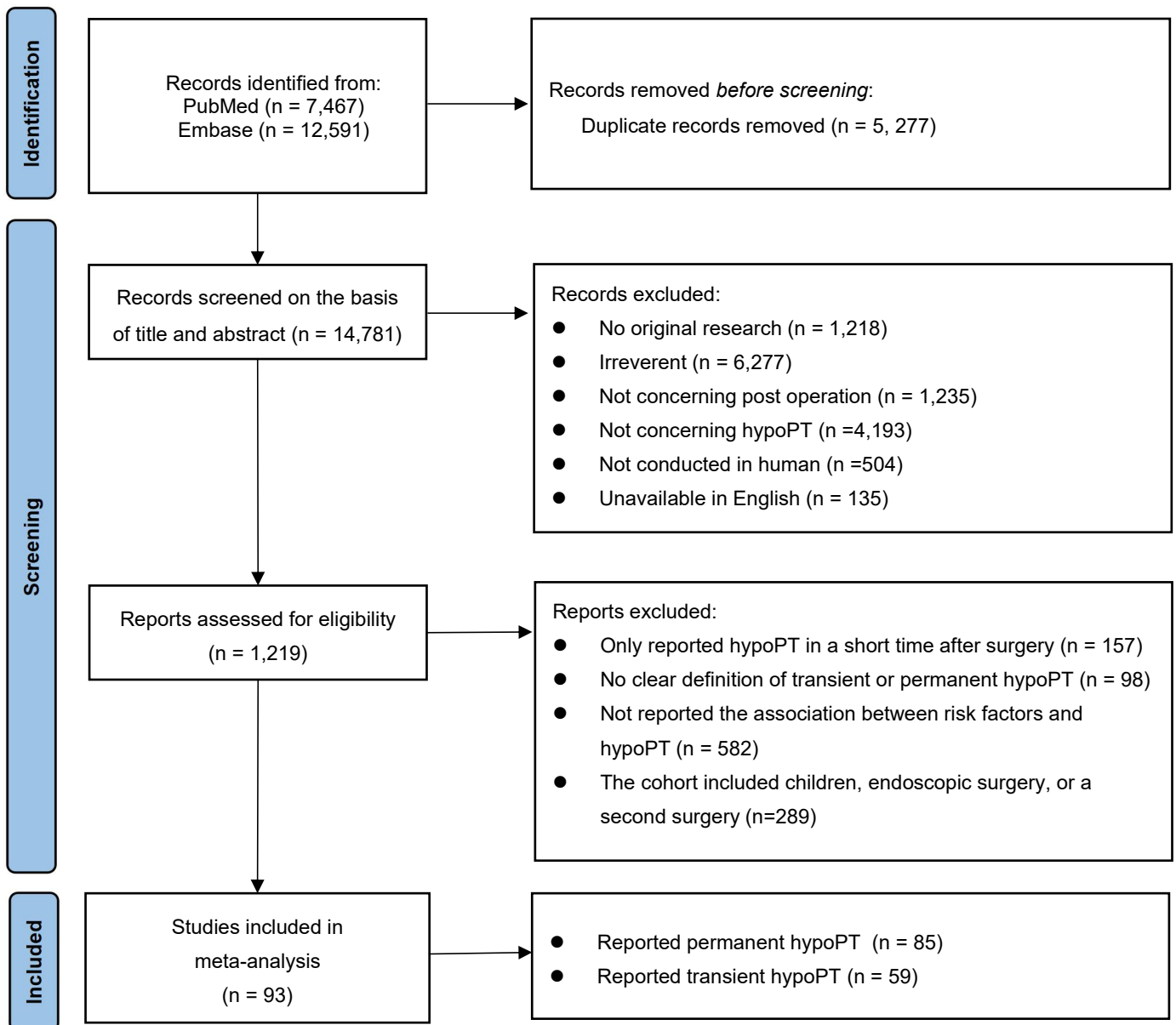

Supplement: Supplementary file 2 [file js9-110-5047-s002.pdf]
