# Supplementary material for: Risk factors of transient and permanent hypoparathyroidism after thyroidectomy: a systematic review and meta-analysis
Source: Int J Surg. 2024 Apr 23;110(8):5047–62. doi: 10.1097/JS9.0000000000001475 (PMC11326036; doi:10.1097/JS9.0000000000001475)
Supplement: Supplementary file 4 [file js9-110-5047-s004.docx]

**Table S1A. The search strategy used in the meta-analysis conducted in PubMed. (From inception to Jun 21, 2024)**

| **PubMed** |  | **Number** |
| --- | --- | --- |
| #1 | "Risk Factors"[MeSH Terms] | 975384 |
| #2 | “Factor, Risk” OR “Risk Factor” OR “Social Risk Factors” OR “Factor, Social Risk” OR “Factors, Social Risk” OR “Risk Factor, Social” OR “Risk Factors, Social” OR “Social Risk Factor” OR “Health Correlates” OR “Correlates, Health” OR “Population at Risk” OR “Populations at Risk” OR “Risk Scores” OR “Risk Score” OR “Score, Risk” OR “Risk Factor Scores” OR “Risk Factor Score” OR “Score, Risk Factor” OR “Prediction” OR “Influencing factors” OR “Association factors” | 2146706 |
| #3 | "Sex"[MeSH Terms] OR "Age Factors"[MeSH Terms] OR "Calcium"[MeSH Terms] OR "Parathyroid Hormone"[MeSH Terms] OR "Lymphatic Metastasis"[MeSH Terms] | 932886 |
| #4 | “Phenotypic Sex” OR “Sex, Phenotypic” OR “Genotypic Sex” OR “Sex, Genotypic” OR “Gender” OR “Male” OR “Males” OR “Female” OR “Females” | 13194384 |
| #5 | “Age Factor” OR “Factor, Age” OR “Factors, Age” OR “Age Reporting” OR “Age” | 3242431 |
| #6 | “Blood Coagulation Factor IV” OR “Coagulation Factor IV” OR “Factor IV, Coagulation” OR “Calcium-40” OR “Calcium 40” OR “Factor IV” | 664999 |
| #7 | “Hormone, Parathyroid” OR “PTH (1-84)” OR “Parathyroid Hormone (1-84)” OR “Parathormone” OR “Parathyrin” OR “Parathyroid Hormone Peptide (1-34)” OR “Parathyroid Hormone (1-34)” OR “PTH (1-34)” OR “Natpara” OR “PTH” OR “Parathyroid hormone” OR “Parathyroid hormones” | 54385 |
| #8 | “Surgery, General” OR “Surgery” OR “Operation” OR “Surgical Procedures” OR “Approach” OR “ Surgical Intervention” OR “Procedure” OR “Operative Treatment” OR “Surgical Operation” OR “Thyroidectomy” OR “Total Thyroidectomy” OR “Completion Total Thyroidectomy” OR “Completion Thyroidectomy” OR “Near-Total Thyroidectomy” OR “Subtotal Thyroidectomy” OR “Unilateral Thyroid Lobectomy” OR “Partial Thyroidectomy” OR “Hemithyroidectomy” OR “Thyroid Nodule Excision” OR “Reoperative Thyroid Surgery” OR “Dunhill Procedure” OR “Thyroid Laparoscopic Surgery” OR “Laparoscopic Thyroidectomy” OR “Minimally Invasive Thyroid Surgery” OR “Endoscopic Thyroid Surgery” OR “Small Incision Thyroid Surgery” OR “Thyroidectomy with Endoscopy” OR “Endoscopic Thyroidectomy” OR “Conventional Open Thyroidectomy” | 6390299 |
| #9 | “Lymph Node Dissection” OR “Lymph Node Surgery” OR “Lymph Nodes Dissected” OR “Central Lymph Node Dissection” OR “CLND” OR “Lateral Lymph Node Dissection” OR “LLND” OR “Central Dissection” OR “Neck Dissection” OR “Central Neck Dissection” OR “CND” OR “Lateral Neck Dissection” OR “LND” OR “Central Compartment Neck Dissection” OR “CCND” OR “Lateral Compartment Node Dissection” OR “LCND” OR “Selective Neck Dissection” OR “Modified Radical Neck Dissection” OR “MRND” OR “Functional Neck Dissection” OR “Radical Neck Dissection” OR “Upper Mediastinal Dissection” | 40219 |
| #10 | “Lymphatic Metastases” OR “Lymph Node Metastasis” OR “Lymph Node Metastases” OR “Metastasis, Lymph Node” OR “Node Metastasis” OR “Metastasis at Diagnosis” OR “Metastatic Lymph Nodes” | 72460 |
| #11 | “Surgical Expertise” OR “Surgeon’s Experience” | 3241 |
| #12 | “Parathyroids autografted” OR “Parathyroids removed” OR “PTG autotransplantation” OR “Parathyroid gland autotransplantation” OR “PG autotransplantation” OR “Parathyroid autotransplantation” OR “Parathyroid glands autotransplanted” OR “Implant of parathyroid gland” OR “Auto-transplantation performed” OR “PGs auto-transplanted” OR “Parathyroid glands auto-transplanted” OR “Parathyroid re-implantation” OR “Parathyroid reimplantation” OR “Autotransplantation” OR “Transplantation” OR “Autotransplanted PGs” | 875589 |
| #13 | “Malignant pathology” OR “Malignant neoplasm” OR “Malignancy” OR “Cancer diagnosis” OR “Thyroid carcinoma” OR “Malignant tumor” OR “Malignant” OR “Papillary Thyroid Carcinoma” OR “FollicularThyroid Carcinoma” OR “Medullary Thyroid Carcinoma” OR “Anaplastic thyroid cancer” OR “Poorly differentiated thyroid carcinoma” OR “Anaplastic thyroid cancer” | 659170 |
| #14 | “Incidental parathyroidectomy” OR “Accidental PG resection” OR “Accidental parathyroid gland resection” OR “Inadvertent parathyroidectomy” OR “Inadvertent parathyroid resection” | 131 |
| #15 | “Parathyroid glands on specimen” OR “PGs on specimen” | 590 |
| #16 | OR #1-#15 | 18631918 |
| #17 | "Thyroid Gland"[MeSH Terms] OR “Thyroid Neoplasms” [MeSH Terms] | 113326 |
| #18 | “Gland, Thyroid” OR “Glands, Thyroid” OR “Thyroid Glands” OR “Thyroid*” OR “Thyroids” | 266757 |
| #19 | “Neoplasm, Thyroid” OR “Thyroid Neoplasm” OR “Neoplasms, Thyroid” OR “Thyroid Carcinoma” OR “Carcinoma, Thyroid” OR “Carcinomas, Thyroid” OR “Thyroid Carcinomas” OR “Cancer of Thyroid” OR “Thyroid Cancers” OR “Thyroid Cancer” OR “Cancer, Thyroid” OR “Cancers, Thyroid” OR “Cancer of the Thyroid” OR “Thyroid Adenoma” OR “Adenoma, Thyroid” OR “Adenomas, Thyroid” OR “Thyroid Adenomas” OR “Papillary Thyroid Carcinoma” OR “Follicular Thyroid Carcinoma” OR “Medullary Thyroid Carcinoma” OR “Anaplastic thyroid cancer” OR “poorly differentiated thyroid carcinoma” OR “Anaplastic thyroid cancer” | 53987 |
| #20 | OR #17-#19 | 266757 |
| #21 | "General Surgery"[MeSH Terms] OR “Postoperative Period” [MeSH Terms] | 102475 |
| #22 | “Surgery, General” OR “Surgery” OR “Operation” OR “Surgical Procedures” OR “Approach” OR “Surgical Intervention” OR “Procedure” OR “Operative Treatment” OR “Surgical Operation” | 6379884 |
| #23 | “Period, Postoperative” OR “Periods, Postoperative” OR “Postoperative Periods” OR “Post-surgery” OR “After surgery” OR “Post-surgery Period” OR “Postoperative” OR “Post-operation” | 1109128 |
| #24 | "Thyroidectomy"[MeSH Terms] | 25695 |
| #25 | “Thyroidectomies” OR “Thyroid removal” OR “Thyroid resection” OR “Postoperative thyroidectomy” OR “Thyroid surgery” OR “Total Thyroidectomy” OR “Completion Total Thyroidectomy” OR “Completion Thyroidectomy” OR “Near-Total Thyroidectomy” OR “Subtotal Thyroidectomy” OR “Unilateral Thyroid Lobectomy” OR “Partial Thyroidectomy” OR “Hemithyroidectomy” OR “Thyroid Nodule Excision” OR “Reoperative Thyroid Surgery” OR “Dunhill Procedure” OR “Thyroid Laparoscopic Surgery” OR “Laparoscopic Thyroidectomy” OR “Minimally Invasive Thyroid Surgery” OR “Endoscopic Thyroid Surgery” OR “Conventional Open Thyroidectomy” | 27878 |
| #26 | OR #21-#25 | 6527535 |
| #27 | #20 AND #26 | 83875 |
| #28 | "Hypoparathyroidism"[MeSH Terms] OR "Vitamin D"[MeSH Terms] OR "Hypocalcemia"[MeSH Terms] OR "Calcium"[MeSH Terms] OR "Parathyroid Hormone"[MeSH Terms] OR “Postoperative Complications"[MeSH Terms] | 980145 |
| #29 | “Idiopathic Hypoparathyroidism” OR “Hypoparathyroidism, Idiopathic” OR “HypoPT” | 10814 |
| #30 | “Hormone, Parathyroid” OR “PTH (1-84)” OR “Parathyroid Hormone (1-84)” OR “Parathormone” OR “Parathyrin” OR “Parathyroid Hormone Peptide (1-34)” OR “Parathyroid Hormone (1-34)” OR “PTH(1-34)” OR “Natpara” OR “PTH” OR “Parathyroid hormone” OR “Parathyroid hormones” | 54385 |
| #31 | “Blood Coagulation Factor IV” OR “Coagulation Factor IV” OR “Factor IV, Coagulation” OR “Calcium-40” OR “Calcium 40” OR “Factor IV” | 664999 |
| #32 | “Hypocalcemias” OR “Hypocalc*” OR “low calcium” | 22300 |
| #33 | “Complication, Postoperative” OR “Complications, Postoperative” OR “Postoperative Complication” OR “Complication” OR “Complications” OR “Associated disease” OR “sequelae” OR “sequels” OR “coexistent disease” OR “concomitant disease” OR “associated conditions” OR “coexistent conditions” OR “concomitant conditions” OR “sequela” OR “Adverse Reaction” OR “Side Effect” OR “Undesirable Effect” OR “Adverse Event” OR “Unintended Consequence” OR “Untoward effect” | 158121 |
| #34 | OR #28-#33 | 697160 |
| #35 | #16 AND #27 AND #34 | 11116 |
| #36 | #35 filters: humans, English | 7467 |

**Table S1B. The search strategy used in the meta-analysis conducted in Embase. (From inception to Jun 21, 2024)**

| **Embase** |  | **Number** |
| --- | --- | --- |
| #1 | ‘Risk Factors’/exp | 1395977 |
| #2 | ‘Factor, Risk’ OR ‘Risk Factor’ OR ‘Social Risk Factors’ OR ‘Factor, Social Risk’ OR ‘Factors, Social Risk’ OR ‘Risk Factor, Social’ OR ‘Risk Factors, Social’ OR ‘Social Risk Factor’ OR ‘Health Correlates’ OR ‘Correlates, Health’ OR ‘Population at Risk’ OR ‘Populations at Risk’ OR ‘Risk Scores’ OR ‘Risk Score’ OR ‘Score, Risk’ OR ‘Risk Factor Scores’ OR ‘Risk Factor Score’ OR ‘Score, Risk Factor’ OR ‘Prediction’ OR ‘Influencing factors’ OR ‘Association factors’ | 2378056 |
| #3 | ‘Sex’/exp OR ‘Age Factors’/exp OR ‘Calcium’/exp OR ‘Parathyroid Hormone’/exp OR ‘Lymphatic Metastasis’/exp | 2071850 |
| #4 | ‘Phenotypic Sex’ OR ‘Sex, Phenotypic’ OR ‘Genotypic Sex’ OR ‘Sex, Genotypic’ OR ‘Gender’ OR ‘Male’ OR ‘Males’ OR ‘Female’ OR ‘Females’ | 16356373 |
| #5 | ‘Age Factor’ OR ‘Factor, Age’ OR ‘Factors, Age’ OR ‘Age Reporting’ OR ‘Age’ | 5144001 |
| #6 | ‘Blood Coagulation Factor IV’ OR ‘Coagulation Factor IV’ OR ‘Factor IV, Coagulation’ OR ‘Calcium-40’ OR ‘Calcium 40’ OR ‘Factor IV’ | 347 |
| #7 | ‘Hormone, Parathyroid’ OR ‘PTH’ OR ‘Parathyroid hormone’ OR ‘Parathyroid hormones’ | 100469 |
| #8 | ‘Surgery, General’ OR ‘Surgery’ OR ‘Operation’ OR ‘Surgical Procedures’ OR ‘Approach’ OR ‘ Surgical Intervention’ OR ‘Procedure’ OR ‘Operative Treatment’ OR ‘Surgical Operation’ OR ‘Thyroidectomy’ OR ‘Total Thyroidectomy’ OR ‘Completion Total Thyroidectomy’ OR ‘Completion Thyroidectomy’ OR ‘Near-Total Thyroidectomy’ OR ‘Subtotal Thyroidectomy’ OR ‘Unilateral Thyroid Lobectomy’ OR ‘Partial Thyroidectomy’ OR ‘Hemithyroidectomy’ OR ‘Thyroid Nodule Excision’ OR ‘Reoperative Thyroid Surgery’ OR ‘Dunhill Procedure’ OR ‘Thyroid Laparoscopic Surgery’ OR ‘Laparoscopic Thyroidectomy’ OR ‘Minimally Invasive Thyroid Surgery’ OR ‘Endoscopic Thyroid Surgery’ OR ‘Small Incision Thyroid Surgery’ OR ‘Thyroidectomy with Endoscopy’ OR ‘Endoscopic Thyroidectomy’ OR ‘Conventional Open Thyroidectomy’ | 9428376 |
| #9 | ‘Lymph Node Dissection’ OR ‘Lymph Node Surgery’ OR ‘Lymph Nodes Dissected’ OR ‘Central Lymph Node Dissection’ OR ‘CLND’ OR ‘Lateral Lymph Node Dissection’ OR ‘LLND’ OR ‘Central Dissection’ OR ‘Neck Dissection’ OR ‘Central Neck Dissection’ OR ‘CND’ OR ‘Lateral Neck Dissection’ OR ‘LND’ OR ‘Central Compartment Neck Dissection’ OR ‘CCND’ OR ‘Lateral Compartment Node Dissection’ OR ‘LCND’ OR ‘Selective Neck Dissection’ OR ‘Modified Radical Neck Dissection’ OR ‘MRND’ OR ‘Functional Neck Dissection’ OR ‘Radical Neck Dissection’ OR ‘Upper Mediastinal Dissection’ | 129370 |
| #10 | ‘Lymphatic Metastases’ OR ‘Lymph Node Metastasis’ OR ‘Lymph Node Metastases’ OR ‘Metastasis, Lymph Node’ OR ‘Node Metastasis’ OR ‘Metastasis at Diagnosis’ OR ‘Metastatic Lymph Nodes’ | 200296 |
| #11 | ‘Surgical Expertise’ | 1561 |
| #12 | ‘Parathyroids autografted’ OR ‘Parathyroids removed’ OR ‘PTG autotransplantation’ OR ‘Parathyroid gland autotransplantation’ OR ‘PG autotransplantation’ OR ‘Parathyroid autotransplantation’ OR ‘Parathyroid glands autotransplanted’ OR ‘Implant of parathyroid gland’ OR ‘Auto-transplantation performed’ OR ‘PGs auto-transplanted’ OR ‘Parathyroid glands auto-transplanted’ OR ‘Parathyroid re-implantation’ OR ‘Parathyroid reimplantation’ OR ‘Autotransplantation’ OR ‘Transplantation’ OR ‘Autotransplanted PGs’ | 1239613 |
| #13 | ‘Malignant pathology’ OR ‘Malignant neoplasm’ OR ‘Malignancy’ OR ‘Cancer diagnosis’ OR ‘Thyroid carcinoma’ OR ‘Malignant tumor’ OR ‘Malignant’ OR ‘Papillary Thyroid Carcinoma’ OR ‘FollicularThyroid Carcinoma’ OR ‘Medullary Thyroid Carcinoma’ OR ‘Anaplastic thyroid cancer’ OR ‘Poorly differentiated thyroid carcinoma’ OR ‘Anaplastic thyroid cancer’ | 1358656 |
| #14 | ‘Incidental parathyroidectomy’ OR ‘Accidental PG resection’ OR ‘Accidental parathyroid gland resection’ OR ‘Inadvertent parathyroidectomy’ OR ‘Inadvertent parathyroid resection’ | 125 |
| #15 | ‘Parathyroid glands on specimen’ OR ‘PGs on specimen’ | 0 |
| #16 | OR #1-#15 | 13259688 |
| #17 | ‘Thyroid Gland’/exp OR ‘Thyroid Neoplasms’/exp | 73534 |
| #18 | ‘Gland, Thyroid’ OR ‘Glands, Thyroid’ OR ‘Thyroid Glands’ OR ‘Thyroid*’ OR ‘Thyroids’ | 386293 |
| #19 | ‘Neoplasm, Thyroid’ OR ‘Thyroid Neoplasm’ OR ‘Neoplasms, Thyroid’ OR ‘Thyroid Carcinoma’ OR ‘Carcinoma, Thyroid’ OR ‘Carcinomas, Thyroid’ OR ‘Thyroid Carcinomas’ OR ‘Cancer of Thyroid’ OR ‘Thyroid Cancers’ OR ‘Thyroid Cancer’ OR ‘Cancer, Thyroid’ OR ‘Cancers, Thyroid’ OR ‘Cancer of the Thyroid’ OR ‘Thyroid Adenoma’ OR ‘Adenoma, Thyroid’ OR ‘Adenomas, Thyroid’ OR ‘Thyroid Adenomas’ OR ‘Papillary Thyroid Carcinoma’ OR ‘Follicular Thyroid Carcinoma’ OR ‘Medullary Thyroid Carcinoma’ OR ‘Anaplastic thyroid cancer’ OR ‘Poorly differentiated thyroid carcinoma’ OR ‘Anaplastic thyroid cancer’ | 94921 |
| #20 | OR #17-#19 | 386458 |
| #21 | ‘General Surgery’/exp OR ‘Postoperative Period’/exp | 22956 |
| #22 | ‘Surgery, General’ OR ‘Surgery’ OR ‘Operation’ OR ‘Surgical Procedures’ OR ‘Approach’ OR ‘Surgical Intervention’ OR ‘Procedure’ OR ‘Operative Treatment’ OR ‘Surgical Operation’ | 9416664 |
| #23 | ‘Period, Postoperative’ OR ‘Periods, Postoperative’ OR ‘Postoperative Periods’ OR ‘Post-surgery’ OR ‘After surgery’ OR ‘Post-surgery Period’ OR ‘Postoperative’ OR ‘Post-operation’ | 1555042 |
| #24 | ‘Thyroidectomy’/exp | 48842 |
| #25 | ‘Thyroidectomies’ OR ‘Thyroid removal’ OR ‘Thyroid resection’ OR ‘Postoperative thyroidectomy’ OR ‘Thyroid surgery’ OR ‘Total Thyroidectomy’ OR ‘Completion Total Thyroidectomy’ OR ‘Completion Thyroidectomy’ OR ‘Near-Total Thyroidectomy’ OR ‘Subtotal Thyroidectomy’ OR ‘Unilateral Thyroid Lobectomy’ OR ‘Partial Thyroidectomy’ OR ‘Hemithyroidectomy’ OR ‘Thyroid Nodule Excision’ OR ‘Reoperative Thyroid Surgery’ OR ‘Dunhill Procedure’ OR ‘Thyroid Laparoscopic Surgery’ OR ‘Laparoscopic Thyroidectomy’ OR ‘Minimally Invasive Thyroid Surgery’ OR ‘Endoscopic Thyroid Surgery’ OR ‘Conventional Open Thyroidectomy’ | 36161 |
| #26 | OR #21-#25 | 6426673 |
| #27 | #20 AND #26 | 66034 |
| #28 | ‘Hypoparathyroidism’/exp OR ‘Vitamin D’/exp OR ‘Hypocalcemia’/exp OR ‘Calcium’/exp OR ‘Parathyroid Hormone’/exp OR ‘Postoperative Complications’/exp | 1426897 |
| #29 | ‘Idiopathic Hypoparathyroidism’ OR ‘Hypoparathyroidism, Idiopathic’ OR ‘HypoPT’ | 1056 |
| #30 | ‘Hormone, Parathyroid’ OR ‘PTH’ OR ‘Parathyroid hormone’ OR ‘Parathyroid hormones’ | 100469 |
| #31 | ‘Blood Coagulation Factor IV’ OR ‘Coagulation Factor IV’ OR ‘Factor IV, Coagulation’ OR ‘Calcium-40’ OR ‘Calcium 40’ OR ‘Factor IV’ | 347 |
| #32 | ‘Hypocalcemias’ OR ‘Hypocalc*’ OR ‘low calcium’ | 43209 |
| #33 | ‘Complication, Postoperative’ OR ‘Complications, Postoperative’ OR ‘Postoperative Complication’ OR ‘Complication’ OR ‘Complications’ OR ‘Associated disease’ OR ‘Adverse Reaction’ OR ‘Side Effect’ OR ‘Undesirable Effect’ OR ‘Adverse Event’ OR ‘Unintended Consequence’ OR ‘Untoward effect’ | 4921022 |
| #34 | OR #28-#33 | 4436313 |
| #35 | #16 AND #27 AND #34 | 14691 |
| #36 | #35 AND [english]/lim | 13920 |
| #37 | #36 AND 'human'/de | 12591 |

**Table S2A. Risk factors examined in** **univariable analyses about transient hypoPT.**

| **Risk factors** | **Eynde**  **(2023)** | **Issa**  **(2023)** | **Ahn**  **(2022)** | **Baud**  **(2022)** | **Xu**  **(2022)** | **Palmhag**  **(2021)** | **Qiu**  **(2021)** | **Di**  **(2020)** | **Jørgensen**  **(2020)** | **Karunakaran**  **(2020)** | **Song**  **(2020)** | **Gambardella**  **(2019)** | **Imga**  **(2019)** | **Jeon**  **(2019)** | **Kong**  **(2019)** | **Kwon**  **(2019)** | **Maurer**  **(2019)** |
| --- | --- | --- | --- | --- | --- | --- | --- | --- | --- | --- | --- | --- | --- | --- | --- | --- | --- |
| Age - continuous (older) |  |  |  |  |  | = |  |  | - |  |  |  | + |  | = | = |  |
| Age (≥ 45 vs < 45 years) |  |  |  |  |  |  |  |  |  |  |  |  |  |  |  |  |  |
| Age (≥ 65 vs < 65 years) | = |  |  |  |  |  |  |  |  |  |  |  |  |  |  |  |  |
| Sex (female vs male) |  |  |  | + |  | = | + |  | = | = |  |  | - |  | = | = |  |
| Preoperative PTH, pg/mL (continuous) |  |  |  |  |  |  | = |  |  |  |  |  | - |  |  |  |  |
| Preoperative calcium, mmol/L (continuous) |  |  |  |  |  |  | = |  |  |  |  |  | + |  |  |  |  |
| Tumor location (isthmus vs others) |  |  |  |  |  |  | = |  |  |  |  |  |  |  |  |  |  |
| Gross extrathyroidal extension (yes vs no) |  |  |  |  |  |  |  |  |  |  |  |  |  |  |  |  |  |
| cN stage (cN1 vs cN0) |  |  |  | + |  |  |  |  |  |  |  |  |  |  |  |  |  |
| CND (yes vs no) |  |  | = |  |  |  | = |  |  |  |  | + |  |  |  |  |  |
| LND (yes vs no) |  |  |  |  |  |  | = |  |  |  |  |  | = |  |  |  |  |
| Extent of CND (bilateral vs unilateral) |  |  |  |  |  |  | + |  |  |  |  |  |  |  |  |  |  |
| Surgery (TT vs lobectomy) |  | = |  |  |  |  |  |  |  |  | + |  |  | + |  |  |  |
| Surgery (TT vs subtotal thyroidectomy) |  |  |  |  |  |  |  | + |  |  |  |  |  |  |  |  | = |
| Incidental parathyroidectomy (yes vs no) |  |  |  |  |  |  | + |  |  |  |  |  |  |  |  |  |  |
| Parathyroid autotransplantation (yes vs no) |  |  |  | + | = | = | = |  | = |  |  |  | = |  |  |  |  |
| Node metastasis (yes vs no) |  |  |  |  |  |  |  |  |  |  |  |  | + |  | = |  |  |
| Pathology (cancer vs benign) |  |  |  |  |  |  |  |  |  |  |  |  | = |  |  | = |  |
| Parathyroid in specimen (yes vs no) |  |  |  | + |  |  |  |  |  |  |  |  |  |  |  |  |  |

**Table S2B. Risk factors examined in univariable analyses about transient hypoPT.**

| **Risk factors** | **Nicholson (2019)** | **Sugino (2019)** | **Su**  **(2018)** | **Thomusch**  **(2018)** | **Vasileiadis (2018)** | **Wang**  **(2018)** | **Yoo**  **(2018)** | **Kwon**  **(2017)** | **Lin**  **(2017)** | **Su**  **(2017)** | **Zheng (2017)** | **He**  **(2016)** | **Jeong**  **(2016)** | **Kim**  **(2016)** | **Lin (2016)** | **Longheu (2016)** | **Park**  **(2016)** |
| --- | --- | --- | --- | --- | --- | --- | --- | --- | --- | --- | --- | --- | --- | --- | --- | --- | --- |
| Age - continuous (older) |  |  |  |  |  |  |  |  |  | = |  |  |  |  |  |  |  |
| Age (≥ 45 vs < 45 years) |  |  | = |  |  |  |  |  |  |  |  |  |  |  |  |  | = |
| Age (≥ 65 vs < 65 years) |  |  |  |  |  |  |  |  |  |  |  |  |  |  |  | - |  |
| Sex (female vs male) |  |  | + |  |  | = |  |  |  | + |  |  | + |  |  |  | + |
| Preoperative PTH, pg/mL (continuous) |  |  | = |  |  | = |  |  |  | - |  |  |  |  |  |  |  |
| Preoperative calcium, mmol/L (continuous) |  |  | = |  |  | = |  |  |  | + |  |  |  |  |  |  |  |
| Tumor location (isthmus vs others) |  |  | = |  |  |  |  |  |  | = |  |  |  |  |  |  |  |
| Gross extrathyroidal extension (yes vs no) |  |  | = |  |  |  |  |  |  | = |  |  |  |  |  |  |  |
| cN stage (cN1 vs cN0) |  |  | = |  |  |  |  |  |  | + |  |  |  |  |  |  |  |
| CND (yes vs no) |  |  |  |  |  | = |  |  |  |  |  |  | = | + |  |  |  |
| LND (yes vs no) |  |  |  |  |  | = |  |  |  |  |  |  | + |  |  |  | + |
| Extent of CND (bilateral vs unilateral) |  |  | + |  |  | + | + |  |  | = |  |  | + |  |  |  | = |
| Surgery (TT vs lobectomy) | = |  |  |  |  |  |  |  |  |  |  |  |  |  |  |  |  |
| Surgery (TT vs subtotal thyroidectomy) |  | + |  | + |  |  |  | + |  |  |  |  |  |  | + |  |  |
| Incidental parathyroidectomy (yes vs no) |  |  | + |  | + |  |  |  | + | + | + |  |  |  |  |  | = |
| Parathyroid autotransplantation (yes vs no) |  |  | + | = |  |  |  |  |  | + |  |  |  |  |  |  | = |
| Node metastasis (yes vs no) |  |  | = |  |  |  |  |  |  |  |  | = |  |  |  |  |  |
| Pathology (cancer vs benign) |  |  |  |  |  | = |  |  |  |  |  |  | = |  |  |  | = |
| Parathyroid in specimen (yes vs no) |  |  |  |  |  |  |  |  |  |  |  |  |  |  |  |  |  |

**Table S2C. Risk factors examined in univariable analyses about transient hypoPT.**

| **Risk factors** | **Selberherr (2016)** | **Carvalho (2015)** | **Daher (2015)** | **Ahn**  **(2014)** | **Calo**  **(2014)** | **Praženica**  **(2014)** | **Puzziello (2014)** | **Song**  **(2014)** | **Wei**  **(2014)** | **Barczyński (2013)** | **Hammerstad (2013)** | **Abboud (2012)** | **Barczyński (2012)** | **Giordano (2012)** | **Barczyński (2011)** | **Wong**  **(2011)** |
| --- | --- | --- | --- | --- | --- | --- | --- | --- | --- | --- | --- | --- | --- | --- | --- | --- |
| Age - continuous (older) |  |  | - |  |  |  |  |  |  |  |  |  |  |  |  |  |
| Age (≥ 45 vs < 45 years) |  |  |  |  |  |  |  |  |  |  |  |  |  |  |  |  |
| Age (≥ 65 vs < 65 years) |  |  |  |  | + |  |  |  |  |  |  |  |  |  |  |  |
| Sex (female vs male) |  |  | + |  |  |  | + | = |  |  | = |  |  |  |  | + |
| Preoperative PTH, pg/mL (continuous) |  |  |  |  |  |  |  |  |  |  |  |  |  |  |  |  |
| Preoperative calcium, mmol/L (continuous) |  |  |  |  |  |  |  |  |  |  |  |  |  |  |  |  |
| Tumor location (isthmus vs others) |  |  |  |  |  |  |  |  |  |  |  |  |  |  |  |  |
| Gross extrathyroidal extension (yes vs no) |  |  |  |  |  |  |  | = |  |  |  |  |  |  |  |  |
| cN stage (cN1 vs cN0) |  |  |  |  |  |  |  | = |  |  |  |  |  |  |  |  |
| CND (yes vs no) |  | + | + | = |  |  |  | + |  | + |  | = |  |  |  |  |
| LND (yes vs no) |  |  | + |  |  |  |  | = |  |  |  |  |  |  |  |  |
| Extent of CND (bilateral vs unilateral) | = |  |  |  |  |  |  | = |  |  |  |  |  | + |  |  |
| Surgery (TT vs lobectomy) |  |  |  |  |  |  |  |  |  |  |  |  |  |  |  |  |
| Surgery (TT vs subtotal thyroidectomy) |  |  |  |  |  |  |  |  |  |  |  |  | = |  | + | + |
| +Incidental parathyroidectomy (yes vs no) |  |  |  |  |  | + |  | + |  |  |  |  |  |  |  |  |
| Parathyroid autotransplantation (yes vs no) |  |  |  |  |  |  |  |  | = |  | = |  |  |  |  | = |
| Node metastasis (yes vs no) |  |  |  |  |  |  |  | = |  |  |  |  |  |  |  |  |
| Pathology (cancer vs benign) |  |  |  |  |  |  |  |  |  |  |  |  |  |  |  |  |
| Parathyroid in specimen (yes vs no) |  |  | = |  |  |  |  | + |  |  |  |  |  |  |  |  |

**Table S2D. Risk factors examined in univariable analyses about transient hypoPT.**

| **Risk factors** | **Barczyński**  **(2010)** | **Moo**  **(2010)** | **Wilhelm**  **(2009)** | **Palestini**  **(2008)** | **Testini**  **(2007)** | **Alimoglu**  **(2005)** | **Ku**  **(2005)** | **Ozbas (2005)** | **Thomusch**  **(2000)** |
| --- | --- | --- | --- | --- | --- | --- | --- | --- | --- |
| Age - continuous (older) |  |  |  |  |  |  |  |  | + |
| Age (≥ 45 vs < 45 years) |  |  |  |  |  |  |  |  |  |
| Age (≥ 65 vs < 65 years) |  |  |  |  |  |  |  |  |  |
| Sex (female vs male) |  |  |  |  |  |  |  |  | + |
| Preoperative PTH, pg/mL (continuous) |  |  |  |  |  |  |  |  |  |
| Preoperative calcium, mmol/L (continuous) |  |  |  |  |  |  |  |  |  |
| Tumor location (isthmus vs others) |  |  |  |  |  |  |  |  |  |
| Gross extrathyroidal extension (yes vs no) |  |  |  |  |  |  |  |  |  |
| cN stage (cN1 vs cN0) |  |  |  |  |  |  |  |  |  |
| CND (yes vs no) |  | + |  | + |  |  |  |  |  |
| LND (yes vs no) |  |  |  |  |  |  |  |  |  |
| Extent of CND (bilateral vs unilateral) |  |  |  |  |  |  |  |  |  |
| Surgery (TT vs lobectomy) |  |  |  |  |  |  |  |  |  |
| Surgery (TT vs subtotal thyroidectomy) | + |  | = |  |  | = | + | + |  |
| Incidental parathyroidectomy (yes vs no) |  |  |  |  |  |  |  |  |  |
| Parathyroid autotransplantation (yes vs no) |  |  |  |  | - |  |  |  |  |
| Node metastasis (yes vs no) |  |  |  | + |  |  |  |  |  |
| Pathology (cancer vs benign) |  |  |  |  |  |  |  |  |  |
| Parathyroid in specimen (yes vs no) |  |  |  |  |  |  |  |  |  |

+: Increased risk of transient hypoPT; –: decreased risk of transient hypoPT; =, non-statistically significant effect. cN: clinical N**;** CND: central neck dissection ; LND: lateral neck dissection; PTH: parathyroid hormone; Transient hypoPT: Transient hypoparathyroidism; TT: total thyroidectomy.

**Table S3A. Risk factors examined in univariable analyses about Permanent hypoPT**

| **Risk factors** | **Annebäck**  **(2024)** | **Eynde**  **(2023)** | **Baud**  **(2022)** | **Lončar**  **(2022)** | **Riordan**  **(2022)** | **Takahashi**  **(2022)** | **Xu**  **(2022)** | **Annebaäck**  **(2021)** | **Lui (2021)** | **Palmhag**  **(2021)** | **Qiu**  **(2021)** | **Salem**  **(2021)** | **Bergenfelz**  **(2020)** | **Di**  **(2020)** | **Godlewska**  **(2020)** | **Jørgensen**  **(2020)** | **Karunakaran**  **(2020)** |
| --- | --- | --- | --- | --- | --- | --- | --- | --- | --- | --- | --- | --- | --- | --- | --- | --- | --- |
| Age - continuous (older) |  |  | - |  |  | = |  |  |  | = |  |  |  |  |  | - |  |
| Age (≥ 45 vs < 45 years) |  |  |  |  |  |  |  |  |  |  |  |  |  |  |  |  |  |
| Age (≥ 65 vs < 65 years) |  | = |  |  | = |  |  |  |  |  |  |  |  |  |  |  |  |
| Sex (female vs male) | = |  | = | = | = | = |  | + | = | = | = | = | = |  | = | = | = |
| Preoperative PTH, pg/mL (continuous) |  |  |  |  |  |  |  |  |  |  | = |  |  |  |  |  |  |
| Preoperative calcium, mmol/L (continuous) |  |  |  |  |  |  |  |  |  |  | = |  |  |  |  |  | = |
| Tumor location (isthmus vs others) |  |  |  |  |  |  |  |  |  |  | = |  |  |  |  |  |  |
| Gross extrathyroidal extension (yes vs no) |  |  |  |  |  |  |  |  |  |  |  |  |  |  |  |  |  |
| cN stage (cN1 vs cN0) |  |  | = |  |  |  |  |  |  |  |  |  |  |  |  |  |  |
| CND (yes vs no) |  |  |  |  |  |  |  |  |  |  | = |  |  |  |  |  |  |
| LND (yes vs no) |  |  |  |  |  |  |  |  |  |  | = |  |  |  |  |  |  |
| Extent of CND (bilateral vs unilateral) |  |  |  |  |  |  |  |  |  |  | = |  |  |  |  |  |  |
| Surgery (TT vs lobectomy) |  |  |  |  |  |  |  |  |  |  |  |  |  | + |  |  |  |
| Surgery (TT vs subtotal thyroidectomy) |  |  |  |  |  |  |  |  |  |  |  |  |  |  |  |  |  |
| Incidental parathyroidectomy (yes vs no) |  |  |  |  |  |  |  |  |  |  | = |  |  |  |  |  |  |
| Parathyroid autotransplantation (yes vs no) | = |  |  | + |  |  | = | + |  | = | = |  |  |  |  | = |  |
| Node metastasis (yes vs no) |  |  |  |  |  |  |  |  |  |  |  |  |  |  |  |  |  |
| Pathology (cancer vs benign) |  |  |  |  | = | + |  |  |  |  |  |  |  |  |  |  |  |
| Parathyroid in specimen (yes vs no) |  |  | + | + | = |  |  |  |  |  |  |  |  |  |  |  |  |

**Table S3B. Risk factors examined in univariable analyses about Permanent hypoPT**

| **Risk factors** | **Song**  **(2020)** | **Wu**  **(2020)** | **Zheng**  **(2020)** | **Díez (2019)** | **Gambardella**  **(2019)** | **Imga (2019)** | **León-Ballesteros**  **(2019)** | **Maurer**  **(2019)** | **Sugino (2019)** | **Falch**  **(2018)** | **Su**  **(2018)** | **Teshima**  **(2018)** | **Thomusch**  **(2018)** | **Vasileiadis (2018)** | **Villarroya-Marquina**  **(2018)** | **Wang**  **(2018)** |
| --- | --- | --- | --- | --- | --- | --- | --- | --- | --- | --- | --- | --- | --- | --- | --- | --- |
| Age - continuous (older) |  |  | = |  |  | = |  |  | = |  |  |  |  |  |  |  |
| Age (≥ 45 vs < 45 years) |  |  |  |  |  |  |  |  |  |  | = |  |  |  |  |  |
| Age (≥ 65 vs < 65 years) |  |  |  |  |  |  |  |  |  |  |  |  |  |  |  |  |
| Sex (female vs male) |  | = | = | = |  | = |  |  | = | = | = | = |  |  | = | = |
| Preoperative PTH, pg/mL (continuous) |  |  |  |  |  | = |  |  |  |  | = |  |  |  |  | - |
| Preoperative calcium, mmol/L (continuous) |  |  |  |  |  | = |  |  |  |  | = |  |  |  |  | + |
| Tumor location (isthmus vs others) |  |  |  |  |  |  |  |  |  |  | = |  |  |  |  |  |
| Gross extrathyroidal extension (yes vs no) |  | + |  |  |  |  |  |  |  |  | = |  |  |  |  |  |
| cN stage (cN1 vs cN0) |  |  |  |  |  |  |  |  |  |  | = | = |  |  |  |  |
| CND (yes vs no) |  | = |  |  | = |  |  |  |  |  |  | = |  |  | = | = |
| LND (yes vs no) |  |  |  |  |  | + |  |  |  |  |  | = |  |  | = | + |
| Extent of CND (bilateral vs unilateral) |  |  |  |  |  |  |  |  |  |  | = |  |  |  |  |  |
| Surgery (TT vs lobectomy) | = |  |  |  |  |  |  |  |  |  |  |  |  |  |  |  |
| Surgery (TT vs subtotal thyroidectomy) |  |  |  |  |  |  |  | = | + |  |  |  |  |  |  |  |
| Incidental parathyroidectomy (yes vs no) |  |  |  |  |  | + | + |  |  |  | + |  |  | + |  |  |
| Parathyroid autotransplantation (yes vs no) |  | = |  |  |  |  | = |  |  | = | = | - | = |  |  |  |
| Node metastasis (yes vs no) |  | = |  |  |  | + | = |  |  |  | = | = |  |  |  |  |
| Pathology (cancer vs benign) |  |  |  |  |  |  |  |  |  | = |  |  |  |  |  | = |
| Parathyroid in specimen (yes vs no) |  | = |  |  |  |  |  |  |  |  |  |  |  |  |  |  |

**Table S3C. Risk factors examined in univariable analyses about Permanent hypoPT**

| **Risk factors** | **Yoo (2018)** | **Kwon (2017)** | **Lin (2017)** | **Lorente-Poch**  **(2017)** | **Serra (2017)** | **Su**  **(2017)** | **Suwannasarn**  **(2017)** | **Zheng (2017)** | **Dubernard**  **(2016)** | **Garrahy**  **(2016)** | **He**  **(2016)** | **Järhult**  **(2016)** | **Jeong**  **(2016)** | **Kim**  **(2017)** | **Lang**  **(2016)** | **Lin (2016)** | **Longheu (2016)** | **Park**  **(2016)** |
| --- | --- | --- | --- | --- | --- | --- | --- | --- | --- | --- | --- | --- | --- | --- | --- | --- | --- | --- |
| Age - continuous (older) |  |  |  |  |  |  |  |  |  |  |  |  |  |  | = |  |  |  |
| Age (≥ 45 vs < 45 years) |  |  |  |  |  | = |  |  |  |  |  |  |  |  |  |  |  | = |
| Age (≥ 65 vs < 65 years) |  |  |  |  |  |  |  |  |  |  |  |  |  |  |  |  | = |  |
| Sex (female vs male) |  |  |  |  |  | = | + |  |  | = |  | = | = |  |  |  |  | = |
| Preoperative PTH, pg/mL (continuous) |  |  |  |  |  | = |  |  |  |  |  |  |  |  |  |  |  |  |
| Preoperative calcium, mmol/L (continuous) |  |  |  |  |  | = |  |  |  |  |  |  |  |  |  |  |  |  |
| Tumor location (isthmus vs others) |  |  |  |  |  | = |  |  |  |  |  |  |  |  |  |  |  |  |
| Gross extrathyroidal extension (yes vs no) |  |  |  |  |  | = |  |  |  |  |  |  |  |  |  |  |  |  |
| cN stage (cN1 vs cN0) |  |  |  |  |  | = |  |  |  |  |  |  |  |  |  |  |  |  |
| CND (yes vs no) |  |  |  |  |  |  | = |  | + | + |  |  | = | + |  |  |  |  |
| LND (yes vs no) |  |  |  |  |  |  |  |  |  |  |  |  | = |  |  |  |  | = |
| Extent of CND (bilateral vs unilateral) | = |  |  |  |  | = |  |  |  |  |  |  | = |  |  |  |  |  |
| Surgery (TT vs lobectomy) |  | + |  |  |  |  |  |  |  |  |  |  |  |  |  |  |  |  |
| Surgery (TT vs subtotal thyroidectomy) |  |  |  |  |  |  | = |  |  |  |  | = |  |  |  | = |  |  |
| Incidental parathyroidectomy (yes vs no) |  |  | + |  | + | + |  | + |  |  |  |  |  |  |  |  |  | = |
| Parathyroid autotransplantation (yes vs no) |  |  |  | + |  | = |  |  |  | = |  |  |  |  | = |  |  | = |
| Node metastasis (yes vs no) |  |  |  |  |  |  |  |  |  |  | = |  |  |  |  |  |  | = |
| Pathology (cancer vs benign) |  |  |  |  |  |  |  |  |  | + |  |  | = |  |  |  |  | = |
| Parathyroid in specimen (yes vs no) |  |  |  |  |  |  |  |  |  | = |  | = |  |  | + |  |  |  |

**Table S3D. Risk factors examined in univariable analyses about Permanent hypoPT**

| **Risk factors** | **Selberherr (2016)** | **Seo (2016)** | **Carvalho (2015)** | **Daher (2015)** | **Lorente-Poch**  **(2015)** | **Wang (2015)** | **Ahn**  **(2014)** | **Almquist**  **(2014)** | **Calo**  **(2014)** | **Nawrot**  **(2014)** | **Praženica**  **(2014)** | **Promberger (2014)** | **Puzziello (2014)** | **Song**  **(2014)** | **Wei**  **(2014)** | **Barczyński (2013)** | **Hammerstad (2013)** |
| --- | --- | --- | --- | --- | --- | --- | --- | --- | --- | --- | --- | --- | --- | --- | --- | --- | --- |
| Age - continuous (older) |  |  |  | - |  |  |  |  |  | = |  | = |  |  |  |  |  |
| Age (≥ 45 vs < 45 years) |  |  |  |  | = |  |  |  |  |  |  |  |  |  |  |  |  |
| Age (≥ 65 vs < 65 years) |  |  |  |  |  |  |  |  | = |  |  |  |  |  |  |  |  |
| Sex (female vs male) |  |  |  | + | = |  |  | = |  | = |  | = | = | = |  |  | = |
| Preoperative PTH, pg/mL (continuous) |  |  |  |  |  |  |  | = |  |  |  |  |  |  |  |  |  |
| Preoperative calcium, mmol/L (continuous) |  |  |  |  |  |  |  | = |  |  |  |  |  |  |  |  |  |
| Tumor location (isthmus vs others) |  |  |  |  |  |  |  |  |  |  |  |  |  |  |  |  |  |
| Gross extrathyroidal extension (yes vs no) |  |  |  |  |  |  |  |  |  |  |  |  |  | = |  |  |  |
| cN stage (cN1 vs cN0) |  |  |  |  |  |  |  |  |  |  |  |  |  | + |  |  |  |
| CND (yes vs no) |  | + | + |  |  | = | + |  |  |  |  |  |  | = |  | = |  |
| LND (yes vs no) |  |  |  | = |  |  |  |  |  |  |  |  |  | + |  |  |  |
| Extent of CND (bilateral vs unilateral) | = |  |  |  |  |  |  |  |  |  |  |  |  | = |  |  |  |
| Surgery (TT vs lobectomy) |  |  |  |  |  |  |  |  |  |  |  |  |  |  |  |  |  |
| Surgery (TT vs subtotal thyroidectomy) |  |  |  |  |  |  |  |  |  |  |  |  |  |  |  |  |  |
| Incidental parathyroidectomy (yes vs no) |  |  |  |  |  |  |  |  |  |  | = |  |  | = |  |  |  |
| Parathyroid autotransplantation (yes vs no) |  |  |  |  | + |  |  |  |  |  |  | + |  |  | - |  | = |
| Node metastasis (yes vs no) |  |  |  |  |  |  |  |  |  |  |  |  |  | + |  |  |  |
| Pathology (cancer vs benign) |  |  |  |  |  |  |  | = |  |  |  |  |  |  |  |  |  |
| Parathyroid in specimen (yes vs no) |  |  |  | + |  |  |  |  |  |  |  |  |  |  |  |  |  |

**Table S3E. Risk factors examined in univariable analyses about Permanent hypoPT**

| **Risk factors** | **Paek**  **(2013)** | **Abboud (2012)** | **Barczyński (2012)** | **Sousa (2012)** | **Barczyński (2011)** | **Wong**  **(2011)** | **Barczyński (2010)** | **Moo**  **(2010)** | **Shen (2010)** | **Palestini (2008)** | **Testini (2007)** | **Alimoglu (2005)** | **Ku (2005)** | **Ozbas (2005)** | **Palazzo**  **(2005)** | **Thomusch**  **(2000)** | **Zhou (2000)** |
| --- | --- | --- | --- | --- | --- | --- | --- | --- | --- | --- | --- | --- | --- | --- | --- | --- | --- |
| Age - continuous (older) |  |  |  |  |  |  |  |  |  |  |  |  |  |  |  | + |  |
| Age (≥ 45 vs < 45 years) | = |  |  |  |  |  |  |  |  |  |  |  |  |  |  |  |  |
| Age (≥ 65 vs < 65 years) |  |  |  |  |  |  |  |  |  |  |  |  |  |  |  |  |  |
| Sex (female vs male) | - |  |  |  |  |  |  |  |  |  |  |  |  |  |  | + |  |
| Preoperative PTH, pg/mL (continuous) |  |  |  |  |  |  |  |  |  |  |  |  |  |  |  |  |  |
| Preoperative calcium, mmol/L (continuous) |  |  |  |  |  |  |  |  |  |  |  |  |  |  |  |  |  |
| Tumor location (isthmus vs others) |  |  |  |  |  |  |  |  |  |  |  |  |  |  |  |  |  |
| Gross extrathyroidal extension (yes vs no) | + |  |  |  |  |  |  |  |  |  |  |  |  |  |  |  |  |
| cN stage (cN1 vs cN0) |  |  |  |  |  |  |  |  |  |  |  |  |  |  |  |  |  |
| CND (yes vs no) |  | = |  |  |  |  |  | = | + | = |  |  |  |  |  |  |  |
| LND (yes vs no) |  |  |  |  |  |  |  |  |  |  |  |  |  |  |  |  |  |
| Extent of CND (bilateral vs unilateral) | + |  |  |  |  |  |  |  |  |  |  |  |  |  |  |  |  |
| Surgery (TT vs lobectomy) |  |  |  |  |  |  |  |  |  |  |  |  |  |  |  |  |  |
| Surgery (TT vs subtotal thyroidectomy) |  |  | = | = | = | = | = |  |  |  |  | = | = | = |  |  | = |
| Incidental parathyroidectomy (yes vs no) |  |  |  |  |  |  |  |  |  |  |  |  |  |  |  |  |  |
| Parathyroid autotransplantation (yes vs no) | = |  |  |  |  | = |  |  |  |  | = |  |  |  | + |  |  |
| Node metastasis (yes vs no) | + |  |  |  |  |  |  |  |  | = |  |  |  |  |  |  |  |
| Pathology (cancer vs benign) |  |  |  |  |  |  |  |  |  |  |  |  |  |  |  |  |  |
| Parathyroid in specimen (yes vs no) | + |  |  |  |  |  |  |  |  |  |  |  |  |  |  |  |  |

+: Increased risk of transient hypoPT; –: decreased risk of transient hypoPT; =, non-statistically significant effect. cN: clinical N**;** CND: central neck dissection ; LND: lateral neck dissection; PTH: parathyroid hormone;; TT: total thyroidectomy; Permanent hypoPT; Permanent hypoparathyroidism.

**Table S4A. The quality of included cohort studies assessed by the Newcastle-Ottawa Quality Assessment Scale.**

| **Category** | **Item** | **Option** | **Annebäck**  **(2024)** | **Eynde**  **(2023)** | **Issa**  **(2023)** | **Ahn**  **(2022)** | **Baud**  **(2022)** | **Lončar**  **(2022)** | **Riordan**  **(2022)** | **Takahashi**  **(2022)** | **Xu**  **(2022)** | **Annebaäck**  **(2021)** |
| --- | --- | --- | --- | --- | --- | --- | --- | --- | --- | --- | --- | --- |
| Selection | Representativeness of the exposed cohort | truly representative of the PTH average after thyroid surgery in the community * | N | N | N | N | N | N | N | N | N | N |
|  |  | somewhat representative of the PTH average after thyroid surgery in the community* | Y* | Y* | Y* | Y* | Y* | Y* | Y* | Y* | N | Y* |
|  |  | selected group of users eg nurses, volunteers | N | N | N | N | N | N | N | N | N | N |
|  |  | no description of the derivation of the cohort | N | N | N | N | N | N | N | N | N | N |
|  | Selection of the non exposed cohort | drawn from the same community as the exposed cohort* | Y* | Y* | Y* | Y* | Y* | Y* | Y* | Y* | Y* | Y* |
|  |  | drawn from a different source | N | N | N | N | N | N | N | N | N | N |
|  |  | no description of the derivation of the non exposed cohort | N | N | N | N | N | N | N | N | N | N |
|  | Ascertainment of exposure | secure record (e.g., surgical records)* | Y* | Y* | Y* | Y* | Y* | Y* | Y* | Y* | Y* | Y* |
|  |  | structured interview* | N | N | N | N | N | N | N | N | N | N |
|  |  | written self report | N | N | N | N | N | N | N | N | N | N |
|  |  | no description | N | N | N | N | N | N | N | N | N | N |
|  | Demonstration that outcome of interest was not present at start of study | yes* | Y* | Y* | Y* | Y* | Y* | Y* | Y* | Y* | Y* | N |
|  |  | no | N | N | N | N | N | N | N | N | N | Y |
| Comparability | Comparability of cohorts on the basis of the design or analysis | study controls for the most important factor* | Y* | Y* | Y* | Y* | Y* | N | Y* | Y* | Y* | Y* |
|  |  | study controls for any additional factor (This criteria could be modified to indicate specific control for a second important factor.) * | Y* | N | N | N | Y* | N | N | Y* | N | Y* |
| Outcome | Assessment of outcome | independent blind assessment * | N | N | N | N | N | N | N | N | N | N |
|  |  | record linkage * | Y* | Y* | Y* | Y* | Y* | Y* | Y* | Y* | Y* | Y* |
|  |  | self report | N | N | N | N | N | N | N | N | N | N |
|  |  | no description | N | N | N | N | N | N | N | N | N | N |
|  | Was follow-up long enough for outcomes to occur (NOT IN CROSS SECTIONAL) | yes, it is an adequate follow up period for outcome of interest* | N | Y* | N | Y* | N | N | N | N | Y* | N |
|  |  | no | N | N | N | N | N | N | N | N | N | N |
|  | Adequacy of follow up of cohorts (NOT IN CROSS SECTIONAL) | complete follow up - all subjects accounted for * | N | N | N | Y* | N | N | N | N | Y* | N |
|  |  | subjects lost to follow up unlikely to introduce bias - small number lost - >80 % (select an adequate %) follow up, or description provided of those lost) * | N | N | N | N | N | N | N | N | N | N |
|  |  | follow up rate < 80% (select an adequate %) and no description of those lost | N | N | N | N | N | N | N | N | N | N |
|  |  | no statement | N | N | N | N | N | N | N | N | N | N |
| Quality Score | | | 8 | 8 | 7 | 9 | 8 | 7 | 7 | 8 | 7 | 7 |

**Table S4B. The quality of included cohort studies assessed by the Newcastle-Ottawa Quality Assessment Scale.**

| **Category** | **Item** | **Option** | **Lui (2021)** | **Palmhag**  **(2021)** | **Qiu**  **(2021)** | **Salem**  **(2021)** | **Bergenfelz**  **(2020)** | **Di**  **(2020)** | **Godlewska**  **(2020)** | **Jørgensen**  **(2020)** | **Karunakaran**  **(2020)** | **Song**  **(2020)** |
| --- | --- | --- | --- | --- | --- | --- | --- | --- | --- | --- | --- | --- |
| Selection | Representativeness of the exposed cohort | truly representative of the PTH average after thyroid surgery in the community * | N | N | N | N | N | N | N | N | N | N |
|  |  | somewhat representative of the PTH average after thyroid surgery in the community* | Y* | Y* | Y* | Y* | Y* | Y* | Y* | Y* | Y* | Y* |
|  |  | selected group of users eg nurses, volunteers | N | N | N | N | N | N | N | N | N | N |
|  |  | no description of the derivation of the cohort | N | N | N | N | N | N | N | N | N | N |
|  | Selection of the non exposed cohort | drawn from the same community as the exposed cohort* | Y* | Y* | Y* | Y* | Y* | Y* | Y* | Y* | Y* | Y* |
|  |  | drawn from a different source | N | N | N | N | N | N | N | N | N | N |
|  |  | no description of the derivation of the non exposed cohort | N | N | N | N | N | N | N | N | N | N |
|  | Ascertainment of exposure | secure record (e.g., surgical records)* | Y* | Y* | Y* | Y* | Y* | Y* | Y* | Y* | Y* | Y* |
|  |  | structured interview* | N | N | N | N | N | N | N | N | N | N |
|  |  | written self report | N | N | N | N | N | N | N | N | N | N |
|  |  | no description | N | N | N | N | N | N | N | N | N | N |
|  | Demonstration that outcome of interest was not present at start of study | yes* | Y* | N | Y* | Y* | Y* | Y* | Y* | Y* | N | N |
|  |  | no | N | Y | N | N | N | N | N | N | Y | Y |
| Comparability | Comparability of cohorts on the basis of the design or analysis | study controls for the most important factor* | Y* | Y* | Y* | Y* | Y* | Y* | Y* | Y* | Y* | Y* |
|  |  | study controls for any additional factor (This criteria could be modified to indicate specific control for a second important factor.) * | N | N | N | Y* | N | N | N | N | N | N |
| Outcome | Assessment of outcome | independent blind assessment * | N | N | N | N | N | N | N | N | N | N |
|  |  | record linkage * | Y* | Y* | Y* | Y* | Y* | Y* | Y* | Y* | Y* | Y* |
|  |  | self report | N | N | N | N | N | N | N | N | N | N |
|  |  | no description | N | N | N | N | N | N | N | N | N | N |
|  | Was follow-up long enough for outcomes to occur (NOT IN CROSS SECTIONAL) | yes, it is an adequate follow up period for outcome of interest* | N | Y* | N | N | N | N | N | Y* | N | Y* |
|  |  | no | N | N | N | N | N | N | N | N | N | N |
|  | Adequacy of follow up of cohorts (NOT IN CROSS SECTIONAL) | complete follow up - all subjects accounted for * | N | Y* | N | N | N | N | N | N | N | Y* |
|  |  | subjects lost to follow up unlikely to introduce bias - small number lost - >80 % (select an adequate %) follow up, or description provided of those lost) * | N | N | N | N | N | Y* | N | N | N | N |
|  |  | follow up rate < 80% (select an adequate %) and no description of those lost | N | N | N | N | N | N | N | N | N | N |
|  |  | no statement | N | N | N | N | N | N | N | N | N | N |
| Quality Score | | | 7 | 8 | 7 | 8 | 7 | 8 | 7 | 8 | 7 | 8 |

**Table S4C. The quality of included cohort studies assessed by the Newcastle-Ottawa Quality Assessment Scale.**

| **Category** | **Item** | **Option** | **Wu**  **(2020)** | **Zheng**  **(2020)** | **Díez (2019)** | **Gambardella**  **(2019)** | **Imga (2019)** | **Jeon (2019)** | **Kong (2019)** | **Kwon**  **(2019)** | **León-Ballesteros**  **(2019)** | **Maurer**  **(2019)** |
| --- | --- | --- | --- | --- | --- | --- | --- | --- | --- | --- | --- | --- |
| Selection | Representativeness of the exposed cohort | truly representative of the PTH average after thyroid surgery in the community * | N | N | N | N | N | N | N | N | N | N |
|  |  | somewhat representative of the PTH average after thyroid surgery in the community* | N | Y* | Y* | Y* | Y* | Y* | Y* | Y* | Y* | Y* |
|  |  | selected group of users eg nurses, volunteers | N | N | N | N | N | N | N | N | N | N |
|  |  | no description of the derivation of the cohort | N | N | N | N | N | N | N | N | N | N |
|  | Selection of the non exposed cohort | drawn from the same community as the exposed cohort* | Y* | Y* | Y* | Y* | Y* | Y* | Y* | Y* | Y* | Y* |
|  |  | drawn from a different source | N | N | N | N | N | N | N | N | N | N |
|  |  | no description of the derivation of the non exposed cohort | N | N | N | N | N | N | N | N | N | N |
|  | Ascertainment of exposure | secure record (e.g., surgical records)* | Y* | Y* | Y* | Y* | Y* | Y* | Y* | Y* | Y* | Y* |
|  |  | structured interview* | N | N | N | N | N | N | N | N | N | N |
|  |  | written self report | N | N | N | N | N | N | N | N | N | N |
|  |  | no description | N | N | N | N | N | N | N | N | N | N |
|  | Demonstration that outcome of interest was not present at start of study | yes* | N | Y* | Y* | Y* | Y* | N | Y* | N | Y* | Y* |
|  |  | no | Y | N | N | N | N | Y | N | Y | N | N |
| Comparability | Comparability of cohorts on the basis of the design or analysis | study controls for the most important factor* | Y* | Y* | Y* | Y* | Y* | Y* | Y* | Y* | Y* | Y* |
|  |  | study controls for any additional factor (This criteria could be modified to indicate specific control for a second important factor.) * | N | N | N | N | N | N | N | N | N | N |
| Outcome | Assessment of outcome | independent blind assessment * | N | N | N | N | N | N | N | N | N | N |
|  |  | record linkage * | N | Y* | Y* | Y* | Y* | Y* | Y* | Y* | Y* | Y* |
|  |  | self report | N | N | N | N | N | N | N | N | N | N |
|  |  | no description | N | N | N | N | N | N | N | N | N | N |
|  | Was follow-up long enough for outcomes to occur (NOT IN CROSS SECTIONAL) | yes, it is an adequate follow up period for outcome of interest* | N | Y* | N | N | N | N | N | Y* | Y* | N |
|  |  | no | N | N | N | N | N | N | N | N | N | N |
|  | Adequacy of follow up of cohorts (NOT IN CROSS SECTIONAL) | complete follow up - all subjects accounted for * | N | Y* | N | N | N | N | N | Y* | N | N |
|  |  | subjects lost to follow up unlikely to introduce bias - small number lost - >80 % (select an adequate %) follow up, or description provided of those lost) * | N | Y* | Y* | Y* | N | N | Y* | Y* | N | Y* |
|  |  | follow up rate < 80% (select an adequate %) and no description of those lost | N | N | N | N | N | N | N | N | N | N |
|  |  | no statement | N | N | N | N | N | N | N | N | N | N |
| Quality Score | | | 6 | 9 | 8 | 8 | 7 | 7 | 8 | 9 | 8 | 8 |

**Table S4D. The quality of included cohort studies assessed by the Newcastle-Ottawa Quality Assessment Scale.**

| **Category** | **Item** | **Option** | **Nicholson (2019)** | **Sugino (2019)** | **Falch**  **(2018)** | **Su**  **(2018)** | **Teshima**  **(2018)** | **Thomusch**  **(2018)** | **Vasileiadis (2018)** | **Villarroya-Marquina**  **(2018)** | **Wang**  **(2018)** |
| --- | --- | --- | --- | --- | --- | --- | --- | --- | --- | --- | --- |
| Selection | Representativeness of the exposed cohort | truly representative of the PTH average after thyroid surgery in the community * | N | N | N | N | N | N | N | N | N |
|  |  | somewhat representative of the PTH average after thyroid surgery in the community* | Y* | Y* | Y* | Y* | Y* | Y* | Y* | Y* | Y* |
|  |  | selected group of users eg nurses, volunteers | N | N | N | N | N | N | N | N | N |
|  |  | no description of the derivation of the cohort | N | N | N | N | N | N | N | N | N |
|  | Selection of the non exposed cohort | drawn from the same community as the exposed cohort* | Y* | Y* | Y* | Y* | Y* | Y* | Y* | Y* | Y* |
|  |  | drawn from a different source | N | N | N | N | N | N | N | N | N |
|  |  | no description of the derivation of the non exposed cohort | N | N | N | N | N | N | N | N | N |
|  | Ascertainment of exposure | secure record (e.g., surgical records)* | Y* | Y* | Y* | Y* | Y* | Y* | Y* | Y* | Y* |
|  |  | structured interview* | N | N | N | N | N | N | N | N | N |
|  |  | written self report | N | N | N | N | N | N | N | N | N |
|  |  | no description | N | N | N | N | N | N | N | N | N |
|  | Demonstration that outcome of interest was not present at start of study | yes* | N | Y* | Y* | Y* | Y* | Y* | N | Y* | Y* |
|  |  | no | Y | N | N | N | N | N | Y | N | N |
| Comparability | Comparability of cohorts on the basis of the design or analysis | study controls for the most important factor* | Y* | Y* | Y* | Y* | Y* | Y* | Y* | Y* | Y* |
|  |  | study controls for any additional factor (This criteria could be modified to indicate specific control for a second important factor.) * | N | N | N | N | N | N | N | N | N |
| Outcome | Assessment of outcome | independent blind assessment * | N | N | N | N | N | N | N | N | N |
|  |  | record linkage * | Y* | Y* | Y* | Y* | Y* | Y* | Y* | Y* | Y* |
|  |  | self report | N | N | N | N | N | N | N | N | N |
|  |  | no description | N | N | N | N | N | N | N | N | N |
|  | Was follow-up long enough for outcomes to occur (NOT IN CROSS SECTIONAL) | yes, it is an adequate follow up period for outcome of interest* | N | Y* | N | Y* | N | N | Y* | Y* | N |
|  |  | no | N | N | N | N | N | N | N | N | N |
|  | Adequacy of follow up of cohorts (NOT IN CROSS SECTIONAL) | complete follow up - all subjects accounted for * | N | Y* | N | Y* | N | N | N | Y* | Y* |
|  |  | subjects lost to follow up unlikely to introduce bias - small number lost - >80 % (select an adequate %) follow up, or description provided of those lost) * | N | Y* | N | N | N | N | Y* | N | N |
|  |  | follow up rate < 80% (select an adequate %) and no description of those lost | N | N | N | N | N | N | N | N | N |
|  |  | no statement | N | N | N | N | N | N | N | N | N |
| Quality Score | | | 7 | 9 | 7 | 9 | 7 | 7 | 8 | 8 | 8 |

**Table S4E. The quality of included cohort studies assessed by the Newcastle-Ottawa Quality Assessment Scale.**

| **Category** | **Item** | **Option** | **Yoo (2018)** | **Kwon (2017)** | **Lin (2017)** | **Lorente-Poch**  **(2017)** | **Serra (2017)** | **Su**  **(2017)** | **Suwannasarn**  **(2017)** | **Zheng (2017)** | **Dubernard**  **(2016)** |
| --- | --- | --- | --- | --- | --- | --- | --- | --- | --- | --- | --- |
| Selection | Representativeness of the exposed cohort | truly representative of the PTH average after thyroid surgery in the community * | N | N | N | N | N | N | N | N | N |
|  |  | somewhat representative of the PTH average after thyroid surgery in the community* | Y* | Y* | Y* | Y* | Y* | Y* | Y* | Y* | Y* |
|  |  | selected group of users eg nurses, volunteers | N | N | N | N | N | N | N | N | N |
|  |  | no description of the derivation of the cohort | N | N | N | N | N | N | N | N | N |
|  | Selection of the non exposed cohort | drawn from the same community as the exposed cohort* | Y* | Y* | Y* | Y* | Y* | Y* | Y* | Y* | Y* |
|  |  | drawn from a different source | N | N | N | N | N | N | N | N | N |
|  |  | no description of the derivation of the non exposed cohort | N | N | N | N | N | N | N | N | N |
|  | Ascertainment of exposure | secure record (e.g., surgical records)* | Y* | Y* | Y* | Y* | Y* | Y* | Y* | Y* | Y* |
|  |  | structured interview* | N | N | N | N | N | N | N | N | N |
|  |  | written self report | N | N | N | N | N | N | N | N | N |
|  |  | no description | N | N | N | N | N | N | N | N | N |
|  | Demonstration that outcome of interest was not present at start of study | yes* | Y* | N | Y* | N | N | Y* | Y* | N | Y* |
|  |  | no | N | Y | N | Y | Y | N | N | Y | N |
| Comparability | Comparability of cohorts on the basis of the design or analysis | study controls for the most important factor* | Y* | Y* | Y* | Y* | Y* | Y* | Y* | Y* | Y* |
|  |  | study controls for any additional factor (This criteria could be modified to indicate specific control for a second important factor.) * | N | N | N | N | N | N | N | N | N |
| Outcome | Assessment of outcome | independent blind assessment * | N | N | N | N | N | N | N | N | N |
|  |  | record linkage * | Y* | Y* | Y* | Y* | Y* | Y* | Y* | Y* | Y* |
|  |  | self report | N | N | N | N | N | N | N | N | N |
|  |  | no description | N | N | N | N | N | N | N | N | N |
|  | Was follow-up long enough for outcomes to occur (NOT IN CROSS SECTIONAL) | yes, it is an adequate follow up period for outcome of interest* | N | N | Y* | Y* | N | N | N | Y* | Y* |
|  |  | no | N | N | N | N | N | N | N | N | N |
|  | Adequacy of follow up of cohorts (NOT IN CROSS SECTIONAL) | complete follow up - all subjects accounted for * | Y* | N | Y* | N | N | N | N | N | Y* |
|  |  | subjects lost to follow up unlikely to introduce bias - small number lost - >80 % (select an adequate %) follow up, or description provided of those lost) * | N | N | Y* | Y* | N | N | Y* | Y* | N |
|  |  | follow up rate < 80% (select an adequate %) and no description of those lost | N | N | N | N | N | N | N | N | N |
|  |  | no statement | N | N | N | N | N | N | N | N | N |
| Quality Score | | | 8 | 7 | 9 | 8 | 7 | 7 | 8 | 8 | 8 |

**Table S4F. The quality of included cohort studies assessed by the Newcastle-Ottawa Quality Assessment Scale.**

| **Category** | **Item** | **Option** | **Garrahy**  **(2016)** | **He (2016)** | **Järhult**  **(2016)** | **Jeong**  **(2016)** | **Kim**  **(2016)** | **Lang**  **(2016)** | **Lin (2016)** | **Longheu (2016)** | **Park**  **(2016)** | **Selberherr (2016)** |
| --- | --- | --- | --- | --- | --- | --- | --- | --- | --- | --- | --- | --- |
| Selection | Representativeness of the exposed cohort | truly representative of the PTH average after thyroid surgery in the community * | N | N | N | N | N | N | N | N | N | N |
|  |  | somewhat representative of the PTH average after thyroid surgery in the community* | Y* | Y* | Y* | Y* | Y* | Y* | Y* | Y* | Y* | Y* |
|  |  | selected group of users eg nurses, volunteers | N | N | N | N | N | N | N | N | N | N |
|  |  | no description of the derivation of the cohort | N | N | N | N | N | N | N | N | N | N |
|  | Selection of the non exposed cohort | drawn from the same community as the exposed cohort* | Y* | Y* | Y* | Y* | Y* | Y* | Y* | Y* | Y* | Y* |
|  |  | drawn from a different source | N | N | N | N | N | N | N | N | N | N |
|  |  | no description of the derivation of the non exposed cohort | N | N | N | N | N | N | N | N | N | N |
|  | Ascertainment of exposure | secure record (e.g., surgical records)* | Y* | Y* | Y* | Y* | Y* | Y* | Y* | Y* | Y* | Y* |
|  |  | structured interview* | N | N | N | N | N | N | N | N | N | N |
|  |  | written self report | N | N | N | N | N | N | N | N | N | N |
|  |  | no description | N | N | N | N | N | N | N | N | N | N |
|  | Demonstration that outcome of interest was not present at start of study | yes* | Y* | Y* | Y* | Y* | Y* | Y* | Y* | Y* | Y* | Y* |
|  |  | no | N | N | N | N | N | N | N | N | N | N |
| Comparability | Comparability of cohorts on the basis of the design or analysis | study controls for the most important factor* | Y* | Y* | Y* | Y* | Y* | Y* | Y* | Y* | Y* | Y* |
|  |  | study controls for any additional factor (This criteria could be modified to indicate specific control for a second important factor.) * | N | N | N | N | N | N | N | N | N | N |
| Outcome | Assessment of outcome | independent blind assessment * | N | N | N | N | N | N | N | N | N | N |
|  |  | record linkage * | Y* | Y* | Y* | Y* | Y* | Y* | Y* | Y* | Y* | Y* |
|  |  | self report | N | N | N | N | N | N | N | N | N | N |
|  |  | no description | N | N | N | N | N | N | N | N | N | N |
|  | Was follow-up long enough for outcomes to occur (NOT IN CROSS SECTIONAL) | yes, it is an adequate follow up period for outcome of interest* | N | N | Y* | N | N | N | N | N | N | Y* |
|  |  | no | N | N | N | N | N | N | N | N | N | N |
|  | Adequacy of follow up of cohorts (NOT IN CROSS SECTIONAL) | complete follow up - all subjects accounted for * | Y* | N | N | N | Y* | N | N | N | N | N |
|  |  | subjects lost to follow up unlikely to introduce bias - small number lost - >80 % (select an adequate %) follow up, or description provided of those lost) * | N | N | N | N | N | Y* | N | N | N | N |
|  |  | follow up rate < 80% (select an adequate %) and no description of those lost | N | N | N | N | N | N | N | N | N | N |
|  |  | no statement | N | N | N | N | N | N | N | N | N | N |
| Quality Score | | | 8 | 7 | 9 | 7 | 8 | 8 | 7 | 7 | 7 | 9 |

**Table S4G. The quality of included cohort studies assessed by the Newcastle-Ottawa Quality Assessment Scale.**

| **Category** | **Item** | **Option** | **Seo (2016)** | **Carvalho (2015)** | **Daher (2015)** | **Lorente-Poch**  **(2015)** | **Wang (2015)** | **Ahn**  **(2014)** | **Almquist**  **(2014)** | **Calo**  **(2014)** | **Nawrot**  **(2014)** | **Praženica**  **(2014)** |
| --- | --- | --- | --- | --- | --- | --- | --- | --- | --- | --- | --- | --- |
| Selection | Representativeness of the exposed cohort | truly representative of the PTH average after thyroid surgery in the community * | N | N | N | N | N | N | N | N | N | N |
|  |  | somewhat representative of the PTH average after thyroid surgery in the community* | Y* | Y* | Y* | Y* | Y* | Y* | Y* | Y* | Y* | N |
|  |  | selected group of users eg nurses, volunteers | N | N | N | N | N | N | N | N | N | N |
|  |  | no description of the derivation of the cohort | N | N | N | N | N | N | N | N | N | N |
|  | Selection of the non exposed cohort | drawn from the same community as the exposed cohort* | Y* | Y* | Y* | Y* | Y* | Y* | Y* | Y* | Y* | Y* |
|  |  | drawn from a different source | N | N | N | N | N | N | N | N | N | N |
|  |  | no description of the derivation of the non exposed cohort | N | N | N | N | N | N | N | N | N | N |
|  | Ascertainment of exposure | secure record (e.g., surgical records)* | Y* | Y* | Y* | Y* | Y* | Y* | Y* | Y* | Y* | Y* |
|  |  | structured interview* | N | N | N | N | N | N | N | N | N | N |
|  |  | written self report | N | N | N | N | N | N | N | N | N | N |
|  |  | no description | N | N | N | N | N | N | N | N | N | N |
|  | Demonstration that outcome of interest was not present at start of study | yes* | Y* | Y* | Y* | Y* | Y* | Y* | Y* | Y* | Y* | N |
|  |  | no | N | N | N | N | N | N | N | N | N | Y |
| Comparability | Comparability of cohorts on the basis of the design or analysis | study controls for the most important factor* | Y* | Y* | Y* | Y* | Y* | Y* | Y* | Y* | Y* | Y* |
|  |  | study controls for any additional factor (This criteria could be modified to indicate specific control for a second important factor.) * | N | N | N | N | N | N | N | N | N | N |
| Outcome | Assessment of outcome | independent blind assessment * | N | N | N | N | N | N | N | N | N | N |
|  |  | record linkage * | Y* | Y* | Y* | Y* | Y* | Y* | Y* | Y* | Y* | N |
|  |  | self report | N | N | N | N | N | N | N | N | N | N |
|  |  | no description | N | N | N | N | N | N | N | N | N | N |
|  | Was follow-up long enough for outcomes to occur (NOT IN CROSS SECTIONAL) | yes, it is an adequate follow up period for outcome of interest* | N | N | N | Y* | N | N | N | N | Y* | N |
|  |  | no | N | N | N | N | N | N | N | N | N | N |
|  | Adequacy of follow up of cohorts (NOT IN CROSS SECTIONAL) | complete follow up - all subjects accounted for * | N | Y* | Y* | Y* | Y* | N | N | N | Y* | N |
|  |  | subjects lost to follow up unlikely to introduce bias - small number lost - >80 % (select an adequate %) follow up, or description provided of those lost) * | N | N | N | N | N | N | N | N | Y* | N |
|  |  | follow up rate < 80% (select an adequate %) and no description of those lost | N | N | N | N | N | N | N | N | N | N |
|  |  | no statement | N | N | N | N | N | N | N | N | N | N |
| Quality Score | | | 7 | 8 | 8 | 9 | 8 | 7 | 7 | 7 | 9 | 6 |

**Table S4H. The quality of included cohort studies assessed by the Newcastle-Ottawa Quality Assessment Scale.**

| **Category** | **Item** | **Option** | **Promberger (2014)** | **Puzziello (2014)** | **Song**  **(2014)** | **Wei**  **(2014)** | **Barczyński (2013)** | **Hammerstad (2013)** | **Paek**  **(2013)** | **Abboud (2012)** | **Barczyński (2012)** |
| --- | --- | --- | --- | --- | --- | --- | --- | --- | --- | --- | --- |
| Selection | Representativeness of the exposed cohort | truly representative of the PTH average after thyroid surgery in the community * | N | N | N | N | N | N | N | N | N |
|  |  | somewhat representative of the PTH average after thyroid surgery in the community* | Y* | Y* | Y* | Y* | Y* | Y* | Y* | Y* | Y* |
|  |  | selected group of users eg nurses, volunteers | N | N | N | N | N | N | N | N | N |
|  |  | no description of the derivation of the cohort | N | N | N | N | N | N | N | N | N |
|  | Selection of the non exposed cohort | drawn from the same community as the exposed cohort* | Y* | Y* | Y* | Y* | Y* | Y* | Y* | Y* | Y* |
|  |  | drawn from a different source | N | N | N | N | N | N | N | N | N |
|  |  | no description of the derivation of the non exposed cohort | N | N | N | N | N | N | N | N | N |
|  | Ascertainment of exposure | secure record (e.g., surgical records)* | Y* | Y* | Y* | Y* | Y* | Y* | Y* | Y* | Y* |
|  |  | structured interview* | N | N | N | N | N | N | N | N | N |
|  |  | written self report | N | N | N | N | N | N | N | N | N |
|  |  | no description | N | N | N | N | N | N | N | N | N |
|  | Demonstration that outcome of interest was not present at start of study | yes* | Y* | Y* | Y* | Y* | Y* | Y* | Y* | Y* | Y* |
|  |  | no | N | N | N | N | N | N | N | N | N |
| Comparability | Comparability of cohorts on the basis of the design or analysis | study controls for the most important factor* | Y* | Y* | Y* | Y* | Y* | Y* | Y* | Y* | Y* |
|  |  | study controls for any additional factor (This criteria could be modified to indicate specific control for a second important factor.) * | N | N | N | N | N | N | N | N | N |
| Outcome | Assessment of outcome | independent blind assessment * | N | N | N | N | N | N | N | N | N |
|  |  | record linkage * | Y* | Y* | Y* | Y* | Y* | Y* | Y* | Y* | Y* |
|  |  | self report | N | N | N | N | N | N | N | N | N |
|  |  | no description | N | N | N | N | N | N | N | N | N |
|  | Was follow-up long enough for outcomes to occur (NOT IN CROSS SECTIONAL) | yes, it is an adequate follow up period for outcome of interest* | N | N | N | N | Y* | N | Y* | N | N |
|  |  | no | N | N | N | N | N | N | N | N | N |
|  | Adequacy of follow up of cohorts (NOT IN CROSS SECTIONAL) | complete follow up - all subjects accounted for * | Y* | Y* | Y* | N | Y* | Y* | Y* | Y* | Y* |
|  |  | subjects lost to follow up unlikely to introduce bias - small number lost - >80 % (select an adequate %) follow up, or description provided of those lost) * | N | N | Y* | N | N | Y* | N | Y* | Y* |
|  |  | follow up rate < 80% (select an adequate %) and no description of those lost | N | N | N | N | N | N | N | N | N |
|  |  | no statement | N | N | N | N | N | N | N | N | N |
| Quality Score | | | 8 | 8 | 8 | 7 | 9 | 8 | 9 | 8 | 8 |

**Table S4I. The quality of included cohort studies assessed by the Newcastle-Ottawa Quality Assessment Scale.**

| **Category** | **Item** | **Option** | **Giordano (2012)** | **Sousa (2012)** | **Barczyński (2011)** | **Wong**  **(2011)** | **Barczyński (2010)** | **Moo**  **(2010)** | **Shen (2010)** | **Wilhelm (2009)** |
| --- | --- | --- | --- | --- | --- | --- | --- | --- | --- | --- |
| Selection | Representativeness of the exposed cohort | truly representative of the PTH average after thyroid surgery in the community * | N | N | N | N | N | N | N | N |
|  |  | somewhat representative of the PTH average after thyroid surgery in the community* | Y* | Y* | Y* | Y* | Y* | Y* | Y* | Y* |
|  |  | selected group of users eg nurses, volunteers | N | N | N | N | N | N | N | N |
|  |  | no description of the derivation of the cohort | N | N | N | N | N | N | N | N |
|  | Selection of the non exposed cohort | drawn from the same community as the exposed cohort* | Y* | Y* | Y* | Y* | Y* | Y* | Y* | Y* |
|  |  | drawn from a different source | N | N | N | N | N | N | N | N |
|  |  | no description of the derivation of the non exposed cohort | N | N | N | N | N | N | N | N |
|  | Ascertainment of exposure | secure record (e.g., surgical records)* | Y* | Y* | Y* | Y* | Y* | Y* | Y* | Y* |
|  |  | structured interview* | N | N | N | N | N | N | N | N |
|  |  | written self report | N | N | N | N | N | N | N | N |
|  |  | no description | N | N | N | N | N | N | N | N |
|  | Demonstration that outcome of interest was not present at start of study | yes* | Y* | Y* | Y* | Y* | Y* | Y* | Y* | Y* |
|  |  | no | N | N | N | N | N | N | N | N |
| Comparability | Comparability of cohorts on the basis of the design or analysis | study controls for the most important factor* | Y* | Y* | Y* | Y* | Y* | Y* | Y* | Y* |
|  |  | study controls for any additional factor (This criteria could be modified to indicate specific control for a second important factor.) * | N | N | N | N | N | N | N | N |
| Outcome | Assessment of outcome | independent blind assessment * | N | N | N | N | N | N | N | N |
|  |  | record linkage * | Y* | Y* | Y* | Y* | Y* | Y* | Y* | Y* |
|  |  | self report | N | N | N | N | N | N | N | N |
|  |  | no description | N | N | N | N | N | N | N | N |
|  | Was follow-up long enough for outcomes to occur (NOT IN CROSS SECTIONAL) | yes, it is an adequate follow up period for outcome of interest* | N | N | N | Y* | N | N | N | N |
|  |  | no | N | N | N | N | N | N | N | N |
|  | Adequacy of follow up of cohorts (NOT IN CROSS SECTIONAL) | complete follow up - all subjects accounted for * | N | Y* | Y* | Y* | Y* | Y* | N | N |
|  |  | subjects lost to follow up unlikely to introduce bias - small number lost - >80 % (select an adequate %) follow up, or description provided of those lost) * | N | Y* | Y* | Y* | Y* | Y* | N | N |
|  |  | follow up rate < 80% (select an adequate %) and no description of those lost | N | N | N | N | N | N | N | N |
|  |  | no statement | N | N | N | N | N | N | N | N |
| Quality Score | | | 7 | 8 | 8 | 9 | 8 | 8 | 7 | 7 |

**Table S4J. The quality of included cohort studies assessed by the Newcastle-Ottawa Quality Assessment Scale.**

| **Category** | **Item** | **Option** | **Palestini (2008)** | **Testini (2007)** | **Alimoglu (2005)** | **Ku (2005)** | **Ozbas (2005)** | **Palazzo**  **(2005)** | **Thomusch**  **(2000)** | **Zhou (2000)** |
| --- | --- | --- | --- | --- | --- | --- | --- | --- | --- | --- |
| Selection | Representativeness of the exposed cohort | truly representative of the PTH average after thyroid surgery in the community * | N | N | N | N | N | N | N | N |
|  |  | somewhat representative of the PTH average after thyroid surgery in the community* | Y* | Y* | Y* | Y* | Y* | Y* | Y* | Y* |
|  |  | selected group of users eg nurses, volunteers | N | N | N | N | N | N | N | N |
|  |  | no description of the derivation of the cohort | N | N | N | N | N | N | N | N |
|  | Selection of the non exposed cohort | drawn from the same community as the exposed cohort* | Y* | Y* | Y* | Y* | Y* | Y* | Y* | Y* |
|  |  | drawn from a different source | N | N | N | N | N | N | N | N |
|  |  | no description of the derivation of the non exposed cohort | N | N | N | N | N | N | N | N |
|  | Ascertainment of exposure | secure record (e.g., surgical records)* | Y* | Y* | Y* | Y* | Y* | Y* | Y* | Y* |
|  |  | structured interview* | N | N | N | N | N | N | N | N |
|  |  | written self report | N | N | N | N | N | N | N | N |
|  |  | no description | N | N | N | N | N | N | N | N |
|  | Demonstration that outcome of interest was not present at start of study | yes* | Y* | Y* | Y* | Y* | Y* | Y* | Y* | Y* |
|  |  | no | N | N | N | N | N | N | N | N |
| Comparability | Comparability of cohorts on the basis of the design or analysis | study controls for the most important factor* | Y* | Y* | Y* | Y* | Y* | Y* | N | Y* |
|  |  | study controls for any additional factor (This criteria could be modified to indicate specific control for a second important factor.) * | N | N | N | N | N | N | N | N |
| Outcome | Assessment of outcome | independent blind assessment * | N | N | N | N | N | N | N | N |
|  |  | record linkage * | Y* | Y* | Y* | Y* | Y* | Y* | Y* | Y* |
|  |  | self report | N | N | N | N | N | N | N | N |
|  |  | no description | N | N | N | N | N | N | N | N |
|  | Was follow-up long enough for outcomes to occur (NOT IN CROSS SECTIONAL) | yes, it is an adequate follow up period for outcome of interest* | N | N | N | N | N | N | N | N |
|  |  | no | N | N | N | N | N | N | N | N |
|  | Adequacy of follow up of cohorts (NOT IN CROSS SECTIONAL) | complete follow up - all subjects accounted for * | N | Y* | Y* | N | Y* | N | Y* | Y* |
|  |  | subjects lost to follow up unlikely to introduce bias - small number lost - >80 % (select an adequate %) follow up, or description provided of those lost) * | N | Y* | Y* | N | Y* | N | N | Y* |
|  |  | follow up rate < 80% (select an adequate %) and no description of those lost | N | N | N | N | N | N | N | N |
|  |  | no statement | N | N | N | N | N | N | N | N |
| Quality Score | | | 7 | 8 | 8 | 7 | 8 | 7 | 7 | 8 |
