# Supplementary material for: Risk factors of transient and permanent hypoparathyroidism after thyroidectomy: a systematic review and meta-analysis
Source: Int J Surg. 2024 Apr 23;110(8):5047–62. doi: 10.1097/JS9.0000000000001475 (PMC11326036; doi:10.1097/JS9.0000000000001475)
Supplement: Supplementary file 5 [file js9-110-5047-s005.pdf]

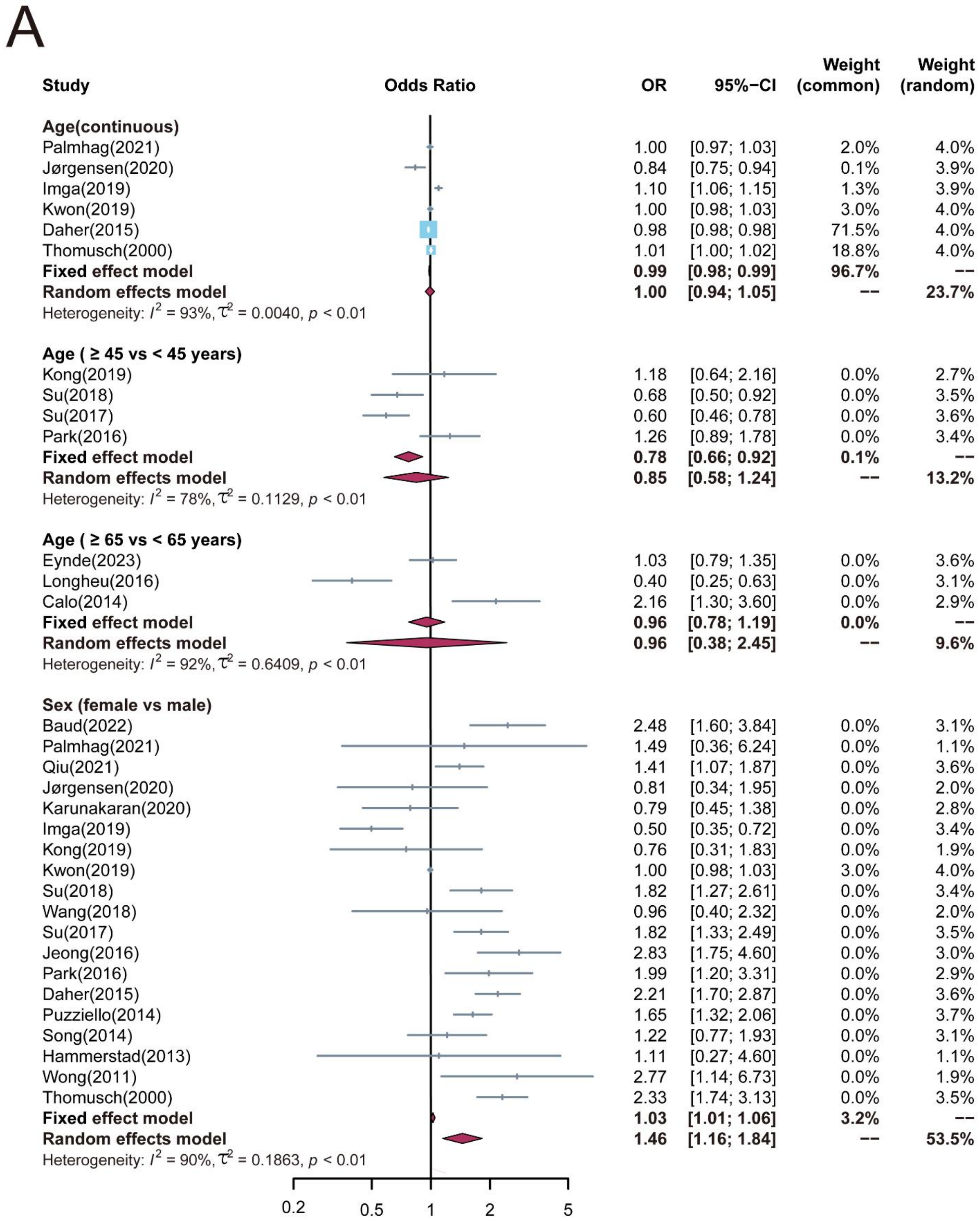

Figure S1A. Forest plots depicting the meta-analysis results of risk factors identified through univariable analyses for transient hypoPT included age(continuous), age (≥ 45 vs < 45 years), age (≥ 65 vs < 65 years), and sex (female vs male).

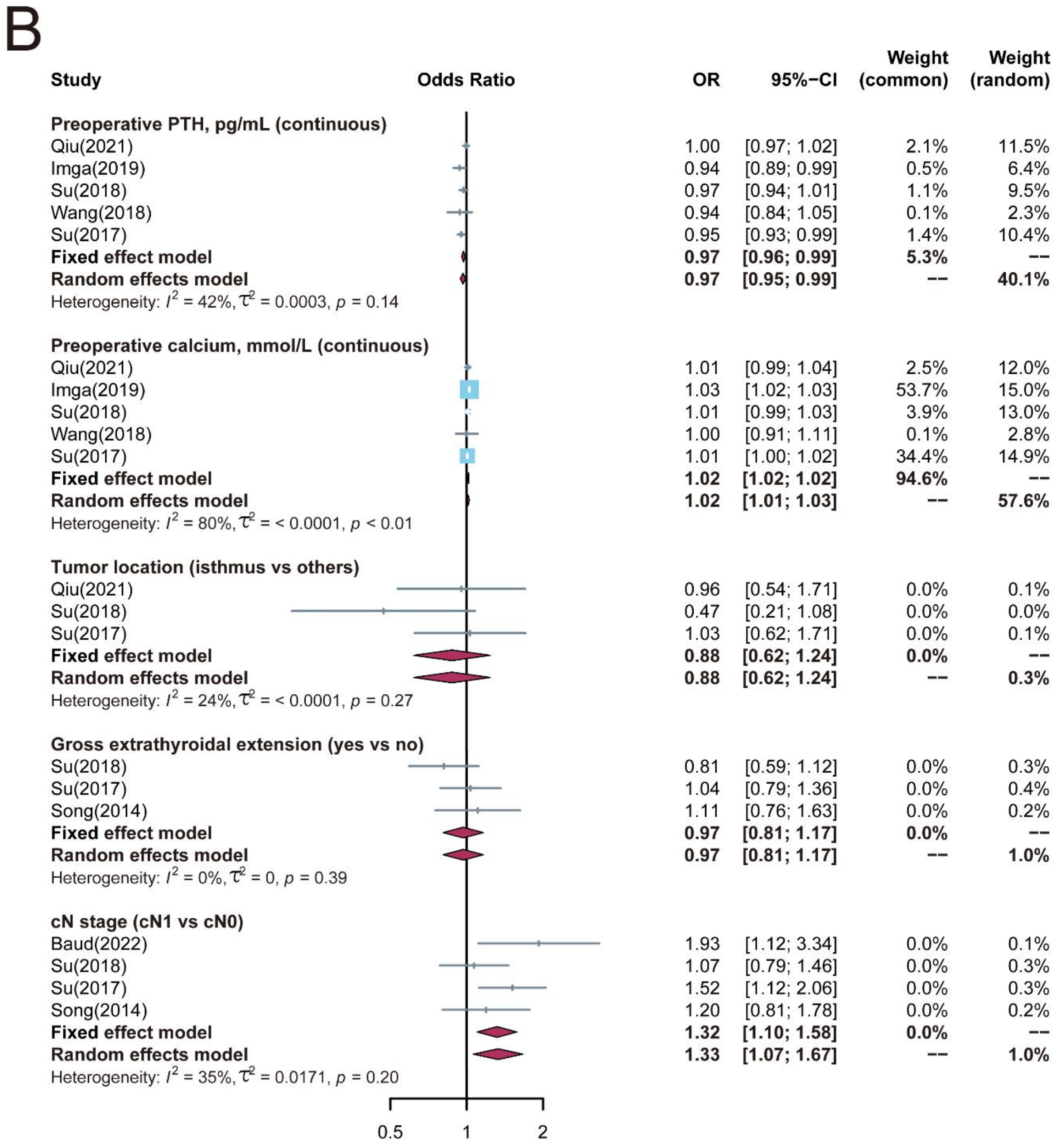

Figure S1B. Forest plots depicting the meta-analysis results of risk factors identified through univariable analyses for transient hypoPT included preoperative PTH, preoperative calcium, tumor location (isthmus vs others), gross extrathyroidal extension, and cN stage (cN1 vs cN0).

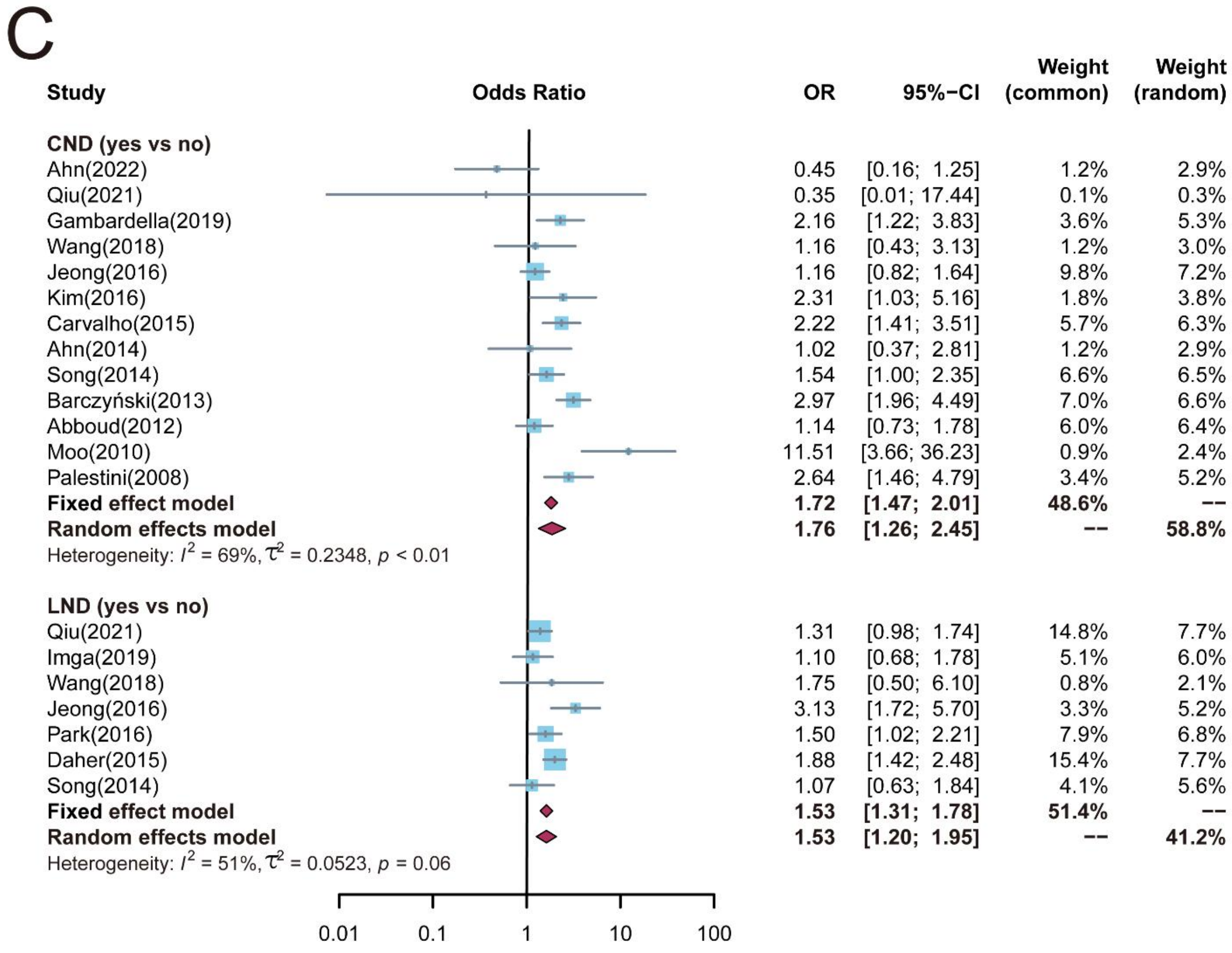

Figure S1C. Forest plots depicting the meta-analysis results of risk factors identified through univariable analyses for transient hypoPT included CND and LND.

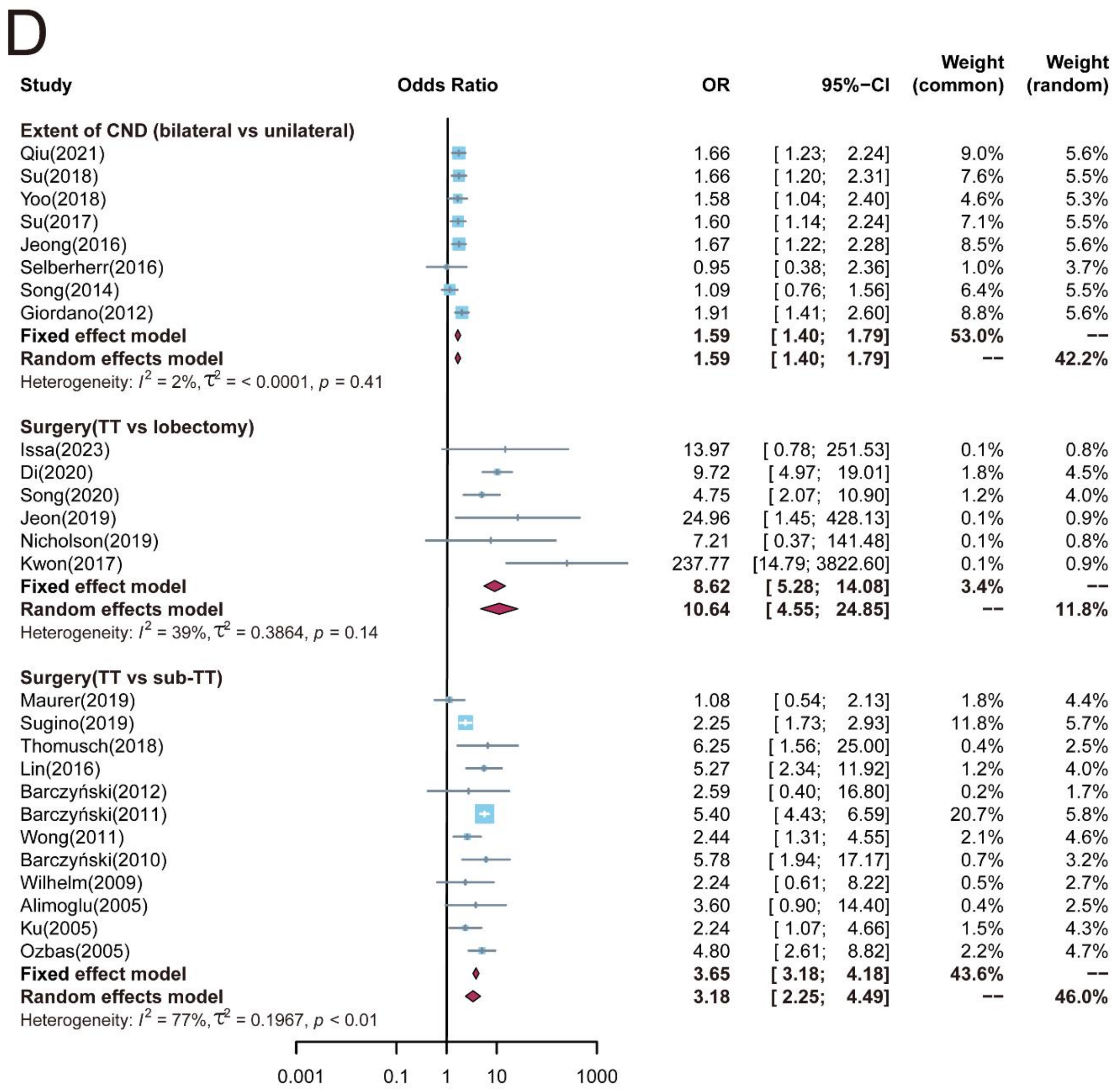

Figure S1D. Forest plots depicting the meta-analysis results of risk factors identified through univariable analyses for transient hypoPT included extent of CND (bilateral vs unilateral), surgery (TT vs lobectomy), and surgery(TT vs sub-TT).

E

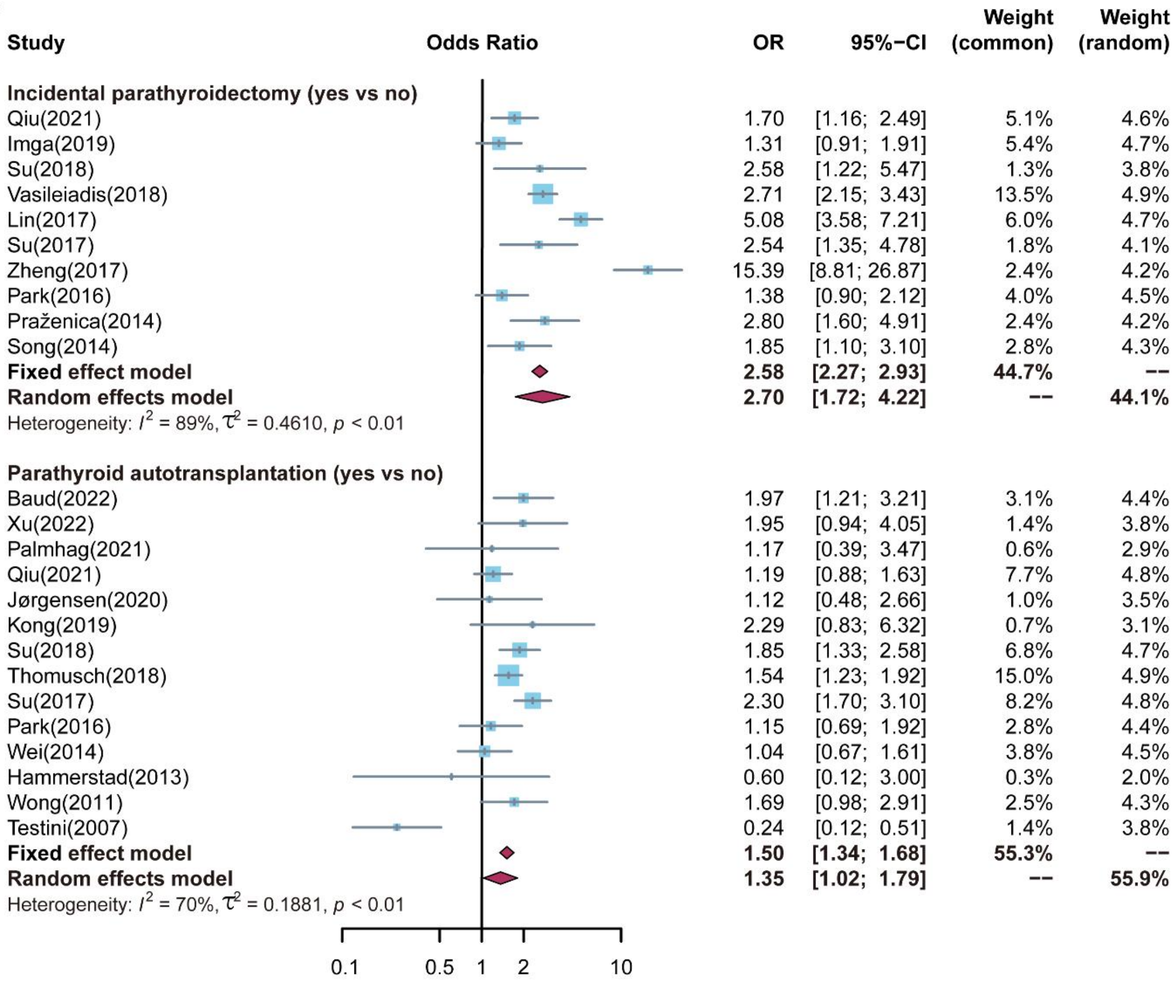

Figure S1E. Forest plots depicting the meta-analysis results of risk factors identified through univariable analyses for transient hypoPT included incidental parathyroidectomy, and parathyroid autotransplantation.

F

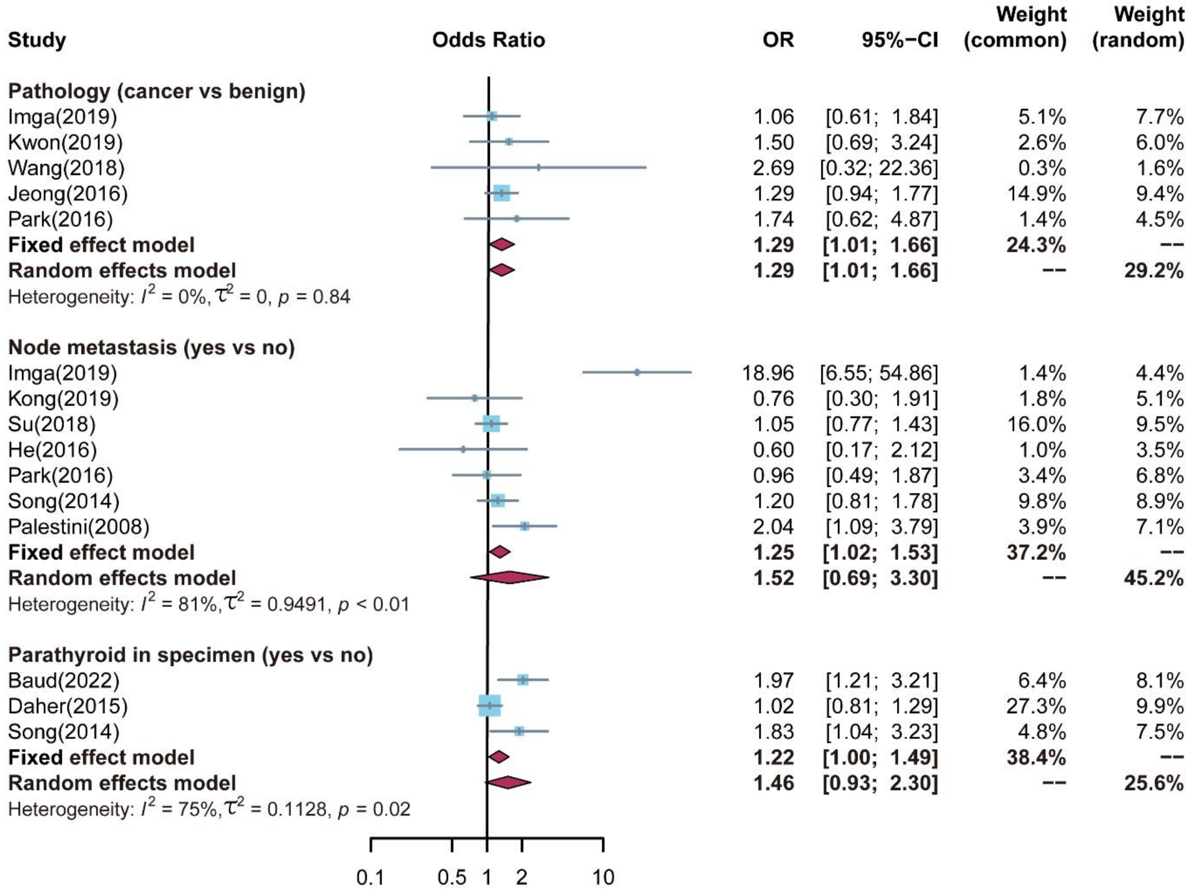

Figure S1F. Forest plots depicting the meta-analysis results of risk factors identified through univariable analyses for transient hypoPT included pathology (cancer vs benign), node metastasis and parathyroid in specimen.

Abbreviation: cN: clinical N; CND: central neck dissection; hypoPT: hypoparathyroidism; LND: lateral neck dissection; PTH: parathyroid hormone; TT: total thyroidectomy.

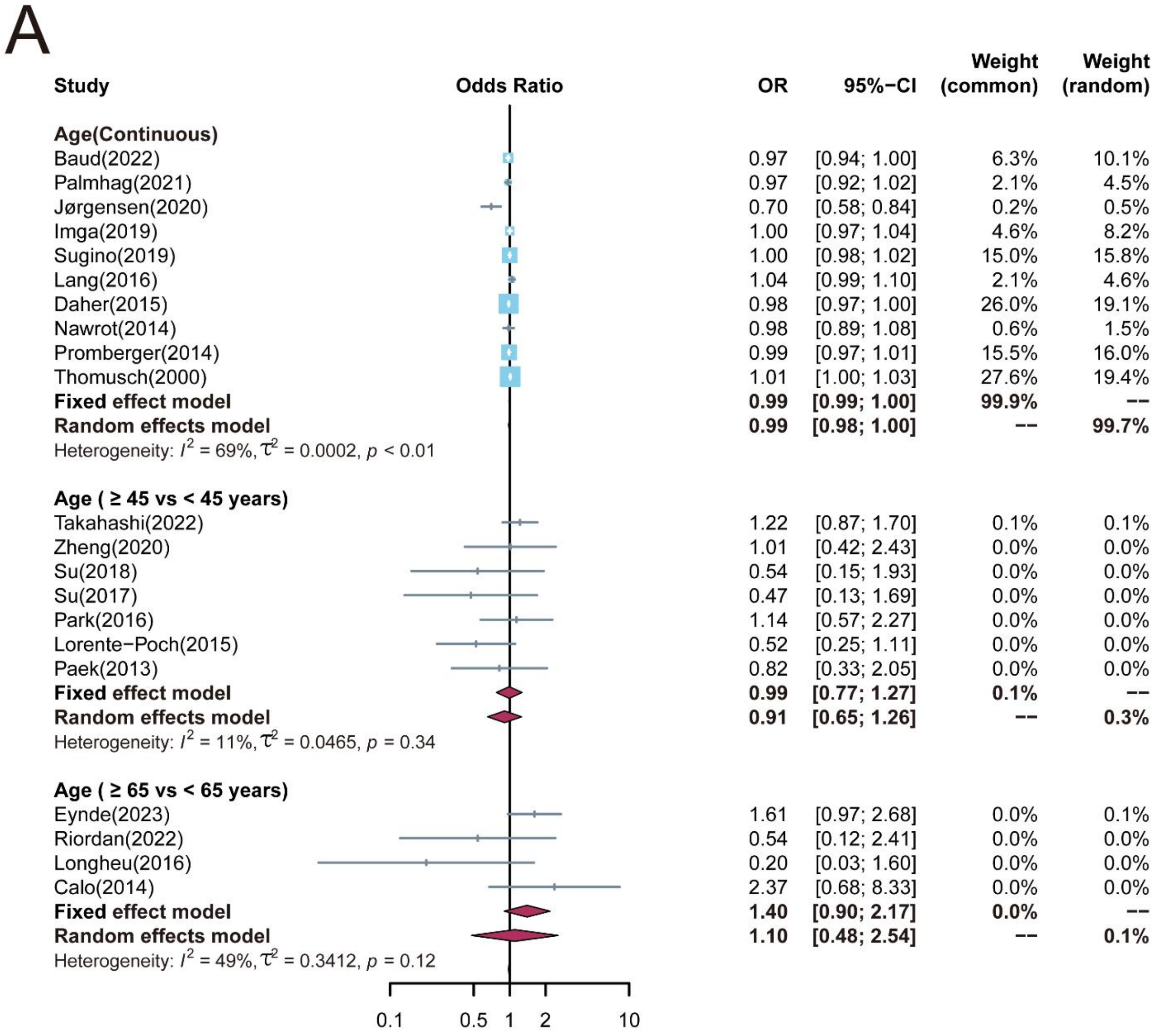

Figure S2A. Forest plots depicting the meta-analysis results of risk factors identified through univariable analyses for permanent hypoPT included age(continuous), age (≥ 45 vs < 45 years), and age (≥ 65 vs < 65 years).

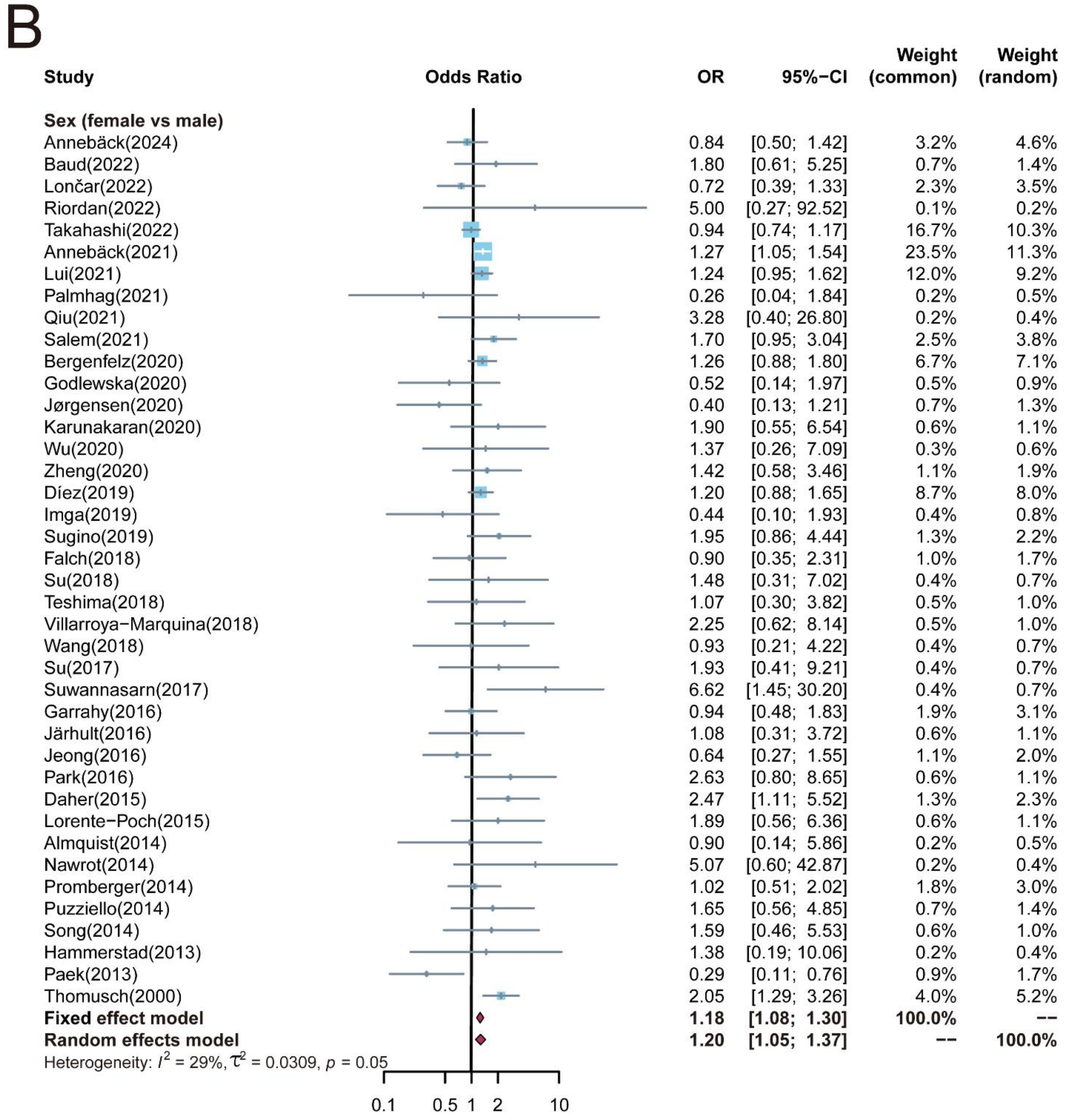

Figure S2B. Forest plots depicting the meta-analysis results of risk factors identified through univariable analyses for permanent hypoPT included sex (female vs male).

C

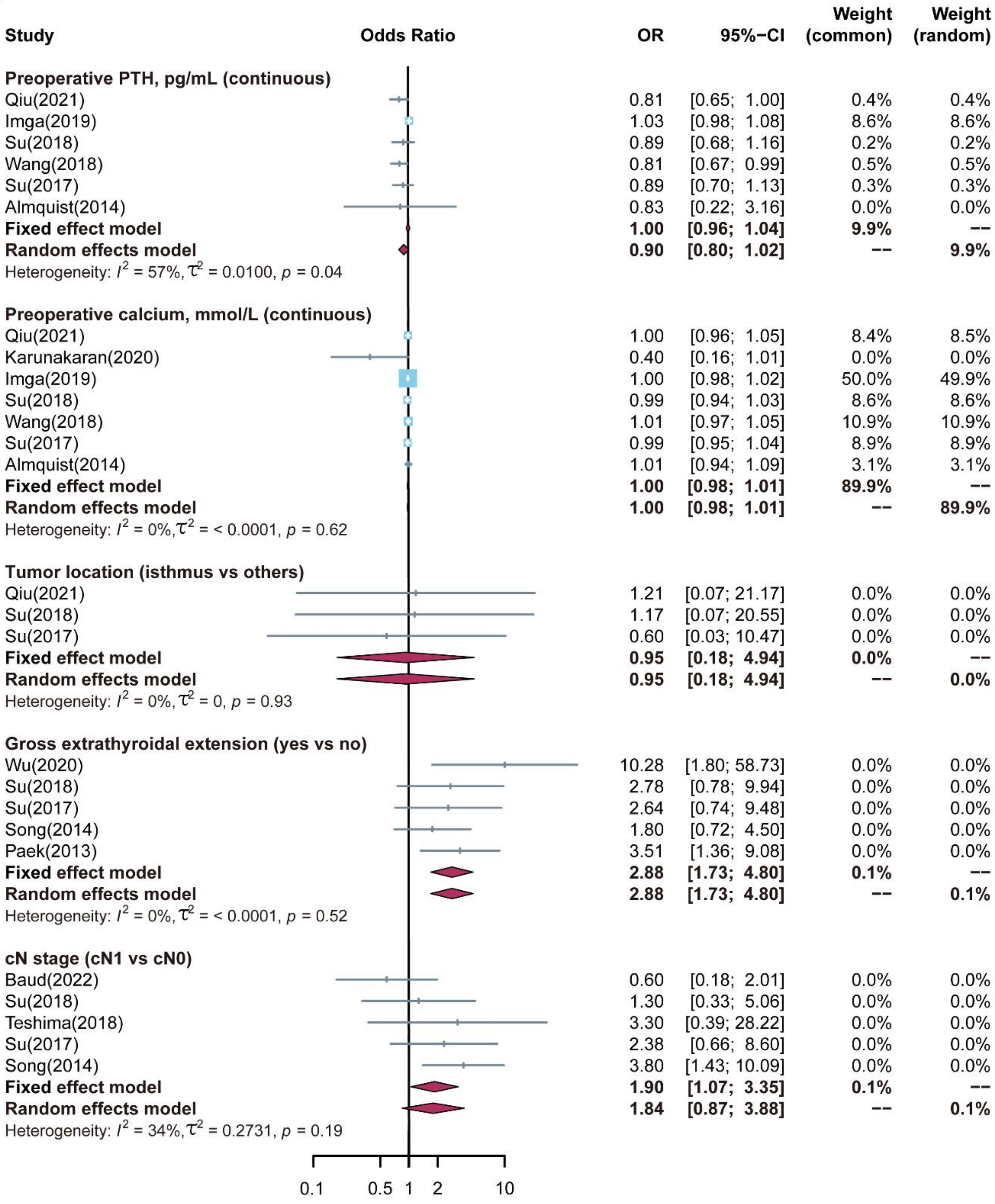

Figure S2C. Forest plots depicting the meta-analysis results of risk factors identified through univariable analyses for permanent hypoPT included preoperative PTH, preoperative calcium, tumor location (isthmus vs others), gross extrathyroidal extension, and cN stage (cN1 vs cN0).

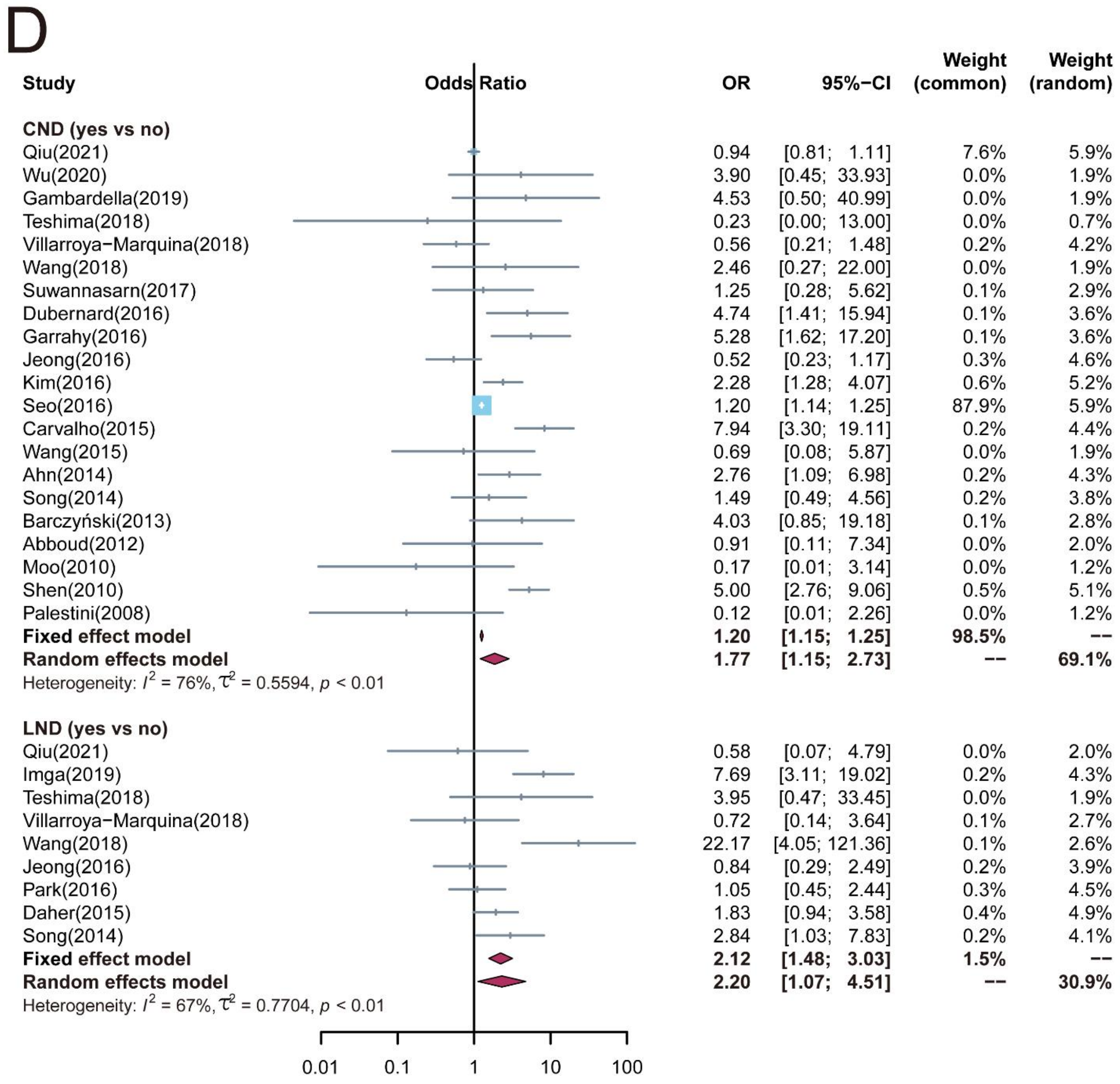

Figure S2D. Forest plots depicting the meta-analysis results of risk factors identified through univariable analyses for permanent hypoPT included CND and LND.

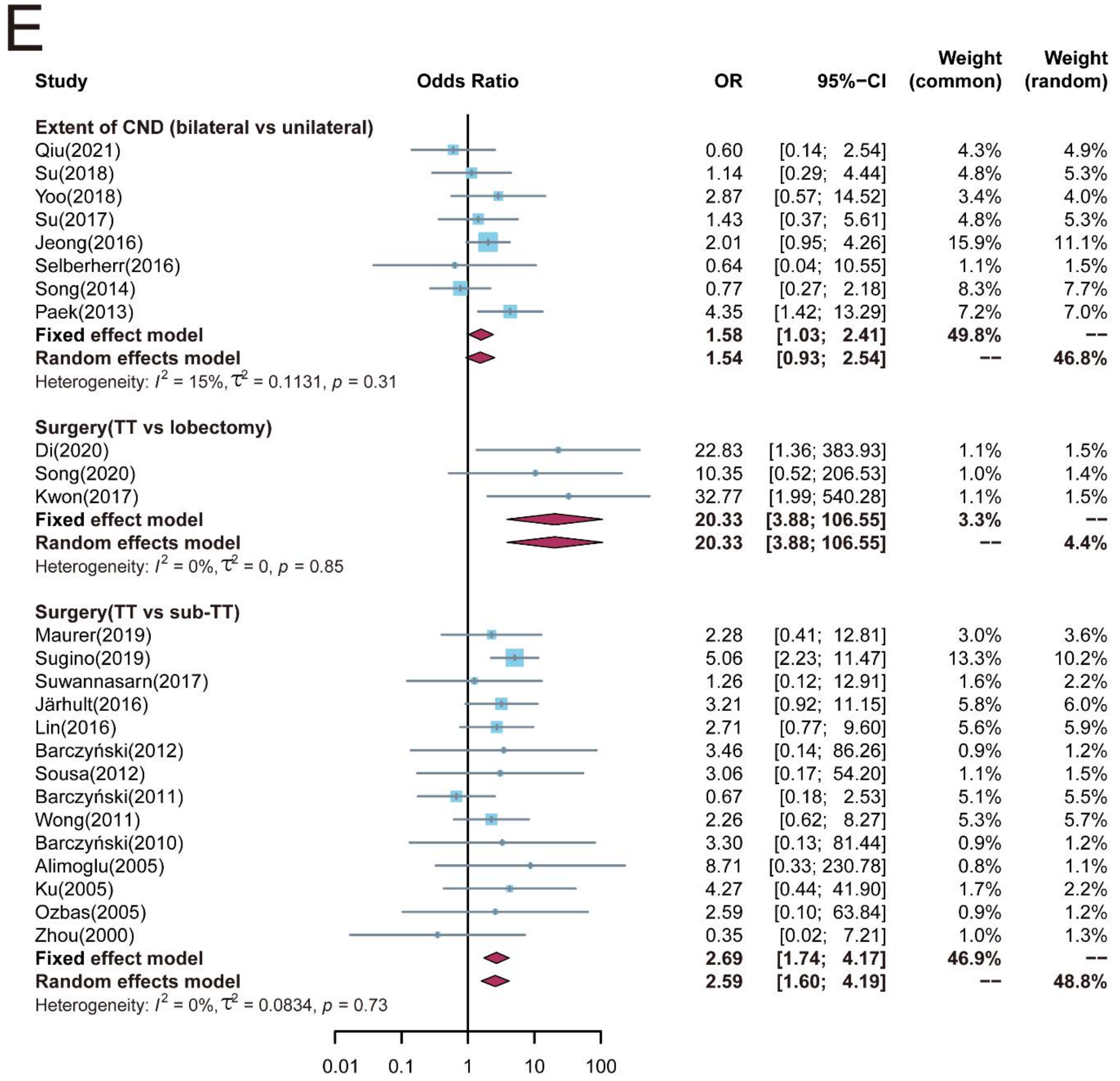

Figure S2E. Forest plots depicting the meta-analysis results of risk factors identified through univariable analyses for permanent hypoPT included extent of CND (bilateral vs unilateral), surgery (TT vs lobectomy), and surgery (TT vs sub-TT).

.

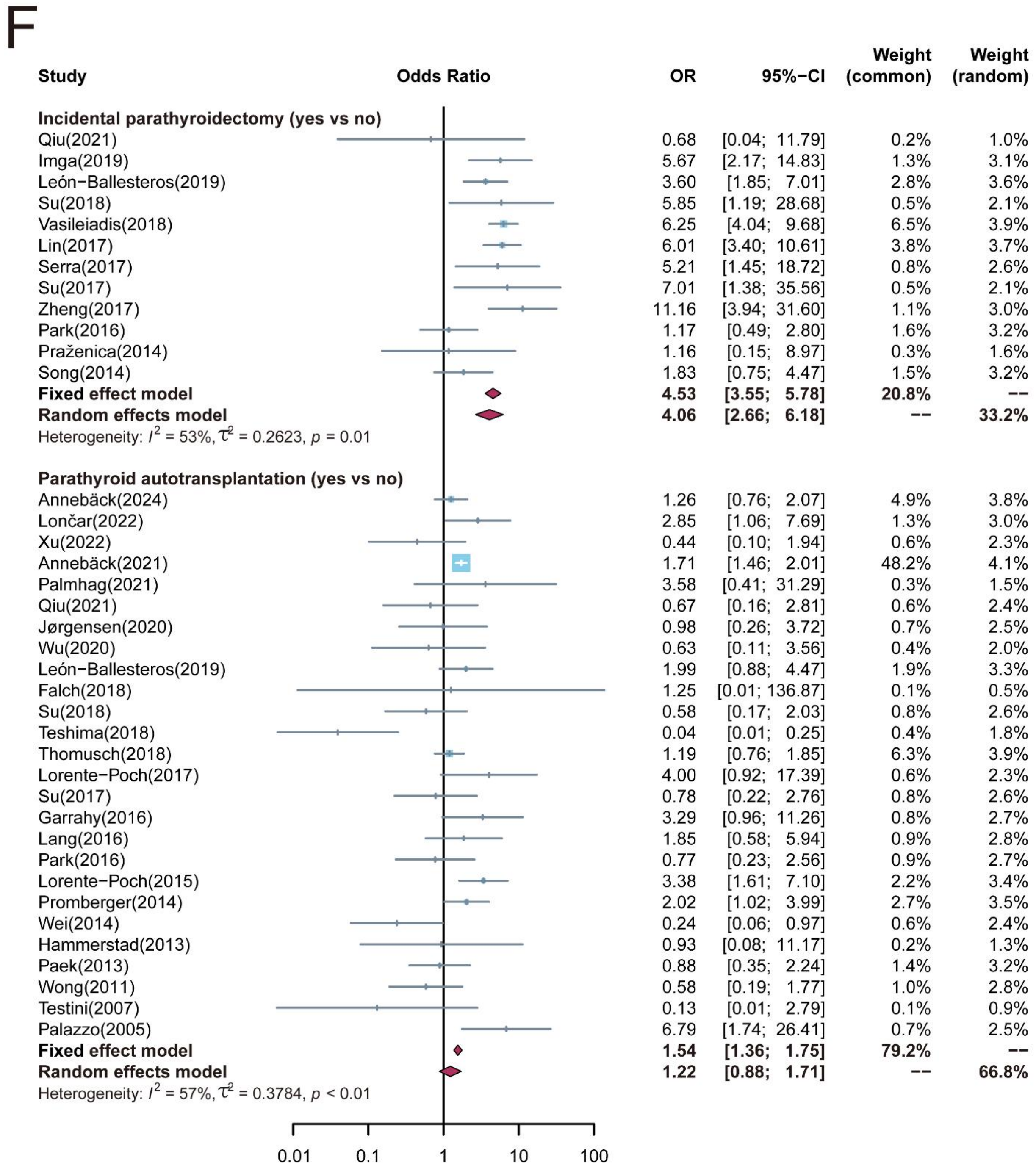

Figure S2F. Forest plots depicting the meta-analysis results of risk factors identified through univariable analyses for permanent hypoPT included incidental parathyroidectomy and parathyroid autotransplantation.

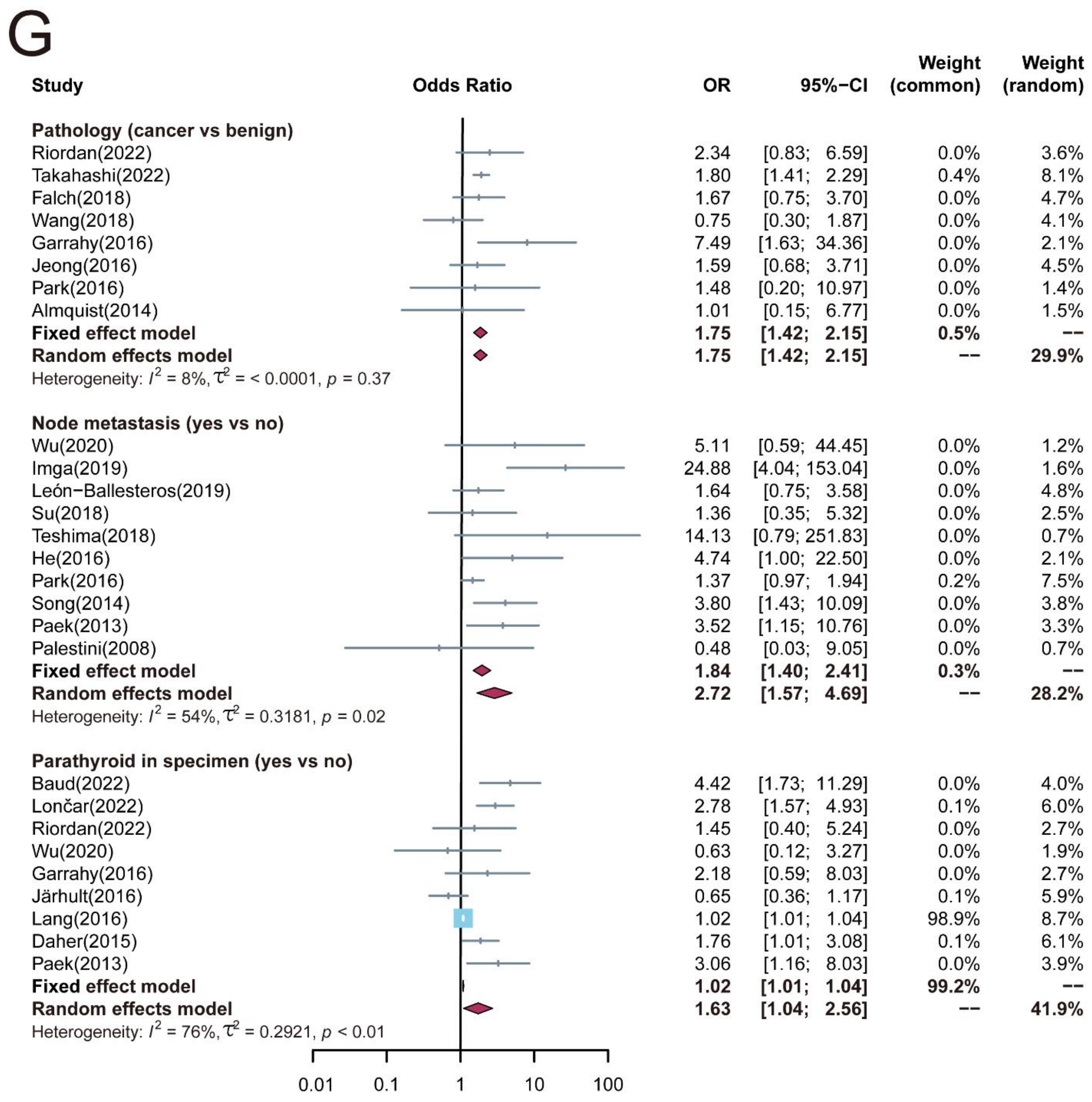

Figure S2G. Forest plots depicting the meta-analysis results of risk factors identified through univariable analyses for permanent hypoPT included pathology (cancer vs benign), node metastasis, and parathyroid in specimen.

Abbreviation: cN: clinical N; CND: central neck dissection; hypoPT; hypoparathyroidism; LND: lateral neck dissection; PTH: parathyroid hormone; TT: total thyroidectomy.

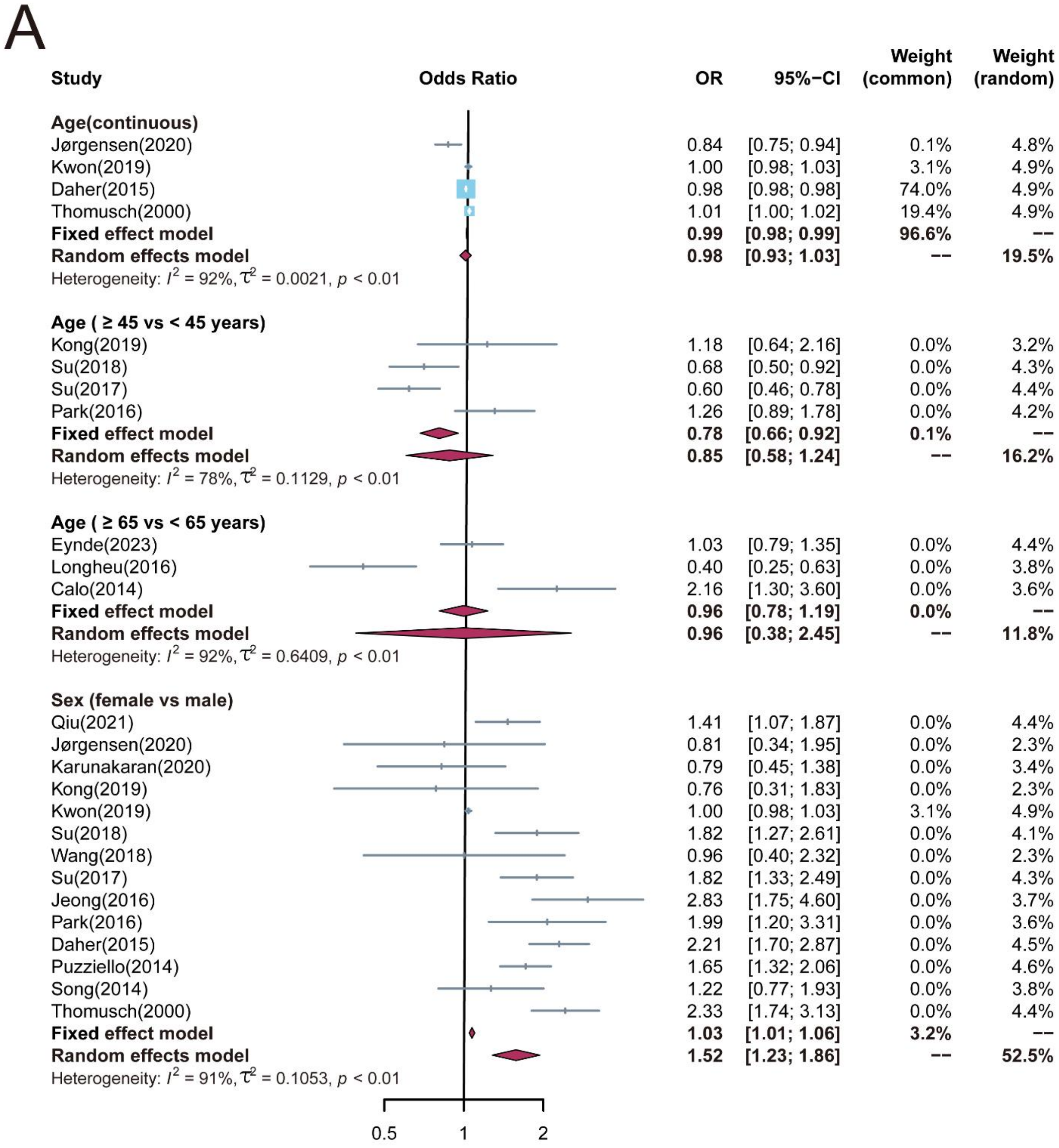

Figure S3A. Forest plots depicting the subgroup meta-analysis results of risk factors for transient hypoPT with 6-months definition time included age(continuous), age (≥ 45 vs < 45 years), age (≥ 65 vs < 65 years) and sex (female vs male).

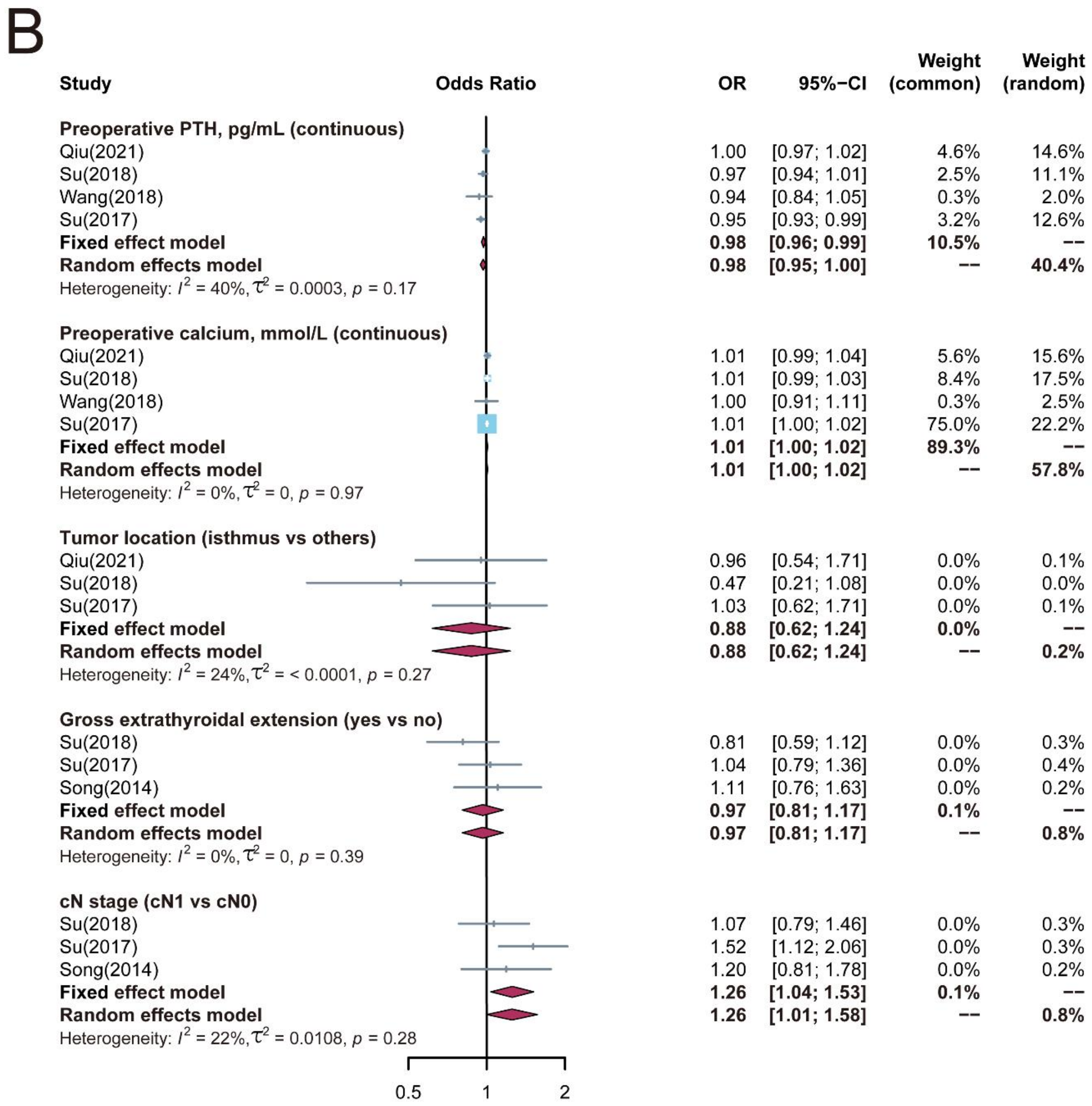

Figure S3B. Forest plots depicting the subgroup meta-analysis results of risk factors for transient hypoPT with 6-months definition time included preoperative PTH, preoperative calcium, tumor location (isthmus vs others), gross extrathyroidal extension, and cN stage.

C

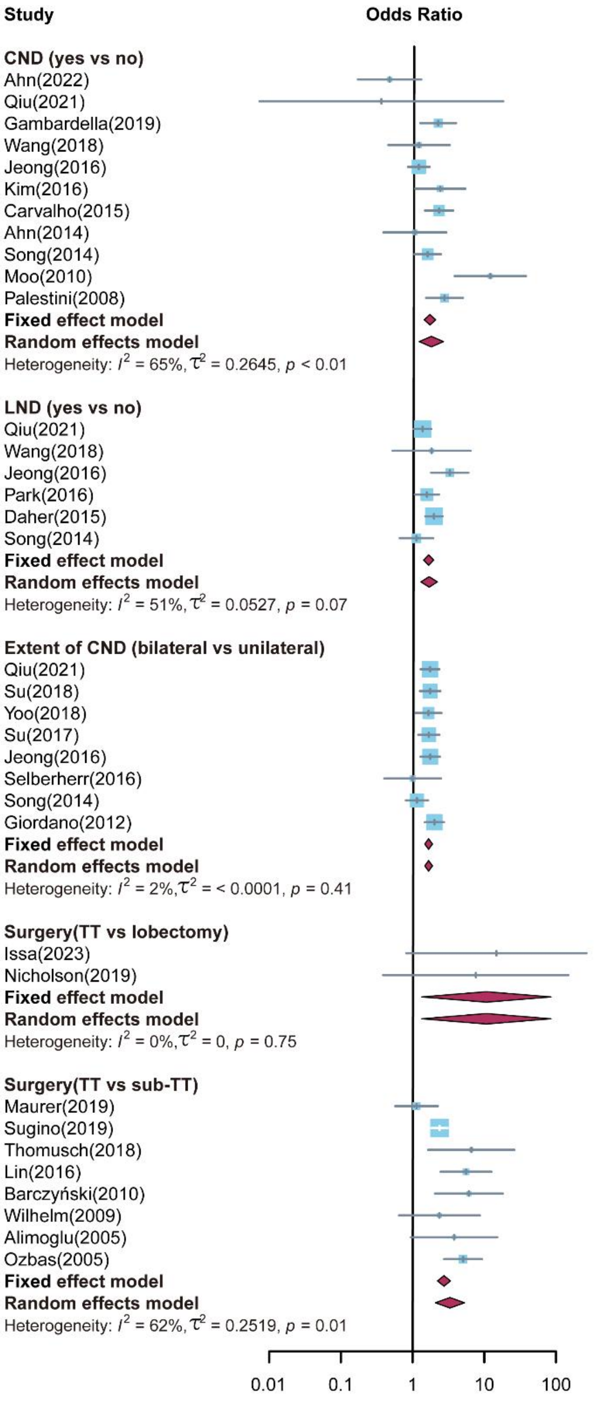

Study	OR	95%-CI	Weight (common)	Weight (random)
**CND (yes vs no)**				
Ahn(2022)	0.45	[0.16; 1.25]	0.6%	1.6%
Qiu(2021)	0.35	[0.01; 17.44]	0.0%	0.2%
Gambardella(2019)	2.16	[1.22; 3.83]	1.9%	3.2%
Wang(2018)	1.16	[0.43; 3.13]	0.7%	1.7%
Jeong(2016)	1.16	[0.82; 1.64]	5.2%	4.4%
Kim(2016)	2.31	[1.03; 5.16]	1.0%	2.2%
Carvalho(2015)	2.22	[1.41; 3.51]	3.1%	3.8%
Ahn(2014)	1.02	[0.37; 2.81]	0.6%	1.7%
Song(2014)	1.54	[1.00; 2.35]	3.5%	4.0%
Moo(2010)	11.51	[3.66; 36.23]	0.5%	1.4%
Palestini(2008)	2.64	[1.46; 4.79]	1.8%	3.1%
**Fixed effect model**	**1.66**	**[1.38; 1.99]**	**19.0%**	**--**
**Random effects model**	**1.73**	**[1.17; 2.56]**	**--**	**27.3%**
Heterogeneity: I^2 = 65%, τ^2 = 0.2645, p < 0.01				
**LND (yes vs no)**				
Qiu(2021)	1.31	[0.98; 1.74]	7.9%	4.8%
Wang(2018)	1.75	[0.50; 6.10]	0.4%	1.2%
Jeong(2016)	3.13	[1.72; 5.70]	1.8%	3.1%
Park(2016)	1.50	[1.02; 2.21]	4.2%	4.2%
Daher(2015)	1.88	[1.42; 2.48]	8.2%	4.8%
Song(2014)	1.07	[0.63; 1.84]	2.2%	3.4%
**Fixed effect model**	**1.59**	**[1.35; 1.87]**	**24.7%**	**--**
**Random effects model**	**1.61**	**[1.24; 2.10]**	**--**	**21.4%**
Heterogeneity: I^2 = 51%, τ^2 = 0.0527, p = 0.07				
**Extent of CND (bilateral vs unilateral)**				
Qiu(2021)	1.66	[1.23; 2.24]	7.0%	4.7%
Su(2018)	1.66	[1.20; 2.31]	5.9%	4.5%
Yoo(2018)	1.58	[1.04; 2.40]	3.6%	4.0%
Su(2017)	1.60	[1.14; 2.24]	5.5%	4.5%
Jeong(2016)	1.67	[1.22; 2.28]	6.6%	4.6%
Selberherr(2016)	0.95	[0.38; 2.36]	0.8%	1.9%
Song(2014)	1.09	[0.76; 1.56]	5.0%	4.4%
Giordano(2012)	1.91	[1.41; 2.60]	6.9%	4.7%
**Fixed effect model**	**1.59**	**[1.40; 1.79]**	**41.3%**	**--**
**Random effects model**	**1.59**	**[1.40; 1.79]**	**--**	**33.3%**
Heterogeneity: I^2 = 2%, τ^2 = < 0.0001, p = 0.41				
**Surgery(TT vs lobectomy)**				
Issa(2023)	13.97	[0.78; 251.53]	0.1%	0.3%
Nicholson(2019)	7.21	[0.37; 141.48]	0.1%	0.3%
**Fixed effect model**	**10.14**	**[1.27; 80.62]**	**0.1%**	**--**
**Random effects model**	**10.14**	**[1.27; 80.62]**	**--**	**0.5%**
Heterogeneity: I^2 = 0%, τ^2 = 0, p = 0.75				
**Surgery(TT vs sub-TT)**				
Maurer(2019)	1.08	[0.54; 2.13]	1.4%	2.7%
Sugino(2019)	2.25	[1.73; 2.93]	9.2%	4.9%
Thomusch(2018)	6.25	[1.56; 25.00]	0.3%	1.0%
Lin(2016)	5.27	[2.34; 11.92]	1.0%	2.2%
Barczyński(2010)	5.78	[1.94; 17.17]	0.5%	1.5%
Wilhelm(2009)	2.24	[0.61; 8.22]	0.4%	1.1%
Alimoglu(2005)	3.60	[0.90; 14.40]	0.3%	1.0%
Ozbas(2005)	4.80	[2.61; 8.82]	1.7%	3.0%
**Fixed effect model**	**2.60**	**[2.11; 3.20]**	**14.8%**	**--**
**Random effects model**	**3.15**	**[1.97; 5.03]**	**--**	**17.5%**
Heterogeneity: I^2 = 62%, τ^2 = 0.2519, p = 0.01				

Figure S3C. Forest plots depicting the subgroup meta-analysis results of risk factors for transient hypoPT with 6-months definition time included CND, LND, extent of CND (bilateral vs unilateral), surgery (TT vs lobectomy) and surgery (TT vs sub-TT).

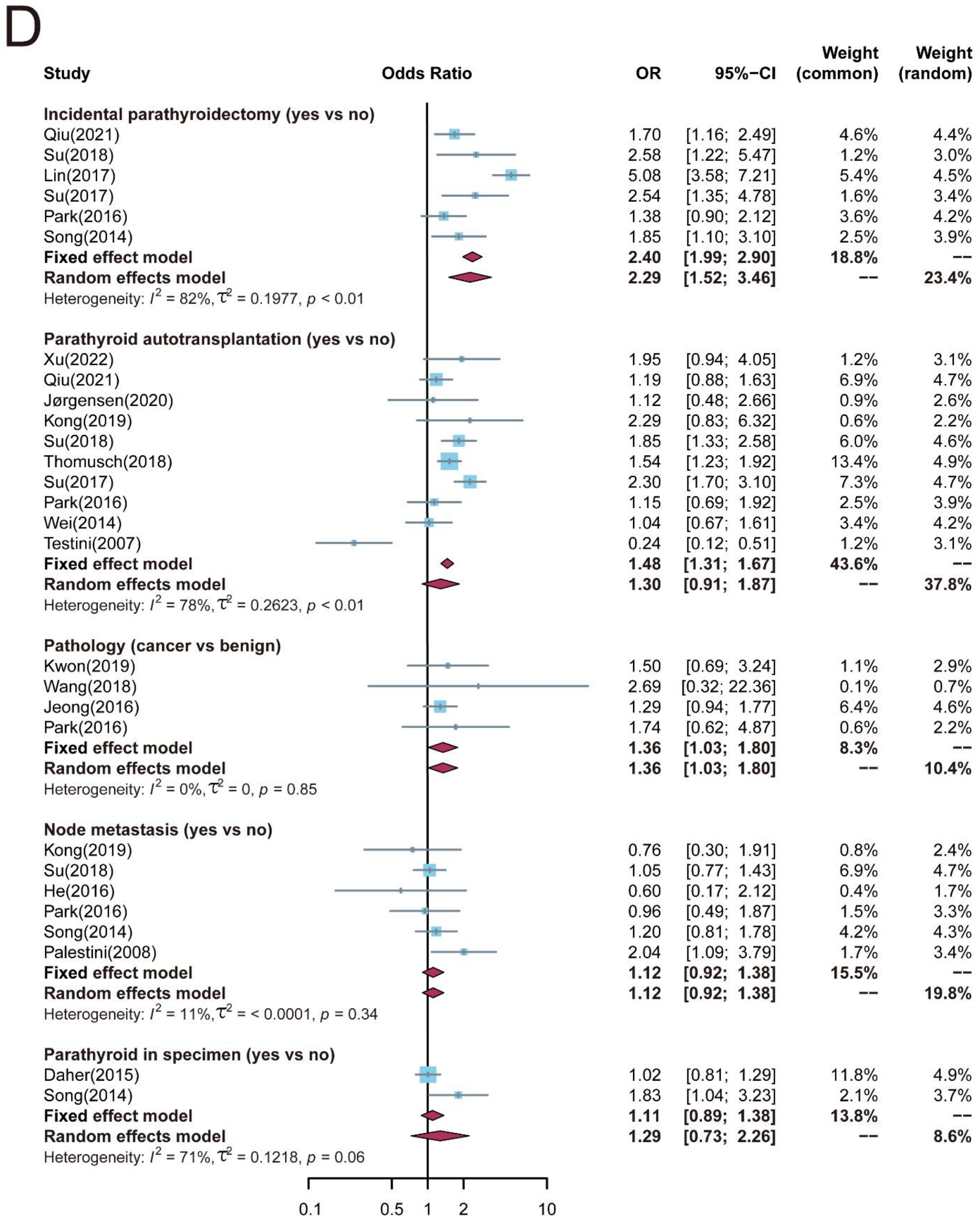

Figure S3D. Forest plots depicting the subgroup meta-analysis results of risk factors for transient hypoPT with 6-months definition time included incidental parathyroidectomy, parathyroid autotransplantation, pathology (cancer vs benign), node metastasis, and parathyroid in specimen.

Abbreviation: cN: clinical N; CND: central neck dissection; hypoPT: hypoparathyroidism; LND: lateral neck dissection; PTH: parathyroid hormone; TT: total thyroidectomy.

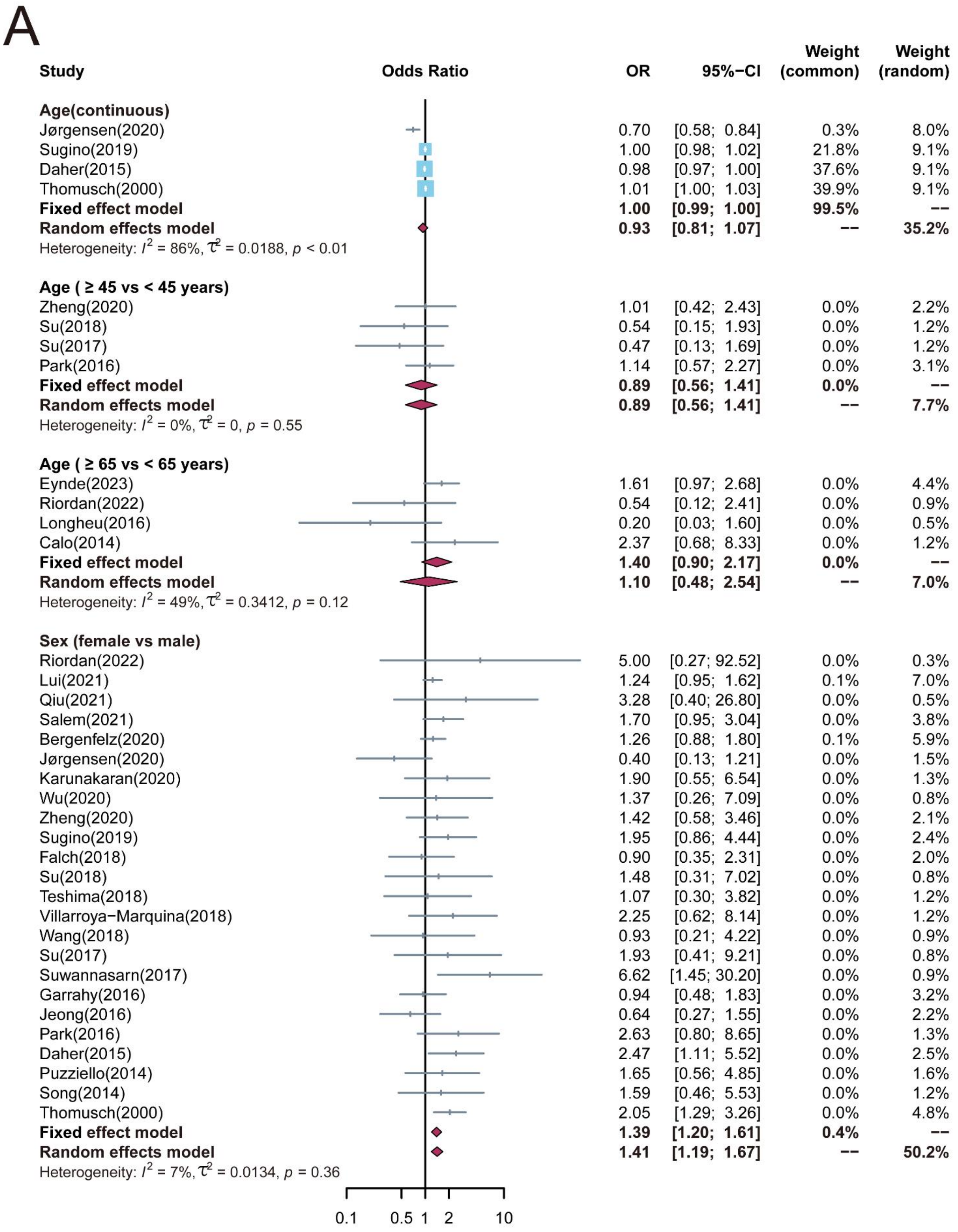

Figure S4A. Forest plots depicting the subgroup meta-analysis results of risk factors for permanent hypoPT with 6-months definition time included age(continuous), age (≥ 45 vs < 45 years), age (≥ 65 vs < 65 years), sex (female vs male).

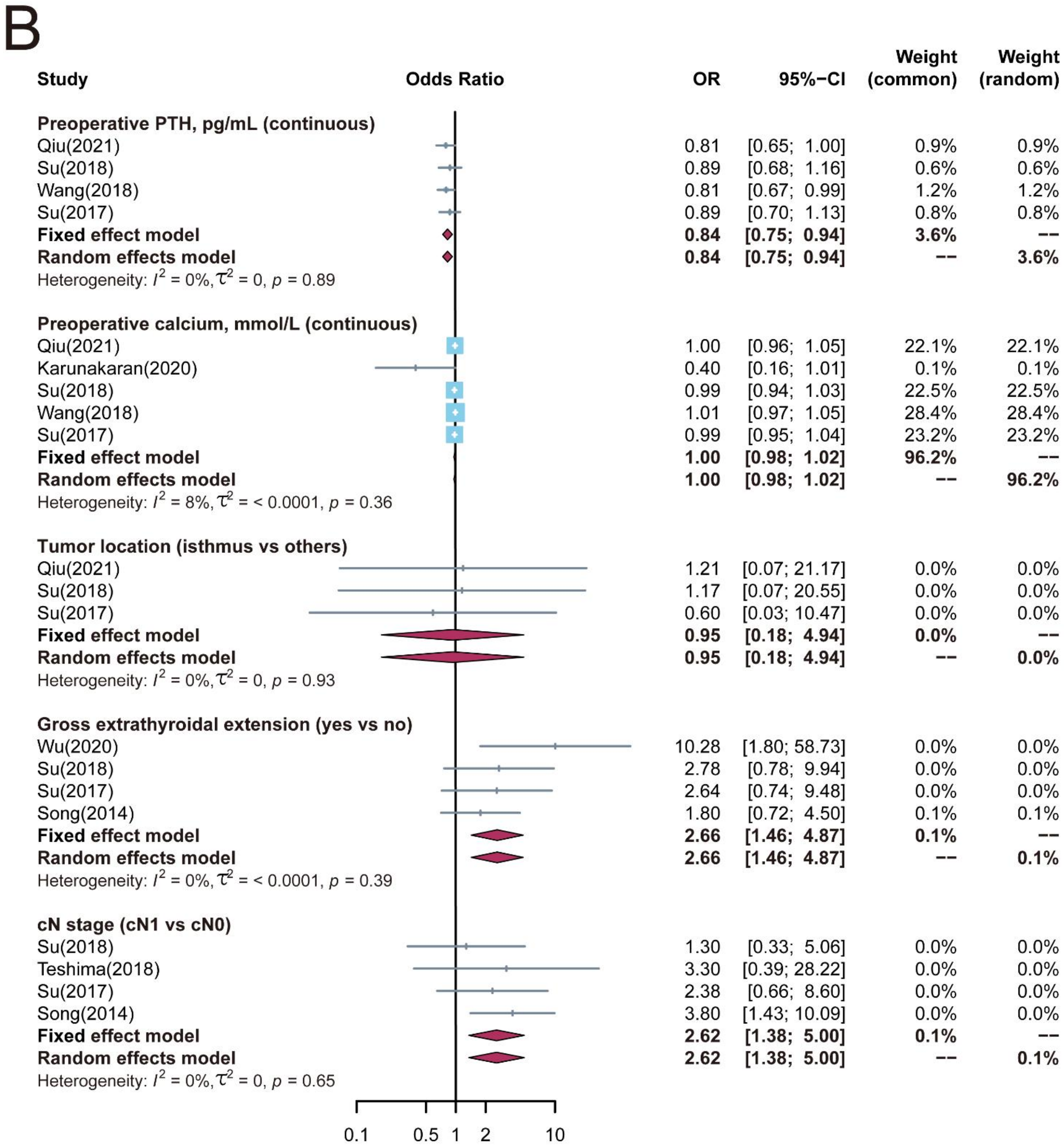

Figure S4B. Forest plots depicting the subgroup meta-analysis results of risk factors for permanent hypoPT with 6-months definition time included preoperative PTH, preoperative calcium, tumor location (isthmus vs others), gross extrathyroidal extension, and cN stage.

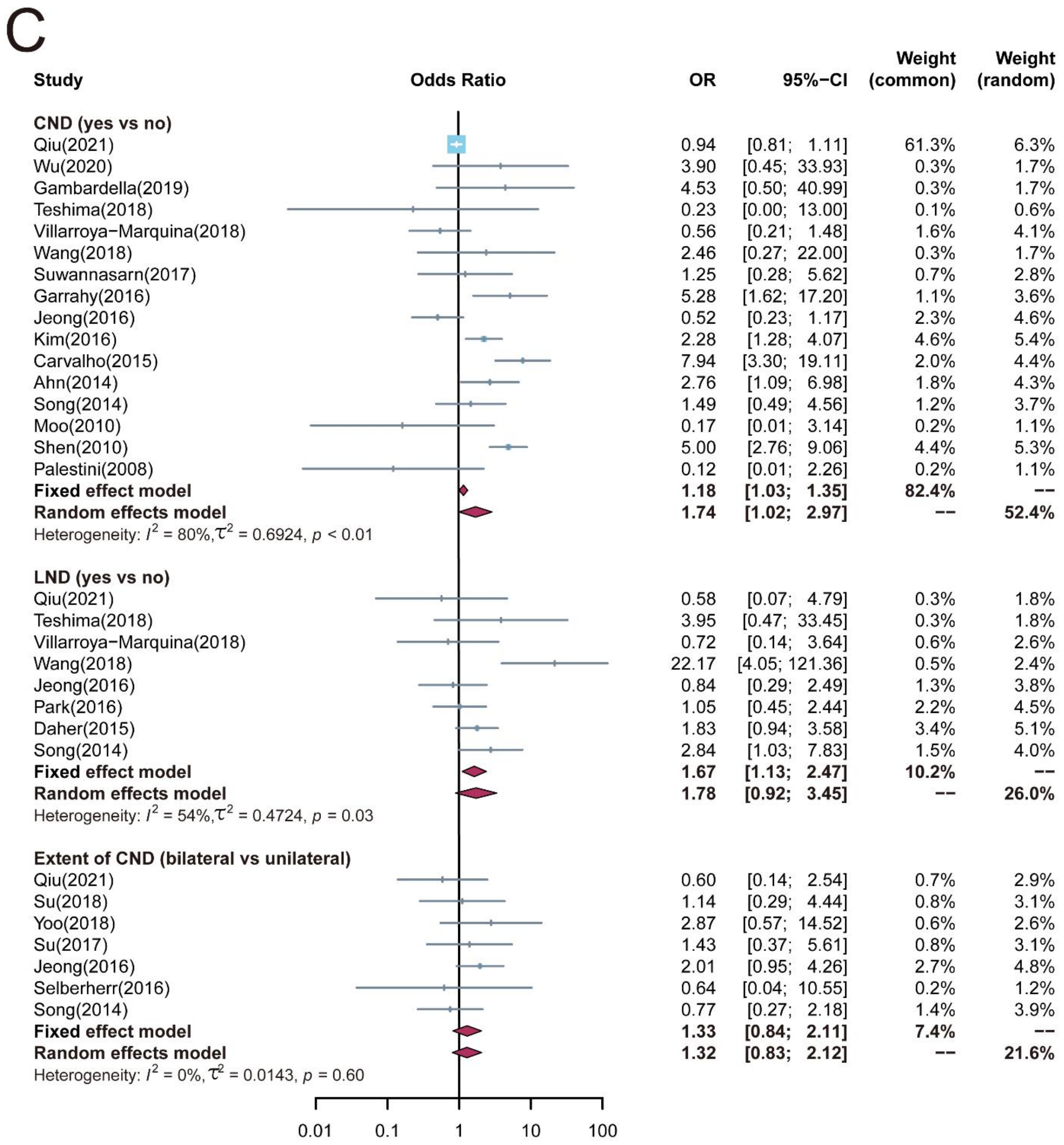

Figure S4C. Forest plots depicting the subgroup meta-analysis results of risk factors for permanent hypoPT with 6-months definition time included CND, LND, and extent of CND (bilateral vs unilateral).

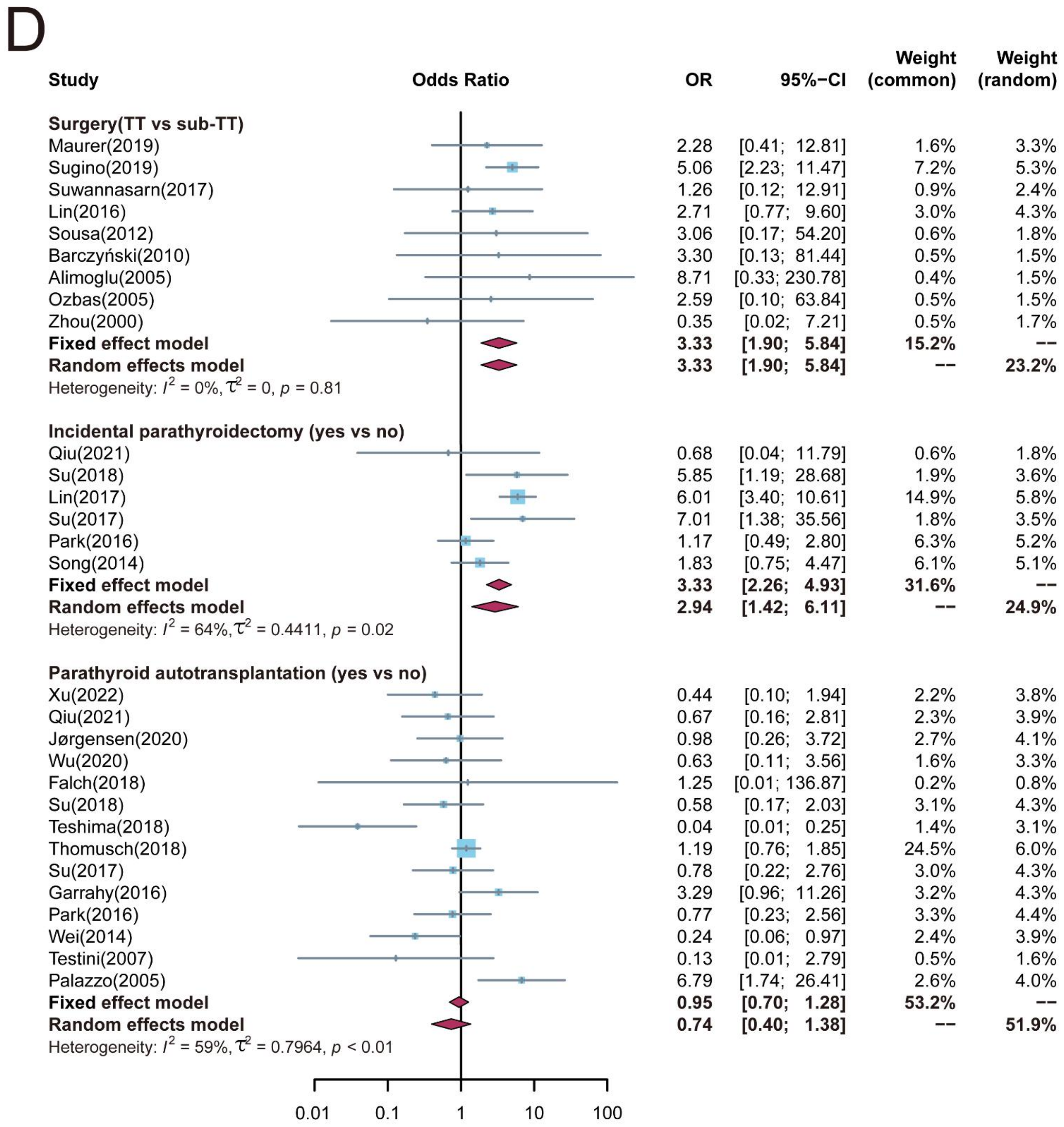

Figure S4D. Forest plots depicting the subgroup meta-analysis results of risk factors for permanent hypoPT with 6-months definition time included surgery (TT vs sun-TT), incidental parathyroidectomy, and parathyroid autotransplantation.

E

Study	OR	95%−CI	Weight (common)	Weight (random)
**Pathology (cancer vs benign)**				
Riordan(2022)	2.34	[0.83; 6.59]	4.4%	4.9%
Falch(2018)	1.67	[0.75; 3.70]	7.4%	8.1%
Wang(2018)	0.75	[0.30; 1.87]	5.6%	6.2%
Garrahy(2016)	7.49	[1.63; 34.36]	2.0%	2.3%
Jeong(2016)	1.59	[0.68; 3.71]	6.6%	7.2%
Park(2016)	1.48	[0.20; 10.97]	1.2%	1.4%
**Fixed effect model**	**1.64**	**[1.08; 2.49]**	**27.1%**	**--**
**Random effects model**	**1.68**	**[1.04; 2.73]**	**--**	**30.2%**
Heterogeneity: $I^2 = 30\%$, $\tau^2 = 0.0795$, $p = 0.21$				
**Node metastasis (yes vs no)**				
Wu(2020)	5.11	[0.59; 44.45]	1.0%	1.2%
Su(2018)	1.36	[0.35; 5.32]	2.5%	2.9%
Teshima(2018)	14.13	[0.79; 251.83]	0.6%	0.7%
He(2016)	4.74	[1.00; 22.50]	1.9%	2.2%
Park(2016)	1.37	[0.97; 1.94]	39.0%	32.9%
Song(2014)	3.80	[1.43; 10.09]	4.9%	5.5%
Palestini(2008)	0.48	[0.03; 9.05]	0.5%	0.6%
**Fixed effect model**	**1.65**	**[1.22; 2.24]**	**50.5%**	**--**
**Random effects model**	**2.25**	**[1.22; 4.15]**	**--**	**46.0%**
Heterogeneity: $I^2 = 38\%$, $\tau^2 = 0.2171$, $p = 0.14$				
**Parathyroid in specimen (yes vs no)**				
Riordan(2022)	1.45	[0.40; 5.24]	2.8%	3.3%
Wu(2020)	0.63	[0.12; 3.27]	1.7%	2.0%
Garrahy(2016)	2.18	[0.59; 8.03]	2.8%	3.2%
Daher(2015)	1.76	[1.01; 3.08]	15.0%	15.3%
**Fixedeffect model**	**1.63**	**[1.03; 2.57]**	**22.3%**	**--**
**Random effects model**	**1.63**	**[1.03; 2.57]**	**--**	**23.7%**
Heterogeneity: $I^2 = 0\%$, $\tau^2 = 0$, $p = 0.66$				

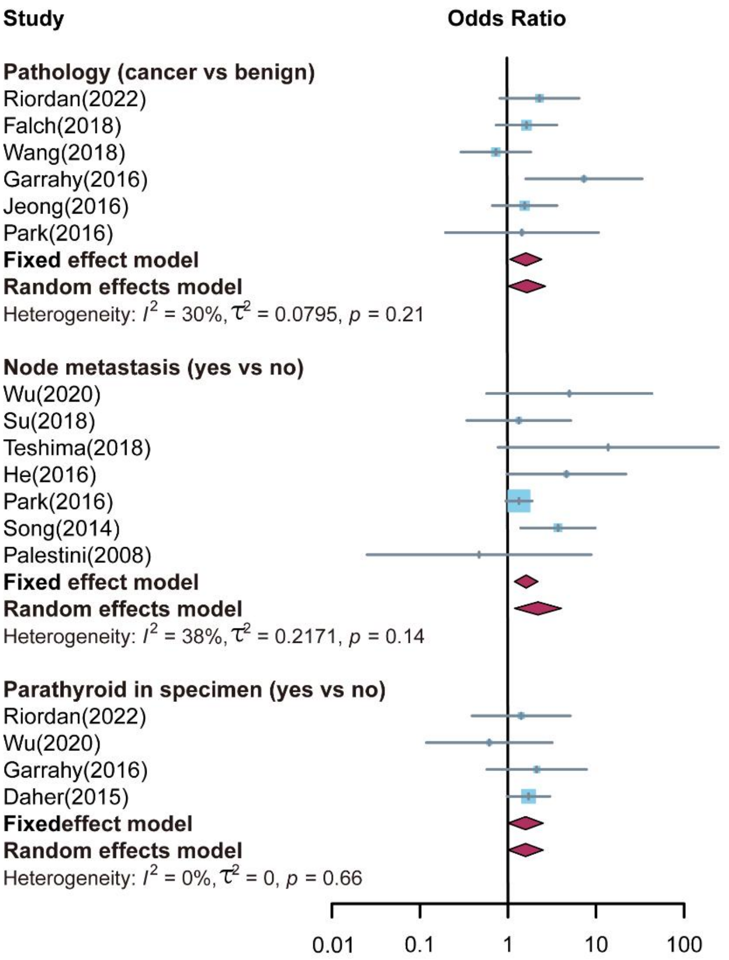

Figure S4E. Forest plots depicting the subgroup meta-analysis results of risk factors for permanent hypoPT with 6-months definition time included pathology (cancer vs benign), node metastasis and parathyroid in specimen.

Abbreviation: cN: clinical N; CND: central neck dissection; hypoPT: hypoparathyroidism; LND: lateral neck dissection; PTH: parathyroid hormone; TT: total thyroidectomy.

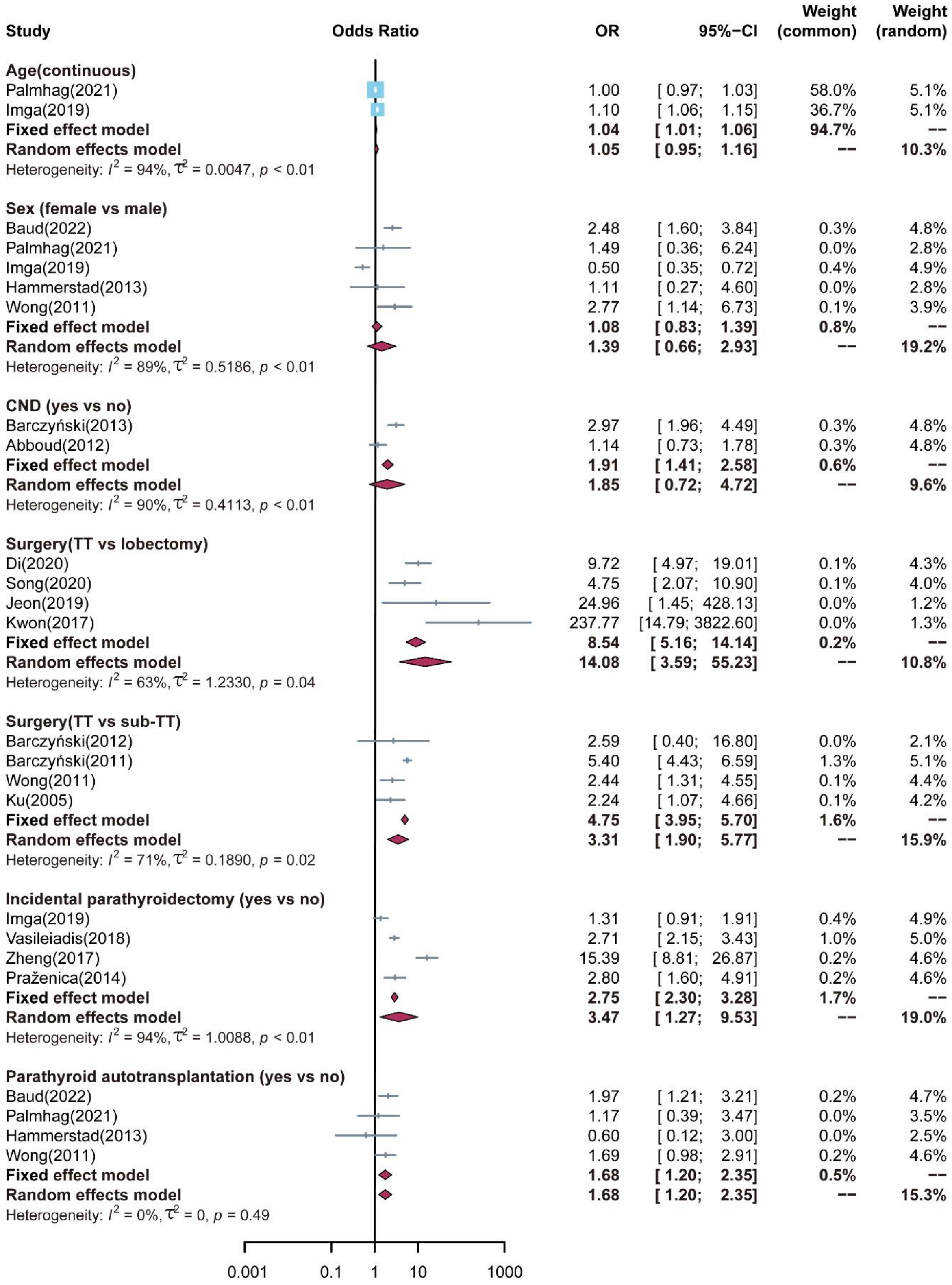

Figure S5. Forest plots depicting the subgroup meta-analysis results of risk factors for transient hypoPT with 12-months definition time included age(continuous), sex (female vs male), CND, surgery (TT vs lobectomy) and surgery (TT vs sub-TT), incidental parathyroidectomy, and parathyroid autotransplantation.

Abbreviation: cN: clinical N; CND: central neck dissection; hypoPT: hypoparathyroidism; LND: lateral neck dissection; PTH: parathyroid hormone; TT: total thyroidectomy.

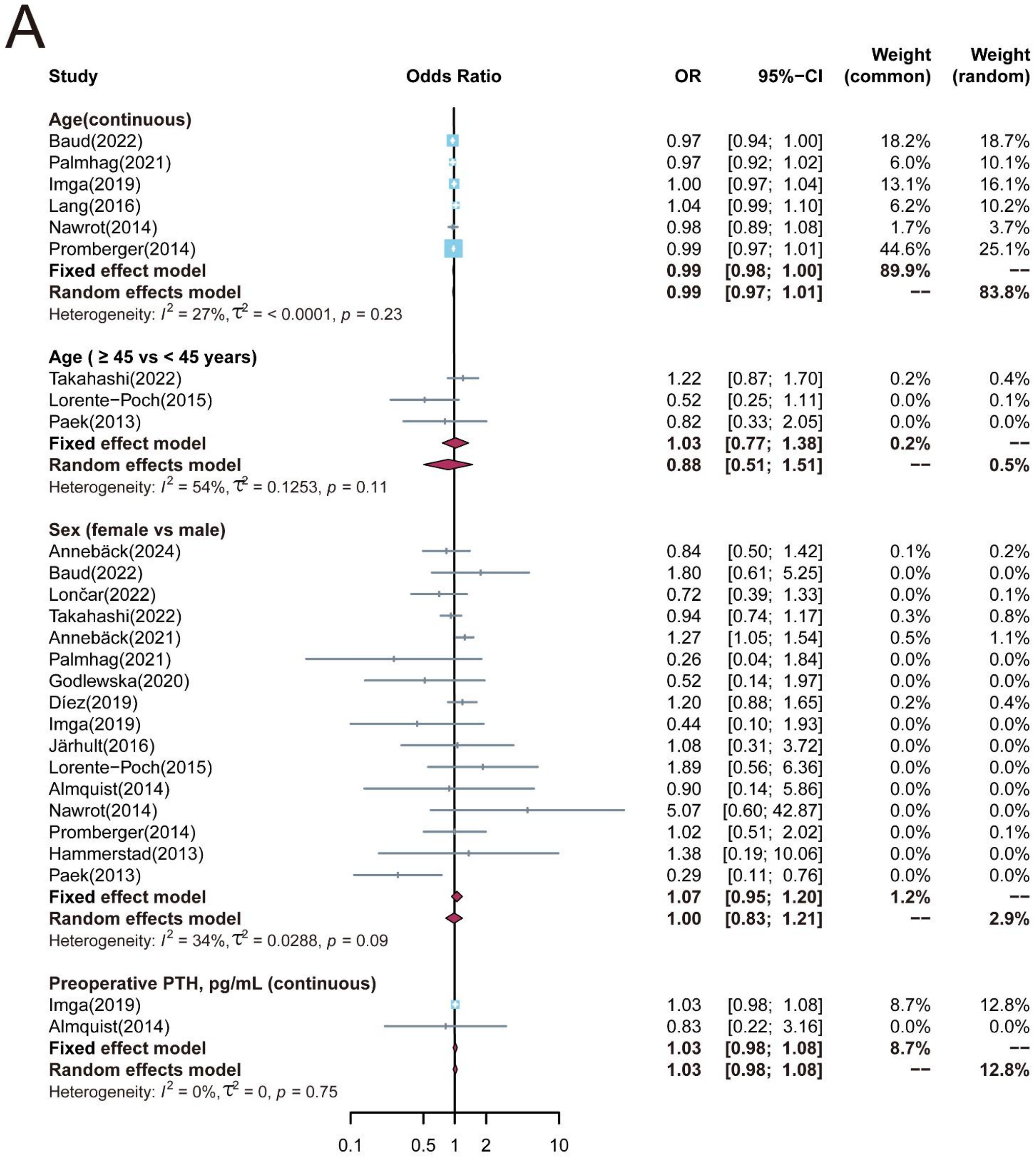

Figure S6A. Forest plots depicting the subgroup meta-analysis results of risk factors for permanent hypoPT with 12-months definition time included age(continuous), age (≥ 45 vs < 45 years), sex (female vs male), and preoperative PTH.

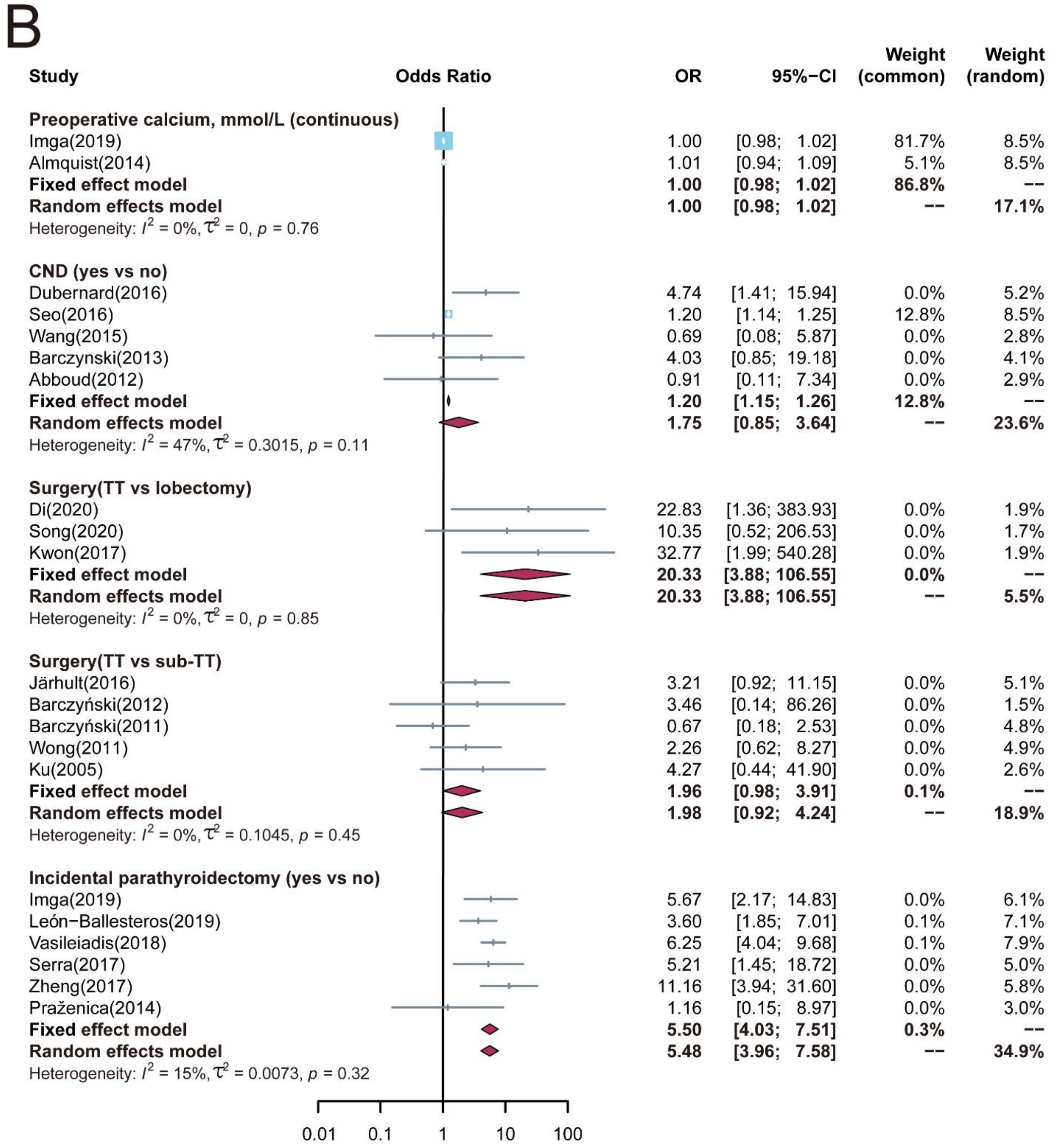

Figure S6B. Forest plots depicting the subgroup meta-analysis results of risk factors for permanent hypoPT with 12-months definition time included preoperative calcium, CND, surgery (TT vs lobectomy), surgery (TT vs sub-TT), and incidental parathyroidectomy.

C

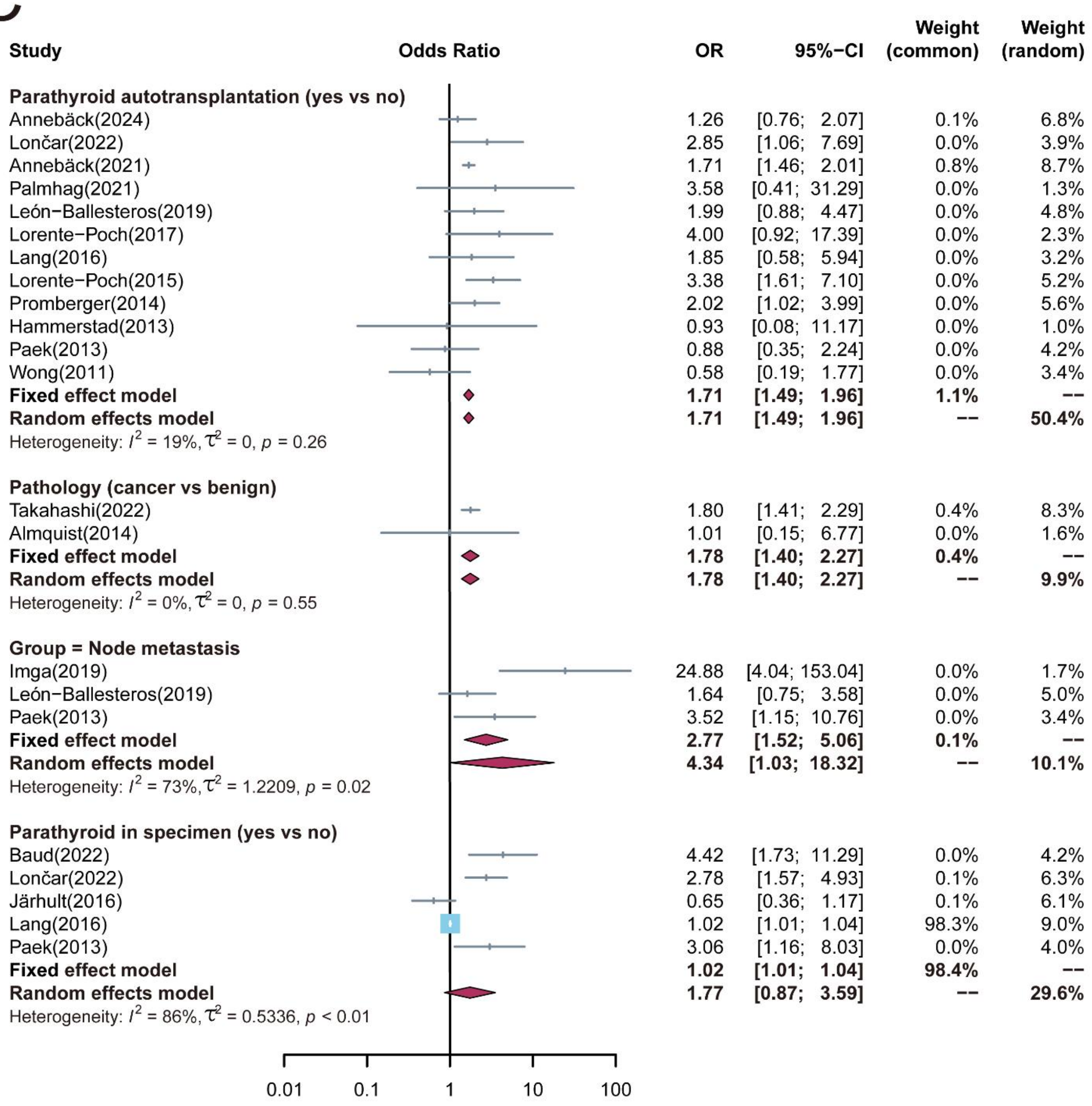

Figure S6C. Forest plots depicting the subgroup meta-analysis results of risk factors for permanent hypoPT with 12-months definition time included parathyroid autotransplantation, pathology (cancer vs benign), node metastasis, parathyroid in specimen.

Abbreviation: cN: clinical N; CND: central neck dissection; hypoPT: hypoparathyroidism; LND: lateral neck dissection; PTH: parathyroid hormone; TT: total thyroidectomy.
