# Supplementary material for: Risk factors of transient and permanent hypoparathyroidism after thyroidectomy: a systematic review and meta-analysis
Source: Int J Surg. 2024 Apr 23;110(8):5047–62. doi: 10.1097/JS9.0000000000001475 (PMC11326036; doi:10.1097/JS9.0000000000001475)
Supplement: Supplementary file 6 [file js9-110-5047-s006.docx]

**
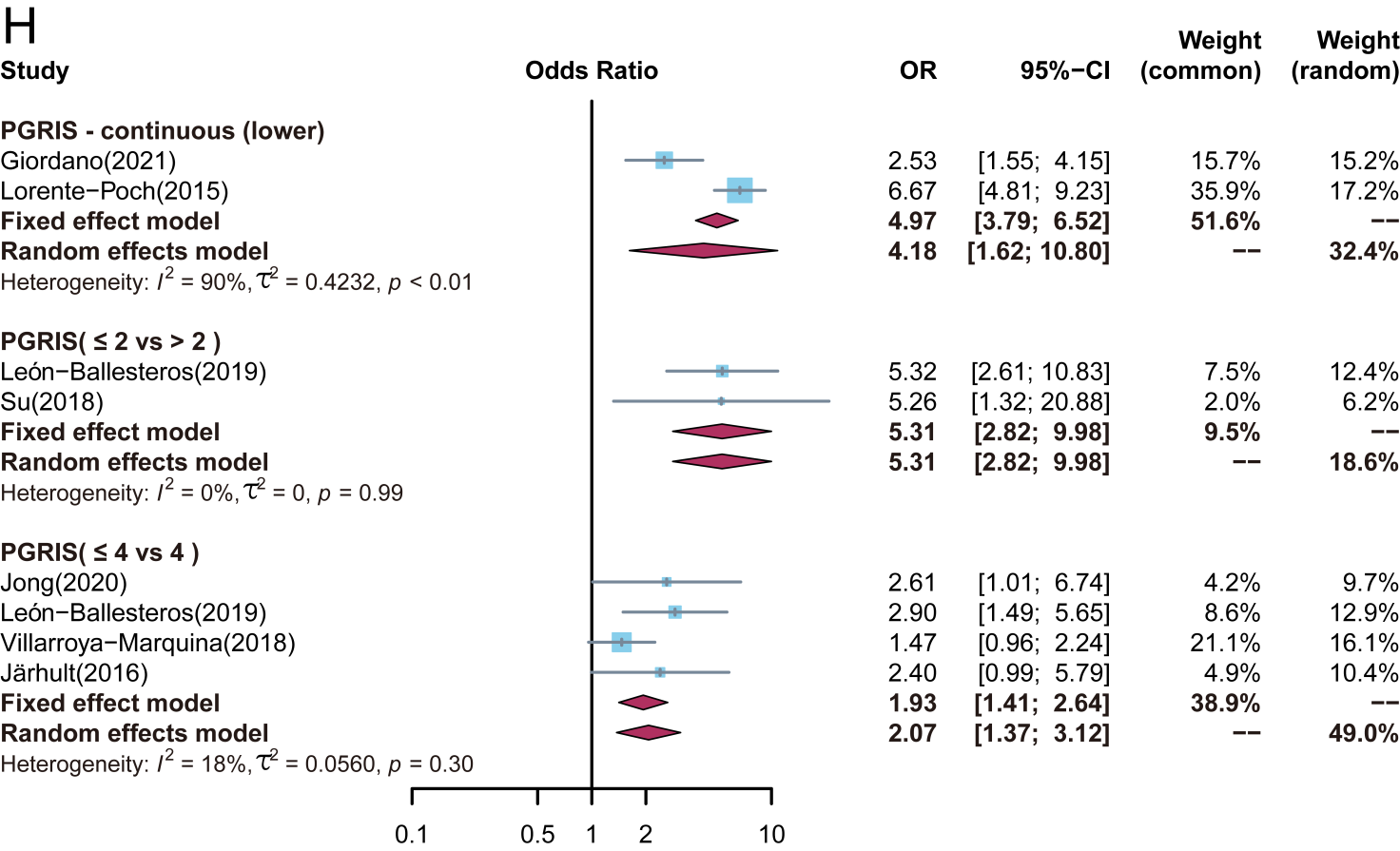
**

**Figure S2H.** Forest plots depicting the meta-analysis results of risk factors identified through univariable analyses for permanent hypoPT included PGRIS (continuous), PGRIS (≤ 2 vs > 2), and PGRIS (≤ 4 vs 4).

**Abbreviation:** cN: clinical N; CND: central neck dissection; LND: lateral neck dissection; PTH: parathyroid hormone; PGRIS: parathyroid glands remaining in situ; TT: total thyroidectomy; hypoPT; hypoparathyroidism.
